# Stem Cell-Like Gene Expression in Ovarian Cancer Predicts Type II Subtype and Prognosis

**DOI:** 10.1371/journal.pone.0057799

**Published:** 2013-03-11

**Authors:** Matthew Schwede, Dimitrios Spentzos, Stefan Bentink, Oliver Hofmann, Benjamin Haibe-Kains, David Harrington, John Quackenbush, Aedín C. Culhane

**Affiliations:** 1 Biostatistics and Computational Biology, Dana-Farber Cancer Institute, Boston, Massachusetts, United States of America; 2 Biostatistics, Harvard School of Public Health, Boston, Massachusetts, United States of America; 3 Division of Hematology/Oncology, Department of Medicine, Beth Israel Deaconess Medical Center, Boston, Massachusetts, United States of America; 4 Exosome Diagnostics GmbH, Martinsried, Germany; 5 Cancer Biology, Dana-Farber Cancer Institute, Boston, Massachusetts, United States of America; 6 Integrative Systems Biology, Institut de recherches cliniques de Montréal, Montreal, Quebec, Canada; Ohio State University Comprehensive Cancer Center, United States of America

## Abstract

Although ovarian cancer is often initially chemotherapy-sensitive, the vast majority of tumors eventually relapse and patients die of increasingly aggressive disease. Cancer stem cells are believed to have properties that allow them to survive therapy and may drive recurrent tumor growth. Cancer stem cells or cancer-initiating cells are a rare cell population and difficult to isolate experimentally. Genes that are expressed by stem cells may characterize a subset of less differentiated tumors and aid in prognostic classification of ovarian cancer. The purpose of this study was the genomic identification and characterization of a subtype of ovarian cancer that has stem cell-like gene expression. Using human and mouse gene signatures of embryonic, adult, or cancer stem cells, we performed an unsupervised bipartition class discovery on expression profiles from 145 serous ovarian tumors to identify a stem-like and more differentiated subgroup. Subtypes were reproducible and were further characterized in four independent, heterogeneous ovarian cancer datasets. We identified a stem-like subtype characterized by a 51-gene signature, which is significantly enriched in tumors with properties of Type II ovarian cancer; high grade, serous tumors, and poor survival. Conversely, the differentiated tumors share properties with Type I, including lower grade and mixed histological subtypes. The stem cell-like signature was prognostic within high-stage serous ovarian cancer, classifying a small subset of high-stage tumors with better prognosis, in the differentiated subtype. In multivariate models that adjusted for common clinical factors (including grade, stage, age), the subtype classification was still a significant predictor of relapse. The prognostic stem-like gene signature yields new insights into prognostic differences in ovarian cancer, provides a genomic context for defining Type I/II subtypes, and potential gene targets which following further validation may be valuable in the clinical management or treatment of ovarian cancer.

## Introduction

Ovarian cancer is the fifth most common cause of cancer deaths among women and is the leading cause of death from gynecological neoplastic disease [1]. The vast majority of initially responsive ovarian cancers eventually relapse [2], and this may be explained by a sub-population of stem cell-like chemotherapy-resistant tumors cells [3]–[5].

In breast cancer, there are widely-accepted molecular subtypes. Approximately 15% of breast cancers are estrogen receptor (ER)-negative, high-grade and often basal-like breast cancer that are enriched in cells expressing putative stem cell markers CD44^+^/CD24^−^ [6] and over-expresses genes associated with embryonic stem cell gene signatures [7]. This “stemness” may be explained, in part, by the observation that BRCA1, which is reported to regulate mammary stem cell fate [8], is often mutated in basal-like tumors [9].

In contrast, ovarian cancer has no consensus molecular subtype classification. Tothill et al. used k-means clustering of microarray data and described six molecular subtypes of serous and endometrioid ovarian cancer [10]. The Cancer Genome Atlas (TCGA) consortium identified four molecular subtypes of high grade serous ovarian cancers [11]. However, others have proposed pathology-site of origin based subtyping of ovarian cancer into Type I tumors, which are low-grade and histologically heterogeneous, and Type II tumors, which are high-grade and mostly serous [12], [13]. Type II is believed to arise largely in the fallopian tube epithelium while Type I’s site of origin is thought to be the ovary, although the cell of origin remains unclear [12], [13]. No Type I/II molecular signature exists, and classifying tumors as Type I or Type II based on clincopathologic analysis is generally but not always straightforward [14].

Here we report the identification of ovarian cancer subtypes based on the expression of genes associated with stem cell signatures. Using a computational approach, we demonstrate the presence of a poor prognosis, stem cell-like subtype in ovarian cancer that aligned closely with the cell of origin classification and provides the first genomic definition of Type I/II ovarian cancer. This gene expression profile does not demonstrate the existence of a subpopulation of cancer stem cells in these tumors. Instead it discovers common molecular pathways expressed by these cancers and stem cells. Tumors displaying expression of stem-like genes may have a less differentiated phenotype. Discovery of this stem cell subtype provides us a more complete understanding of ovarian cancer’s molecular diversity and opens up the potential for new and more directed approaches to treating and managing the disease.

## Methods

### Data

Stem-like cluster discovery was applied to ovarian cancer gene expression data published by Tothill et al. [10], as part of the Australian Ovarian Cancer Study (AOCS) data, and which were downloaded from Gene Expression Omnibus (GEO) [15] (GSE9891). AOCS samples (n = 206), were obtained from the Royal Brisbane Hospital (n = 22), Westmead Hospital (**n = **54), and Netherlands Cancer Institute (NKI-AVL; n = 3) [10], and gene expression was assayed on Affymetrix GeneChip U133 plus 2.0 arrays [10]. Raw data were normalized using RMA [16] with custom cell description files (CDFs) based on Ensembl gene mapping (version 12) as provided by the Microarray Lab at the University of Michigan [17]. Custom CDFs were used because updated probe set definitions provide better precision and accuracy compared to Affymetrix probe set definitions [18].

The four validation ovarian cancer gene expression datasets used in this analysis were from Dressman et al. [19]; Wu et al. (GSE6008) [20]; Tone et al. (GSE10961) [21]; and Crijns et al. [22]. The three validation breast cancer datasets were from Miller et al. (GSE3494) [23]; Desmedt et al. (GSE7390) [24]; and a merged dataset combining GSE2034 [25] and GSE5327 [26], which we called “Veridex.” All datasets were downloaded from GEO, except the Crijns dataset, which was received from the authors in normalized form as described in their paper [22], and the Dressman dataset was downloaded from the authors’ website [19]. TCGA ovarian cancer microarray dataset (n = 518) was downloaded from the TCGA data portal [11]. All datasets other than the Crijns, Wu, and Tone datasets were RMA-normalized with custom Ensembl CDF’s [17].

RMA-normalization was used in most datasets because of its highly reproducible results and correlation with RT-PCR data [27]. However, due to the inclusions of normal fallopian tube in the Tone dataset and normal ovarian surface epithelium samples in the Wu dataset, these datasets were normalized using the Invariant Set normalization method [28] to avoid the assumption within RMA of equivalent gene expression distribution. All validation data had been collected on Affymetrix GeneChip U133a arrays, except for the Tone and Crijns datasets, which had been collected on Affy U133 Plus 2.0 chips and Operon human v3 ∼35 K 70-mer two-color oligonucleotide microarrays, respectively.

In the Desmedt and Veridex breast cancer datasets, we predicted molecular subtypes as described by Desmedt et al. (2008) [29]. Specifically, subtypes based on gene markers ESR1, ERBB2, and AURKA were generated using the *subtype.cluster* function with model *scmgene* in Bioconductor package genefu. This entailed using mixture modeling to group patients into HER2+, ER−/HER2- (basal-like), or ER+/HER2- (luminal) [29]. While triple-negative breast cancer is not equivalent to basal-like breast cancer [30]–[32], most triple-negative breast cancer classify to the basal-like molecular subtype [32] leading us to regard prediction of ER−/HER2- an approximation of “basal-like”.

### Statistical Analysis

Statistical analyses, unless otherwise described, were performed using all available data and standard functions in R version 2.10.1. The ISIS algorithm [33] was applied to the AOCS dataset to generate unsupervised candidate bipartitions of the patients. For each candidate bipartition, ISIS calculates a diagonal linear discriminant (DLD) score with the most significant genes supporting the bipartition. Default parameters of the R implementation of ISIS were used, except that the number of genes used for scoring (“p”) was 100, a level which has been previously used [34]. We also took advantage of another paramater (“p.offs”) by ignoring the top 5 most related genes in order to reduce the effects of high leverage genes on scoring, resulting in a 95-gene signature used for scoring each bipartition.

To diminish confounding effects from differential stroma and non-tumor cells in different arrayed sites (e.g. peritoneal or ovary) (unpublished data), only AOCS arrays of ovarian mRNA from malignant, serous, and primary site ovarian tumors from patients that did not receive neoadjuvant therapy were included, reducing the dataset from 285 to 145 patients. Analyses of the AOCS dataset were performed on this subset unless we specify the “entire” AOCS dataset (n = 285) or “remaining” AOCS data (n = 140), which were tumors not used for ISIS class discovery.

Genes used for subtype cluster discovery were limited to 83 mouse and human gene signatures of adult, cancer, or embryonic stem cells obtained from GeneSigDB [35] that had at least 5 and at most 1,000 genes. For each gene signature, we retrieved the article describing the gene signature to confirm its description and association with adult, cancer, or embryonic stem cells. Stem-like gene signatures frequently contain proliferation genes [7]. To avoid dividing patients based on proliferation, we also removed genes (n = 580, Table S1 in Methods S1) associated with proliferation (see Methods S1), similar to analyses by Ben-Porath we al. [7].

The resulting matrix of 2,632 stem-like genes was subject to ISIS bipartition discovery. The highest scoring bipartition that was significantly (p<0.05) associated with grade and disease-free survival was selected for further investigation. These criteria were based on the finding that the stem cell-like sub-population of breast cancer tumors discovered by Ben-Porath et al. was characterized by high grade and poor prognosis [7]. The association with prognosis was secondary since the Ben-Porath et al. analysis primarily described association between stem cell-like gene expression and higher grade [7]. We selected the bipartition that most closely satisfied the criteria and called this bipartition the “stemness bipartition”. Leave-one-out cross-validation was used to refine the gene list defining the stemness bipartition, and the genes which were in each 95-gene list in all 145 folds formed an ovarian cancer “51-gene stemness signature” (Table S2 in Methods S1).

Gene-Set-Enrichment Analysis (GSEA) with nonparametric inference for linear models as implemented in *gsealmPerm* in the package gsealm in Bioconductor [36] was performed with curated gene sets (C2) and Gene Ontology (GO) gene sets (C5) from the gene set database MSigDB version 3.0 [37]. GSEA was also performed using the 13 stem cell gene sets of Ben-Porath et al.: two describing embryonic stem cells; four activated by Nanog, Oct4, and/or Sox2; four bound by the Polycomb repressive complex 2 PRC2; and two activated by c-Myc [7].

### Class Prediction

To confirm the presence of the stem-like subtype in ovarian cancer, the stem-like subtype classification was applied to multiple independent microarray datasets. In order to predict the class of new tumors, we first needed generate a “stemness molecular classifier,” a model of gene weight which discriminated the stem-like and differentiated tumors. Diagonal linear discriminant (DLD) analysis [38] was used to train this gene classifier using expression profiles of the 51 genes in the AOCS dataset (Table S2 in Methods S1). To predict the subtype of new tumors, expression profiles were projected as supplementary points onto the DLD axis, and the DLD score was the weighted sum of the expression of the genes. As the DLD scores of new ovarian tumors projected on this axis were bimodally distributed (Figure S1 in Methods S1), Gaussian mixture modeling [39] was used to define the two populations and assign new tumors to either the stem-like or differentiated subtype. The DLD score is the linear combination of weighted expression of many genes, and although we did not investigate the effect of batch-specific technical variation [40] in depth, we found it robust to mild noise from missing data and outliers in the datasets tested. This stemness molecular classifier was applied to each validation dataset.

The ovarian cancer datasets used for validation are phenotypically and clinically heterogeneous, and contain different histological subtypes, grades, prognoses, treatments and follow-up protocols. Unless stated we did not control for phenotype variation; instead we exploited the heterogeneity in datasets, in particular histology and grade, to explore how the bipartition associated with phenotypes beyond those represented in the AOCS dataset and to determine the extent to which the stemness molecular classifier could be generalized.

## Results

### Discovery of a Stem Cell-like Subtype in Ovarian Cancer

To explore whether ovarian cancer has a stem-like component, we tested whether genes reported to be expressed by stem cells are also expressed in a subset of ovarian tumors. To do this, we extracted the union of all adult, cancer, or embryonic stem (ES) cell gene expression signatures in GeneSigDB [35] as described in Methods S1 to generate a list of 2,632 stem-like genes (Table S3 in Methods S1).

We then took the AOCS ovarian cancer gene expression data (n = 145 patients) and considered only the 2,632 genes reported to be expressed in stem cells. To this, we applied ISIS [33], an unsupervised bipartition clustering algorithm that randomly partitions samples into two subsets and selects the genes that most significantly associate with the partition. ISIS identified twenty-eight separate distinct, statistically significant patient bipartitions of the data, and each of which was further tested for association with grade and disease-free survival.

**Figure 1:**
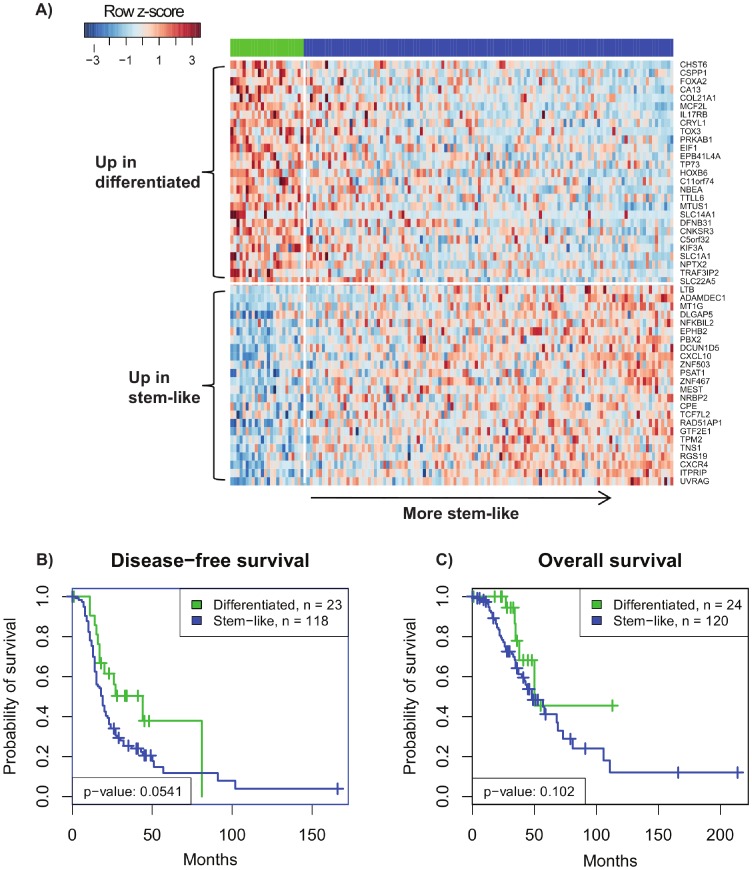
Heatmap of gene expression and Kaplan-Meier survival curves for the stemness bipartition. (A) A heatmap of gene expression profiles of the 24 differentiated (green) and 121 stem-like (blue) tumors from the AOCS dataset [10]. The tumors are ordered by increasing stemness molecular subtype score, and the 51 classifier genes are ordered from top to bottom by increasing over-expression in the stem-like subtype according to a pooled *t*-test. The Kaplan-Meier curves are with respect to (B) disease-free survival and (C) overall survival and are not significant at p<0.05, but this is possibly due to the small size of the differentiated subtype.

The top scoring bipartition, hereafter referred to as the ovarian cancer stemness bipartition differentiated two distinct subgroups of ovarian cancer patients: a set of 121 patients with worse disease free (p = 0.0541), overall survival (p = 0.102), and higher grade (p = 0.00326) that was interpreted as more “stem cell-like” as these tumors over-expressed a number of genes known to be associated with stemness, and a smaller group of 24 patients with better survival and lower grade that we refer to as the “differentiated” subgroup (Figure 1).

Leave-one-patient-out cross-validation was performed to extract the most robust gene signature of this bipartition, resulting in a 51-gene stemness signature (Table S2 in Methods S1). Although the bipartition’s association with overall survival showed a trend and did not reach conventional statistical significance, the signature was significantly prognostic in subsequent analyses when the sample size was larger (see below).

To provide further support for the phenotypes revealed by the bipartition, we tested if gene targets known to be expressed in stem cells were differentially regulated between the stem-like and differentiated subtypes using gene set enrichment analysis (GSEA). Thirteen lists of genes (Table S4 in Methods S1) which have been previously used to characterize stem cells [7] but were not among the initial 83 signatures used to discover the stem-like ovarian subtype were examined. Activation targets of Nanog, Oct4, Sox2 and c-Myc, which are up-regulated in stem cells, were also up-regulated in the stem-like subtype, and eight of nine of these gene lists were significantly (p<0.05) different across the subtypes (Table 1). Four sets of Polycomb-regulated genes, which characterize more differentiated cells, were up-regulated in the differentiated subtype when compared to the stem-like subtype and were close to significance (p<0.10). Since some of these gene sets are reported to be dependent on proliferation genes [7], we also performed a modified gene set analysis with proliferation genes excluded as previously described [7]. Even with this modification, the stem-like subtype was enriched in gene expression of ES [41] (p<0.0001), Nanog [42] (p<0.05), and c-Myc [43] (p<0.05) targets. High expression of the same ES gene set is reported in high-grade, estrogen receptor (ER)-negative breast tumors [7].

**Table 1:** The stem-like and differentiated subtypes are enriched in stem cell and differentiated gene sets respectively.

	Gene Set	Enrichment	P-value	Adjusted P-value*
**Embryonic stem cell genes**	ES exp1	Stem-like	0.00002	0.00004
	ES exp2	Stem-like	0.00031	0.01671
**NOS targets**	Nanog targets	Stem-like	0.00115	0.01573
	Oct4 targets	Stem-like	0.01509	0.07951
	Sox2 targets	Stem-like	0.02296	0.12607
	NOS targets	Stem-like	0.00969	0.10489
	NOS TFs	Stem-like	0.09596	0.13822
**Myc targets**	Myc targets1	Stem-like	0.01144	0.03774
	Myc targets2	Stem-like	0.01349	0.10387
**Polycomb targets**	Suz12 targets	Differentiated	0.05251	0.05508
	Eed targets	Differentiated	0.06293	0.05605
	H3K27 bound	Differentiated	0.05046	0.06329
	PRC2 targets	Differentiated	0.08553	0.09058

* Analysis was repeated after removing proliferation-related genes from the gene sets, as described by Ben-Porath et al. [7]. No multiple testing correction was performed.

### Identification of Functional Links to Basal-like Breast Cancer

To further characterize tumors in the stem-like subtype, we performed GSEA using all of the gene sets in MSigDB [37] to identify which gene sets were enriched in genes differentially expressed between tumors in the stem-like and differentiated subtypes (again, these sets did not include the gene sets initially utilized to discover the bipartition). Genes over-expressed by stem-like tumors were especially enriched (p<0.0001) for gene sets describing poor prognosis and undifferentiated cancers; high-grade, invasive ovarian cancer; Myc targets; and embryonic stem cells, BRCA1 mutation, estrogen receptor (ER)-negative status, and the basal-like subtype in breast cancer (Table S5). In contrast, gene sets strongly enriched in the differentiated subtype included those related to cellular projections (Table S6), ER-positive breast cancer, and low malignant potential (LMP) and low-grade ovarian cancer (Table S5 in Methods S1).

Both high-grade, serous ovarian and basal-like breast cancer are seen in women with mutant BRCA1 [9], [44]. To investigate the GSEA prediction that the stem-like serous ovarian cancer molecular subtype is enriched for genes also expressed in high grade basal-like breast cancer, we applied diagonal linear discriminant analysis [38] to the AOCS ovarian cancer data to train a stemness molecular classifier and predicted the “stem-like” or “differentiated” classification of tumors in two published breast gene expression datasets (Desmedt [24] and Veridex [25], [26] datasets). The gene list we applied to the breast cancer datasets was not optimized for breast cancer, and the DLD scores did not exhibit bimodal distributions. However to maintain consistency with the methodology applied to ovarian cancer data, we used the same Gaussian mixture modeling approach to dichotomize the DLD scores of breast cancers defining them as stem-like or differentiated. The resulting stemness classification of breast tumors confirmed the gene set analysis predictions; breast tumors assigned to the stem-like subtype were significantly enriched in basal-like molecular subtype and high-grade (logistic regression likelihood ratio test p = 1.42×10^−9^ and p = 0.00964, Desmedt and Veridex datasets respectively – Table 2) breast cancer.

**Table 2:** The stem-like subtype is significantly overrepresented in basal-like breast cancer.

	Desmedt***		Veridex***	
	Basal	Non-basal	Basal	Non-basal
Stem-like	35	14	90	63
Differentiated	11	138	12	179

*** Fisher’s exact test p = 3.50×10^−18^ and p = 1.11×10^−27^, Desmedt and Veridex datasets respectively.

### Reproducibility of the “Stemness” Phenotypes in Independent Datasets

To validate our ovarian stem-like and differentiated molecular subtypes, we applied the stemness DLD molecular classifier to three independent ovarian cancer microarray datasets and the “remaining AOCS data” (n = 140) not used in the initial bipartition discovery. Two datasets, Crijns et al. [22] and Dressman et al. [19], consisted of high-stage, serous tumors, while the other two, the Wu et al. dataset [20] and the remaining AOCS data [10] were histologically heterogeneous.

First, we confirmed the association between grade and the stem-like molecular subtype. In these independent data, stem-like tumors had higher grade in the Wu (n = 103, logistic regression p = 1.63×10^−5^), remaining AOCS (n = 140, p = 1.16×10^−7^), and Dressman (n = 118, p = 0.073) datasets.

Next we explored which histological subtypes of ovarian cancer were classified as stem-like. Serous is the most common histological form of epithelial ovarian tumor, but epithelial ovarian cancer is a heterogeneous disease with mixed malignancy potentials and histological subtypes, including endometrioid, clear cell, and mucinous [12], [13]. Despite initial classification being performed only on malignant, serous tumors, the stemness molecular classifier discriminated between different histological subtypes. In the Wu dataset [20], most serous tumors (29/41) were “stem-like”, but most endometrioid (22/37), almost all clear cell (7/8), and all mucinous (13/13) tumors, as well as all four (4/4) “normal” ovarian surface epithelium samples were “differentiated.”

To further evaluate association with histological subtype, we examined the entire AOCS dataset (n = 285). This larger dataset was comprised of the serous AOCS discovery dataset (n = 145) and the remaining AOCS data (n = 140), which included LMP serous tumors and malignant endometrioid and serous tumors arrayed from sites other than the ovary. We observed that the differentiated subtype was significantly enriched in endometrioid tumors (9/20, Fisher’s test p<0.05 after FWER correction [45]). Of note, the stemness DLD scores were significantly lower in the LMP serous tumors (Wilcoxon rank-sum test p-value = 1.09×10^−4^) than in the malignant, differentiated tumors, and almost all (17/18) LMP tumors were classified as differentiated. Additionally, within the stem-like subtype the DLD score was significantly associated with higher grade (p = 0.00159), though it was not correlated (p>0.05 after FWER correction) with stage, overall survival, or disease-free survival, suggesting that further investigation into the clinical value of the continuous DLD score within subtypes is warranted.

The stem-like subtype classification was not equivalent to the classification recently proposed by Tothill et al. in which serous and endometrioid tumors are identified as one of six molecular subtypes, C1–C6 [10]. The stem-like tumors (n = 233) were not classified into a single molecular subtype but instead were mostly distributed among poor prognosis subtypes C1 (n = 80), C2 (n = 48), C4 (n = 39), C5 (n = 29) and Not Classified (n = 30). A considerable number of differentiated tumors were in good prognosis molecular subtypes C3 (n = 25) and C6 (n = 4), and the remaining 23 differentiated tumors were distributed among the other subtypes (Table 3). So, while the bipartition roughly separated better prognosis low-grade subtypes C3 and C6 from the others and overlap with the molecular subtype classification was significant, no combination of AOCS subtypes fully explained the bipartition. However, it should be noted that the C1–C6 subtype classification was arrived at using *k*-means clustering [10], a method that is not deterministic in the sense that re-running the algorithm can produce different clusters, so the assignments by the AOCS should be considered only approximate.Prognostic value of the stem-like phenotype.

**Table 3:** Clinical characteristics of patients in stem-like and differentiated subtypes.

Dataset			Differentiated	Stem-like	P-value
**Wu et al**	Stage	I	31	4	***
**(n = 103)**		II	6	5	
		III	13	31	
		IV	4	5	
	Grade	1	18	1	***
		2	9	8	
		3	14	24	
	Histology	Clear Cell	7	1	***
		Endometrioid	22	15	
		Mucinous	13	0	
		OSE	4	0	
		Serous	12	29	
**Entire AOCS**	Type	LMP	17	1	***
**(n = 285)**		Malignant	35	232	***
	Stage	I	15	9	
		II	8	10	
		III	28	189	
		IV	1	21	
	Grade	1	14	5	***
		2	19	78	
		3	17	147	
	Histology	Adenocarcinoma	0	1	*
		Endometrioid	9	11	
		Serous	43	221	
	Primary site	Fallopian tube	0	8	**
		Ovary	52	191	
		Peritoneum	0	34	
	Arrayed Site	Other	0	14	***
		Ovary	50	150	
		Peritoneum	2	69	
	Age	Median age	56.3	59.3	*
	Residual disease	<1 cm	43	118	**
		>1 cm	5	76	
	Molecular subtype	C1	3	80	***
		C2	2	48	
		C3	25	3	
		C4	7	39	
		C5	7	29	
		C6	4	4	
		NC	4	30	

** p-value <0.01,

*** p-value <0.001. OSE Ovarian surface epithelium, NC not classified. In each dataset, p-values were corrected for family-wise error rate using Hommel’s method [46], except for the test for association with the Tothill et al. molecular subtypes.

**Figure 2:**
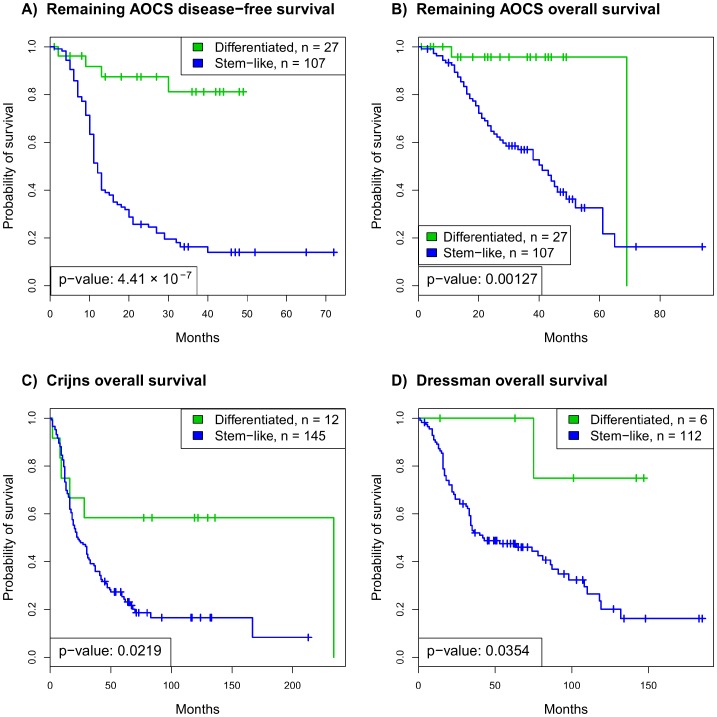
Validation of the stemness bipartition in independent ovarian cancer microarray datasets, as well as in the remaining AOCS dataset. In the remaining AOCS dataset, the stem-like subtype has strongly worse (A) disease-free survival (p<0.001) and (B) overall survival (p = 0.00127). In the (C) Crijns and (D) Dressman datasets, the stem-like subtype has significantly worse overall survival (p = 0.022 and p = 0.035, respectively).

In the three independent validation datasets and the remaining AOCS data, tumors with the stem-like subtype had worse prognosis (Figure 2). The stem-like subtype had both significantly worse overall survival (log-rank test p = 4.41×10^−7^) and disease-free survival (p = 0.00127) in the remaining AOCS dataset (n = 140) and worse overall survival in the Crijns (n = 157, p = 0.021) and Dressman datasets (n = 118, p = 0.0354). Although the Wu dataset [20] did not include survival information, the stem-like subtype’s tumors had significantly higher stage (n = 103, p = 1.03×10^−6^), which suggests worse prognosis.

A finding of potential clinical importance is that the stemness molecular classifier may also be prognostic within high-grade, high-stage serous ovarian cancer. The stem-like subtype had worse disease-free (p = 0.0053) and overall survival (p = 0.0299) in high-grade, malignant tumors of the entire AOCS dataset. In independent analysis of each histology, the stem-like subtype was associated with poorer disease-free survival in high-grade serous (p = 0.0447), but was not a significant predictor in high-grade endometrioid tumors (p = 0.278). In the Crijns and Dressman datasets, which were exclusively high-stage serous tumors, the stemness molecular classifier identified a small subset of differentiated subtype tumors with better overall survival (Figure 2). Equally, across datasets the differentiated subtype included a small number of high-grade tumors (grades 2 or 3) [14] with better prognosis. Further analysis is needed to determine if this classification is useful in identifying high-grade tumors more likely to have a favorable outcome.

The stem-like subtype was associated with phenotypes often predictive of poor outcome (Table 3), but the stem-like subtype’s prognostic ability is not fully explained by these common clinical variables. In the entire AOCS dataset the stem-like subtype was a strong predictor of outcome (univariate analysis DFS p = 3.23×10^−6^, OS p = 0.00122), was associated with greater patient age (p<0.05), and was strongly (p<0.001 after FWER correction [46]) associated with high stage; peritoneal arrayed site, a site to which high-grade ovarian cancer frequently spreads; and greater residual disease after surgery.

Despite these associations, in multivariate analysis, the stemness bipartition remained a strong predictor of worse disease-free survival. The bipartition remained a significant predictor of disease-free survival when adjusting for one (p<0.005) or two variables among stage, grade, and residual disease (Table 4) or any combination of two variables (p<0.05) in Table 3, with the exception of adjusting for both stage and low malignancy potential (p = 0.0785). Notably, the stem-like subtype had significantly worse disease-free survival (p = 0.0143) in multivariate analysis adjusting for grade, histological subtype, and low malignancy potential. Even when adjusting for stage, low malignancy potential, histological subtype, and grade, the stem-like subtype still had a 54% increased odds of relapse, although this was not significant (p = 0.126).

**Table 4:** Multivariate Cox proportional hazards models of the stemness bipartition to predict relapse-free survival when adjusting for two prognostic variables (A) residual disease and stage, (B) grade and stage or (C) grade and residual disease.

Variable	Hazard ratio	Lower limit (95% CI)	Upper limit (95% CI)	P-value
**(A) Adjusting for residual disease and stage**				
Stem-like subtype	1.75	1.00	3.05	0.0498*
Residual disease	1.43	1.02	2.00	0.0374*
Stage^1^	7.30	2.57	20.8	0.000195***
**(B) Adjusting for grade and stage**				
Stem-like subtype	2.17	1.22	3.85	0.00818**
Grade^1^	1.16	0.586	2.29	0.674
Stage^1^	7.72	2.80	21.2	7.64×10^−5^ ***
**(C) Adjusting for grade and residual disease**				
Stem-like subtype	2.36	1.32	4.22	0.00370**
Grade^1^	1.42	0.672	3.01	0.358
Residual disease	1.76	1.26	2.46	0.000872***

* p<0.05,

** p<0.01,

*** p<0.001.

1 In regression analyses, the ordinal variables stage and grade were broken into multiple components using default functions in R. However, only the linear components (levels treated as a continuous variable) are displayed in the table because the other components were not significant. Grade was also coded as a quadratic component (grade 2 vs. grades 1 and 3) and stage as both quadratic (stages 2 and 3 vs. stages 1 and 4) and cubic (stage 2> stage 4> stage 1> stage 3) components.

### Stem-like Tumors have Characteristics of Type II Ovarian Cancer

A recent pathogenesis model of ovarian cancer divides tumors into Type I, which is low-grade and histologically diverse, and Type II, which is high-grade and mostly serous [12], [13]. A gene signature-based molecular classification for Type I/II ovarian cancer has not yet been described, and Type I/II are distinguished largely based on their morphological properties (Table 5). In comparing our molecular subtypes to these morphological classifications, we found stem-like tumors to possess characteristics of Type II ovarian tumors and the differentiated tumors to be similar to Type I (Table 5).

**Table 5:** Shared characteristics of Types I and II ovarian cancer and the stem-like and differentiated subtypes [12].

	Type I	Type II	Stemness bipartition
**Presence of ciliated cells**	Possibly	No	Differentiated subtype is enriched in genes related to cilia and more similar to normal fallopian tube.
**Histological subtypes**	Serous, endometrioid, mucinous,clear cell	Mostly serous	Stem-like subtype overrepresented serous ovarian cancer while the differentiated subtype had mixed histology (Table 3).
**Mutations**	KRAS, BRAF PTEN, CTNNB1, ERBB2, PIK3CA	Mostly p53	Stem-like subtype is enriched in p53-mutant tumors and p53 mutation-associated genes. Differentiated subtype is enriched in other mutations.
**Benign tumors**	Sometimes	No	Almost all 18 of the LMP tumors were classified as differentiated in entire AOCS dataset.
**Grade**	Low grade	High grade	Stem-like subtype has higher grade across datasets.
**% epithelial ovarian carcinomas**	25%	75%	Original stem-like subtype comprises 83% of the tumors and in entire AOCS dataset, 77% of tumors.
**% ovarian cancer deaths**	10%	90%	Stem-like consists of 91% of deaths in original bipartition and 94% in entire AOCS dataset.

Both Type II and the stem-like subtype are associated with poor prognosis, high-grade serous tumors (Table 5). Although formal evaluation of the stem-like and differentiated subtypes’ prevalence and lethality would require prospective random sampling of ovarian carcinomas and subsequent classification, the stem-like subtype properties would appear to be consistent with the reports Type II’s relative prevalence and lethality (Table 5).

Mutations characteristic of Type II ovarian cancer are found in the stem-like subtype. Type II tumors are thought to arise from precursor lesions in fallopian tube epithelium and have “p53 signatures” that have strong p53 immunoreactivity and usually p53 mutations [13]. It is reported that most Type II ovarian tumors (>80%) have p53 mutation [12]. TCGA ovarian cancer data consists of 489 high-grade serous ovarian adenocarcinomas and almost all have TP53 mutation (96%) [11]. In our analysis stemness molecular subtype scores of TCGA ovarian cancers lacked bimodality and most tumors were classified into one subtype which was more stem-like (data not shown). The mutation status of almost all samples in other ovarian microarray datasets are unknown but Wu et al. [20] reported p53 mutation status of endometrioid tumors (n = 37), and these endometrioid tumors with p53 mutations were overrepresented in the stem-like subtype (p = 0.016). In addition, we observed that genes expressed by the stem-like ovarian cancer subtype are enriched for two gene sets that are over-expressed by p53-mutant breast cancers relative to breast cancer without p53 mutation [47], [48] (GSEA p-values 0.00008 and 0.00004, respectively in AOCS dataset–Table S7 in Methods S1). To confirm this association, we applied the stemness bipartition classification to a breast cancer dataset in which the p53 status of tumors is known [23], and the stem-like subtype had strong overlap with p53-mutant tumors (Fisher’s exact test p = 1.36×10^−12^).

### Differentiated Tumors have Characteristics of Type I Ovarian Cancer

In contrast, “differentiated” tumors and Type I tumors describe histologically diverse and mostly (although not exclusively) low-grade and LMP tumors (Table 5). Type I tumors are characterized by other mutations (including KRAS, BRAF, PTEN, and PIK3CA and others shown in Table 5) [12], and we observed that all 15 endometrioid tumors in the Wu dataset with CTNNB1, PTEN, or PIK3CA mutations were characterized as differentiated. Moreover, all 13 tumors in this dataset with β-catenin accumulation were differentiated, which is consistent with aberrations in Wnt signaling seen in low-grade endometrioid tumors [12]. Thus, the subtype classification was consistent with reported mutation status of Type I/II tumors.

Type I tumors purportedly arise from either ovarian surface epithelium that undergoes metaplasia or epithelium of fallopian tube, endometrium, or peritoneum that proliferates after being trapped in ovarian cortical inclusion cysts [13] with the cell of origin possibly being normal fallopian tube epithelium (FTE) that sheds by endosalpingiosis [12], [13]. We tested whether the differentiated subtype’s gene expression patterns are similar to that of normal FTE by applying our stemness classification to the Tone et al. dataset [21] in which the authors compared expression profiles of serous ovarian cancer and normal FTE from women with and without BRCA1/2 mutation. Our stemness classification assigned 13/13 ovarian cancer samples to the stem-like group and 24/24 FTE samples to the differentiated. Although all four normal OSE samples in the Wu dataset [20] were also classified as “differentiated,” these results suggest substantial similarity in gene expression profiles between FTE and the differentiated subtype.

Consistent with the hypothesis that Type I (but not Type II) tumors are enriched for expression of genes associated with cilia [12], the differentiated subtype of the AOCS dataset (n = 145) was enriched in gene sets associated with cilia, such as apical projections gene sets (Table S6 in Methods S1) and FOXJ1, which is required for ciliogenesis [49] and has been proposed as a marker for ciliated fallopian tube cells [50]. FOXJ1 is not in the 51-gene stemness signature but was strongly up-regulated in the differentiated subtype compared to the stem-like subtype (*t*-test p = 4.87×10^−7^). These observations provide evidence for the hypothesis that Type II/high-grade serous ovarian cancer arises from non-ciliated epithelial cells [13].

Therefore, the Type I/II and stemness classifications are similar in terms of grade, histological subtype, prevalence, and lethality, in addition to cell of origin, presence of ciliated cells, and mutation status (Table 5). From a gene expression point of view, Type II ovarian tumors may be more stem-like in their gene expression. If this molecular definition holds in further prospective studies, our stem-like classification would represent a molecular classification system that could establish the Type I/II system and provide insight into possible mechanism and therapies for these subtypes.

### Biological Basis of the Stem-like Gene Classifier

Of the 51 genes used for classification, 37 were present in all four ovarian cancer datasets used for clinical validation (remaining AOCS, Wu, Dressman, and Crijns), because the gene expression profiling was performed on different technological platforms. Of these, a subset of 12 were consistently over-expressed (p<0.05 after FDR correction) in either the stem-like or differentiated subtypes across the four datasets and are thus most robustly expressed. The six stem-like subtype genes were UVRAG, CXCR4, RGS19, RAD51AP1, PSAT1, and CXCL10, and the six differentiated subtype genes were FOXA2, EIF1, MTUS1, DFNB31, TRAF3IP2, and SLC22A5. Despite enrichment in gene expression of the targets of Nanog, Oct4, and Sox2 in the stem-like subtype (Table 1), we did not find that that these genes were differentially expressed between the stem-like and differentiated subtypes.

## Discussion

The cancer stem cell theory proposes that a subpopulation of cells inherit or acquire stem-like properties that enable them to survive therapy and drive recurrent tumor growth, but the function and identification of such stem cells is controversial both in normal and malignant ovarian and fallopian tube tissue [51]–[53]. We have not demonstrated the existence of cancer stem cells in ovarian cancer; instead our analysis identified a subtype of ovarian cancer with stem-like gene expression which provides a new molecular subtype classification of ovarian cancer, a genomic context for further investigation of type I/II ovarian cancers and insights into why ovarian cancer is so likely to be fatal despite aggressive therapy.

Tumors identified as being of the stem-like subtype have higher tumor grade and significantly worse prognosis, properties that were reproducible in independent and heterogeneous ovarian cancer microarray datasets; the associations between stem cell-like gene expression and grade or survival have been observed before but has not been explored in ovarian cancer [7], [54]. In our analysis, stemness was also a significant predictor of disease-free survival in multivariate analyses that adjusted for prognostic variables, such as grade and stage. Thus, this classification’s prognostic value may be independent of confounding clinical variables.

The classification is also valuable because of similarity to and support for a subtype classification associated with distinct pathogenesis pathways. Type I ovarian cancer, which includes low-grade and histologically heterogeneous tumors that may arise in the ovary, is similar to the differentiated subtype while Type II, which includes high-grade and mostly serous tumors that arise in the fallopian tube, is similar to the stem-like subtype [12], [13]. This is the first potential gene expression-based description of Type I and II ovarian tumors (a distinction that up to now was mainly morphologic), and thus it provides rationale for new biologic and treatment hypotheses.

Our signature classified a low number of high-grade, serous tumors, which would normally be classified as Type II, as Type I. The identification of good prognosis, high-grade serous carcinomas may reflect novel biological insight or gene expression patterns of tumors that were originally low-grade and became high-grade [55]. Alternatively, these tumors may reflect initial misclassification in tumor grade because such clinical pathological classification of Type I and II tumors is mostly not always straightforward [14].

The 51 classification genes we identified may provide insight into pathogenesis of Type I and II ovarian cancer. FOXA2, which is consistently up in the differentiated subtype, is an inhibitor of the epithelial-mesenchymal transition associated with invasion and metastasis [56], [57]. On the other hand, CXCR4, which is consistently highly expressed in the stem-like subtype, is implicated in ovarian cancer metastasis [58], and is a potential therapeutic target of drugs such as CXCR4 antagonists AMD3100 [58] and CTCE-9908 [59]. Other genes including UVRAG and RAD51AP1 are implicated in DNA damage response, which has been recently linked to cell differentiation status [60].

The stem-like subtype was enriched in stem cell-related gene sets, including gene targets of stem cell markers Oct4 and Nanog, which are reported to be associated with grade and stage in serous ovarian cancer [61], [62]. Although many common markers of stem cells or EMT were not up-regulated in stem-like ovarian cancer, expression of stem cell markers were also not observed in the stem cell-like high-grade, ER-negative breast cancer [7]. This may be because stem cell markers are up-regulated in a minority of cancer stem cells which would not be reflected in gene expression profiling of whole tumor. Kim et al. proposed that Myc activity explains the apparent contradiction between the predictive power of stem cell-related transcriptional programs and the absence of canonical stem cell gene expression [61], [62]. In our analysis, we observed an enrichment of the Kim et al. Myc module in the stem-like subtype, but without an over-expression of Myc (AOCS dataset *t*-test p = 0.445).

The stem-like subtype was predominant in high grade serous ovarian and basal-like breast cancer supporting common biological connections between these cancers that also share BRCA1 dysfunction [9], [44] and p53 mutation [63].Molecular similarities between basal-like breast cancer and high-grade serous ovarian cancer have been described in other studies, including the recent study by the Cancer Genome Atlas Network [64]. Inactivation of p53 in breast cancers has been shown to correlate with stem cell transcriptional signatures [65] and the basal-like subtype [9] in breast cancer. The guardian of the genome, p53 maintains DNA integrity and DNA repair processes but also plays an important role in stem cell and cell state reprogramming. While the majority of ovarian cancer patients (∼ 80%) have tumors of the stem-like subtype, the stem-like is observed in only 15% of breast cancers which may partially explain the relatively poor prognosis in ovarian cancer [1]. Our analysis provides additional support that therapies effective in basal-like breast may also be may also be effective in high grade serous ovarian cancer. Poly(ADP-ribose) polymerase (PARP) inhibitors, which are especially effective in cancer associated with BRCA1 or BRCA2 mutation [66], have shown considerable promise in triple-negative breast cancer [67] and may be valuable in treating high-grade serous Type II ovarian cancer [68].

We present a prognostic stem-like subtype classification of ovarian cancer which provides a genomic context for the Type I/II classification. Though it does not explain all variation in survival, in combination with other clinical indicators, it may have the potential to explain prognosis with greater accuracy than currently used clinical variables can alone. This classification requires further experimental validation in a large cohort of patients to characterize the properties of each molecular subtype, their association with Types I and II ovarian cancer and demonstrate possible clinical application. Our study provides support for the recently described Type I/II model of ovarian cancer and provides a molecular signature for stratification of these subtypes.

## Supporting Information

Methods S1 This file contains: Figure S1 Distributions of stemness bipartition diagonal linear discriminant (DLD) scores in each dataset. In the ovarian cancer datasets, the DLD scores were all bimodal, and Gaussian mixture modeling [39] of the score was used to classify the lower scoring group as differentiated and the higher scoring group as stem-like. In the breast cancer datasets, mixture modeling was still used to discover approximate stem-like and differentiated classes, despite lack of clear bimodality. The range of scores is inconsistent in different datasets, because genes were often lost when crossing microarray platforms and potentially because of batch effects across datasets. Table S1 Proliferation genes (see Supplementary Methods) that were removed from the set of 83 GeneSigDB [35] gene signatures that were used for class discovery with ISIS [33]. After removing these genes, 2,632 genes were still used for class discovery.Table S2 Genes used to generate the stemness bipartition and their corresponding weights when predicting the bipartition in other datasets, as well as pooled *t*-statistics for differential expression between the stem-like and differentiated subtypes. Negative weights correspond to the differentiated subtype and positive weights correspond to the stem-like subtype. Table S3. A list of the 83 gene signatures from GeneSigDB [35] that are reported to be associated with stem cells. Table S4 The 13 gene sets used by Ben-Porath et al. to characterize classes of cancer as stem cell-like or differentiated. Genes in these gene sets were limited to those that were in the DNA microarray platform (Affy U133 Plus 2.0 platform) of the AOCS data [10]. Table S5 Gene-Set-Enrichment p-values for enrichment in stem-like and differentiated subtypes for all of the curated gene sets on MSigDB v 3.0. No multiple testing correction was used. Table S6 Gene-Set-Enrichment p-values for enrichment in stem-like and differentiated subtypes for all of the Gene Ontology gene sets on MSigDB v 3.0. No multiple testing correction was used. Table S7 Genes over-expressed in breast cancer cells containing wild type and mutant p53 from Figure 5 of Troester et al. [47] and Table 1 of Takahashi et al. [48] and whether they are over-expressed in the stem-like or differentiated subtype. P-value is for over-expression in either the stem-like or differentiated class and was calculated with the likelihood ratio test for logistic regression. Only genes in the AOCS dataset were considered. No multiple testing correction was performed. In the Troester et al., the Gene-Set-Enrichment p-value for enrichment of mutant p53 genes in stem-like subtype was 0.00004 and p-value for enrichment of WT p53 genes in differentiated subtype was 0.00005. These p-values were 0.00008 and 0.05144, respectively, in the Takahashi et al. signature.
(DOCX)
