# Supplementary material for: Stem Cell-Like Gene Expression in Ovarian Cancer Predicts Type II Subtype and Prognosis
Source: PLoS One. 2013 Mar 11;8(3):e57799. doi: 10.1371/journal.pone.0057799 (PMC3594231; doi:10.1371/journal.pone.0057799)
Supplement: Methods S1 — This file contains: Figure S1 Distributions of stemness bipartition diagonal linear discriminant (DLD) scores in each dataset. In the ovarian cancer datasets, the DLD scores were all bimodal, and Gaussian mixture modeling [39] of the score was used to classify the lower scoring group as differentiated and the higher scoring group as stem-like. In the breast cancer datasets, mixture modeling was still used to discover approximate stem-like and differentiated classes, despite lack of clear bimodality. The range of scores is inconsistent in different datasets, because genes were often lost when crossing microarray platforms and potentially because of batch effects across datasets. Table S1 Proliferation genes (see Supplementary Methods) that were removed from the set of 83 GeneSigDB [35] gene signatures that were used for class discovery with ISIS [33]. After removing these genes, 2,632 genes were still used for class discovery.Table S2 Genes used to generate the stemness bipartition and their corresponding weights when predicting the bipartition in other datasets, as well as pooled t-statistics for differential expression between the stem-like and differentiated subtypes. Negative weights correspond to the differentiated subtype and positive weights correspond to the stem-like subtype. Table S3. A list of the 83 gene signatures from GeneSigDB [35] that are reported to be associated with stem cells. Table S4 The 13 gene sets used by Ben-Porath et al. to characterize classes of cancer as stem cell-like or differentiated. Genes in these gene sets were limited to those that were in the DNA microarray platform (Affy U133 Plus 2.0 platform) of the AOCS data [10]. Table S5 Gene-Set-Enrichment p-values for enrichment in stem-like and differentiated subtypes for all of the curated gene sets on MSigDB v 3.0. No multiple testing correction was used. Table S6 Gene-Set-Enrichment p-values for enrichment in stem-like and differentiated subtypes for all of the Gene Ontology gene set [file pone.0057799.s001.docx]

**Supporting Information: Materials &Methods**

### Stem cell-like gene expression in ovarian cancer is prognostic and associated with Type II tumor characteristics

Matthew Schwede^a^, Dimitrios Spentzos^c^, Stefan Bentink^a,d^, Oliver Hofmann^b^, Benjamin Haibe-Kains^a^, David Harrington^a,b^, John Quackenbush^a,b,e^, Aedín C. Culhane^a,b^

Table of Contents

[Software 2](#_Toc300574496)

[Data pre-processing 2](#_Toc300574497)

[AOCS dataset clustering 3](#_Toc300574498)

[Statistical analysis 5](#_Toc300574499)

[Gene-Set-Enrichment Analysis 6](#_Toc300574500)

[External validation of stemness gene signature 8](#_Toc300574501)

[Simulations to justify similarity between stemness bipartition and basal-like breast cancer 9](#_Toc300574502)

[p53 mutant gene signatures 14](#_Toc300574503)

[References 16](#_Toc300574504)

[Figures 18](#_Toc300574505)

[Tables 19](#_Toc300574506)

This information will also be available on our group website <http://compbio.dfci.harvard.edu/> and http://bcb.dfci.harvard.edu/~aedin

Corresponding author contact details:

Aedín Culhane

Dana-Farber Cancer Institute

450 Brookline Ave, Smith 822C

Boston, Massachusetts 02115

Phone: (617) 632-2468

Fax: (617) 582-7760

Email: aedin@jimmy.harvard.edu

homepage: <http://www.hsph.harvard.edu/aedin-culhane/>

## Software

All analysis was performed using R version 2.10.1.

## Data pre-processing

Several independent datasets were used for analysis and validation. These were the Australian Ovarian Cancer Study (AOCS) dataset [[1](#_ENREF_1)], GSE9891, obtained on Affymetrix GeneChip U133 plus 2.0 arrays and downloaded from GEO [[2](#_ENREF_2)]; the Dressman et al. ovarian cancer dataset [[3](#_ENREF_3)] obtained on Affymetrix GeneChip U133a arrays, which was downloaded from the authors’ listed website (<http://data.cgt.duke.edu/platinum.php>) on August 23, 2010; the Crijns et al. ovarian cancer dataset [[4](#_ENREF_4)], which was obtained on Operon human v3 ~35K 70-mer two-color oligonucleotide microarrays, and which is available as GSE13876 on GEO, but which the authors emailed to us in normalized form; the Wu et al. ovarian cancer dataset, GSE6008, which was obtained on Affymetrix GeneChip U133a arrays and downloaded from GEO [[5](#_ENREF_5)]; the Tone et al. ovarian cancer dataset, GSE10971, which was obtained on Affymetrix GeneChip U133 plus 2.0 arrays and downloaded from GEO [[6](#_ENREF_6)]; The Cancer Genome Atlas (TCGA) ovarian cancer microarray dataset [[7](#_ENREF_7)], which was obtained on Affymetrix GeneChip U133a arrays and downloaded on November 24, 2010 using the TCGA data portal (<https://tcga-data.nci.nih.gov/tcga/>); the Miller et al. breast cancer dataset, GSE3494, which was obtained on Affymetrix GeneChip U133a arrays and downloaded from GEO [[8](#_ENREF_8)]; the Desmedt et al. breast cancer dataset, GSE7390, which was obtained on Affymetrix GeneChip U133a arrays and downloaded from GEO [[9](#_ENREF_9)]; and a merged breast cancer dataset combining GSE2034 [[10](#_ENREF_10)] and GSE5327 [[11](#_ENREF_11)], which we called the “Veridex” dataset and which was also downloaded from GEO.

All datasets were processed with an Ensembl custom cell description file (CDF), except for the Crijns dataset. The Crijns dataset was processed as described in the paper: quantile normalization, quality control for samples using principle components analysis, annotation with HGNC symbols, and averaging of Cy5- and Cy3-labeled samples [[4](#_ENREF_4)]. Otherwise, custom CDFs were used, because updated probe set definitions provide better precision and accuracy compared to Affymetrix probe set definitions [[12](#_ENREF_12)]. We used Version 12 of the Ensembl gene custom CDF provided by the University of Michigan Microarray Lab’s website: <http://brainarray.mbni.med.umich.edu/Brainarray/Database/CustomCDF/genomic_curated_CDF.asp>. Except for the Wu and Tone datasets, all those processed with an Ensembl custom CDF were processed with RMA [[13](#_ENREF_13)] [[14](#_ENREF_14)] because of RMA’s highly reproducible results and correlation with RT-PCR data [[15](#_ENREF_15)]. We specifically used the function $rma$ of the Bioconductor package “affy” [[16](#_ENREF_16)]. This function is an implementation of the robust multichip average (RMA) [[13](#_ENREF_13)]. Due to the inclusions of normal fallopian tube in the Tone dataset and normal ovarian surface epithelium samples in the Wu dataset, both datasets were normalized using the Invariant Set normalization method [[17](#_ENREF_17)] to avoid the assumption of equivalent gene expression distribution in each array. This was performed with the function $expresso$ in the Bioconductor package “affy.”

After processing, each dataset was a matrix of several thousand genes annotated with Ensembl identifiers for patients’ samples. The entire AOCS (Tothill et al.) dataset had 18,456 genes and 285 patients. The Dressman dataset had 11,967 genes and 118 patients due to mismatch of one patient’s ID. The Wu dataset had 11,967 genes and 103 patients. The Tone dataset had 19,064 genes and 37 patients. The Crijns dataset had 15,909 genes and 157 patients. The TCGA dataset had 11,967 genes and 518 patients. The Miller dataset had 11,967 genes and 251 patients. The Desmedt dataset had 11,967 genes and 198 patients. The Veridex dataset had 11,967 genes and 344 patients.

## AOCS dataset clustering

We discovered bipartitions in the patients of a subset of the entire AOCS dataset using a method that generates candidate bipartitions in patients called ISIS (“Identifying splits with clear separation”) [[18](#_ENREF_18)]. Both the patients and genes in this analysis were limited. The patients were limited to those that did not receive neoadjuvant therapy and had malignant serous tumors that were arrayed at the ovary and for which primary site was the ovary.

The genes used for patient clustering with ISIS were limited to human and mouse stem cell genes downloaded from GeneSigDB [[19](#_ENREF_19)]. The mouse stem cell genes were converted to human orthologs with the function $getLDS$ in the “biomaRt” package of Bioconductor, specifically with “host” parameter equal to “may2010.archive.ensembl.org,” which corresponds to Ensembl genome 58, for consistency. Otherwise, all annotation conversion was performed with the latest Ensembl genome version at the time with the function $getBM$ in the Bioconductor package “biomaRt.” Of the gene signatures from GeneSigDB, we only used signatures with greater than 5 Ensembl ID’s and fewer than 1,000 Ensembl ID’s in an attempt to find gene signatures that sufficiently characterized stem cells while avoiding the noise common in large gene sets. We also excluded Table A13 and Table A14 from Murat et al. [[20](#_ENREF_20)], because they were not stem cell gene signatures.

Proliferation genes are strongly associated with grade and have been shown to inflate statistical significant of stem-cell gene signature analysis [[21](#_ENREF_21)]. Therefore, we removed genes that were coordinately expressed and associated with proliferation (Table S7). Rather than select a single published gene signature or gene ontology category, we combined genes from several gene lists from gene ontology, KEGG, Biocarta, GeneSigDB [[19](#_ENREF_19),[22](#_ENREF_22)] and MSigDB [[23](#_ENREF_23)] that were associated with grade and proliferation in gene set enrichment analysis (GSEA) of multiple cancers. To identify a set of genes most robustly associated with proliferation across cancers, we applied meta-gene set analysis using iBBiG [[24](#_ENREF_24)] and GSEA [[23](#_ENREF_23)] to identify a proliferation gene sets module. In this analysis, 2,854 gene signatures from the Gene Signature Database (GeneSigDB) [[19](#_ENREF_19)] and Molecular Signature Database (MSigDB) [[23](#_ENREF_23)] were tested in 14,103 gene expression profiles of cancer, representing 22 different tissue types, from GCOD, our in-house database of cancer related gene expression datasets [[25](#_ENREF_25)]. This resulted in a large matrix of p-values, which was discretized and subjected to iBBiG bi-cluster analysis to identify geneset modules [[24](#_ENREF_24)]. The largest and highest ranked gene set module was strongly associated with proliferation and tumor grade. We extracted the set of genes which were both present in the most highly ranked proliferation gene sets (n = 100) and were also differentially expressed using limma [[26](#_ENREF_26)] ( p < 0.05 [[26](#_ENREF_26)] among module clinical covariates. This resulted in a list of 1,822 genes (Table S7) that were associated with an elevated proliferation across multiple tumors or cancerous tissues. 580 of these genes were also among the stem cell genes downloaded from GeneSigDB and were these were excluded from subsequent analysis.

## Statistical analysis

Analysis was performed with all available data and no imputation for missing data. Unless otherwise specified, p-values for association between a bipartition and an ordered categorical (grade and stage) or continuous variable (age) with a bipartition were calculated using likelihood ratio test for logistic regression, with the clinical variable predicting the bipartition. P-values for association between unordered categorical variables (residual disease, arrayed site, primary site) and a bipartition were generated by creating a contingency table and performing Fisher’s exact test. P-values for a gene’s expression’s relationship with a binary and ordered categorical variable were generated with the likelihood ratio tests for logistic regression and ordinal logistic regression, respectively. P-values appearing in Kaplan-Meier curves were generated with the logrank test with the function $survdiff$ in the R package “survival” [[27](#_ENREF_27)], and p-values for multivariate survival analysis were generated using Cox proportional hazards regression (function *coxph* in “survival”).

In the AOCS dataset, the clinical variable describing the arrayed site had levels ovary, peritoneum, and other because of small group size for all arrayed sites that were not ovary or peritoneum. Residual disease was coded as a binary variable with residual disease greater than 1 cm coded as 1 and less than 1cm coded as 0.

Except for in Gene-Set-Enrichment Analysis and when otherwise specified, when conducting many non-nested tests, we corrected the p-values for the family-wise error rate using Hommel’s method [[28](#_ENREF_28)] with the function *p.adjust* in the Bioconductor package “limma.” This was because the joint interpretation of a set of tests was of interest and for more conservative p-value estimation. When multiple testing correction was applied, but the joint interpretation was not necessarily of interest and less conservative multiple testing correction was sufficient, we corrected p-values using the false discovery rate [[29](#_ENREF_29)] with *p.adjust*.

## Gene-Set-Enrichment Analysis

To characterize genes enriched in the classes of the stemness bipartition of the subset of AOCS patients used for ISIS clustering, we used Gene-Set-Enrichment Analysis (GSEA) with nonparametric inference for linear models, and specifically the implementation $gsealmPerm$ in the Bioconductor package “GSEAlm” [[30](#_ENREF_30)]. We performed 100,000 permutations to compute approximate p-values. Prior to GSEA, Ensembl identifiers were converted to gene symbols so to be comparable to the gene sets from MSigDB [[23](#_ENREF_23)] and to be comparable to stem cell gene sets used by Ben-Porath et al. [[21](#_ENREF_21)]. Gene identifier conversion was done with the function *getBM* of the Bioconductor package “biomaRt.” When an Ensembl identifier mapped uniquely to multiple gene symbols, the gene symbols were combined in GSEA. When Ensembl identifiers mapped to the exact same gene symbols, their gene expression values were averaged.

The stem cell gene sets used by Ben-Porath et al. were as follows (Table S3):

1. Embryonic stem cell gene sets: two gene sets over-expressed in embryonic stem cells compared to other cells and tissues [[31](#_ENREF_31)].
2. Nanog, Oct4, and Sox2 (NOS) targets: four gene sets whose promoters are bound and activated in human ES cells by each of these regulators of ES cell identity, or co-activated by all three [[32](#_ENREF_32)], and an additional gene set (NOS TFs) that is the subset of NOS activation targets that encode transcription regulators [[21](#_ENREF_21)].
3. Polycomb targets: four sets representing genes bound by the Polycomb repressive complex 2 (PRC2) in human ES cells [[33](#_ENREF_33)].
4. Myc targets: two gene sets bound and activated by c-Myc, identified in two independent studies [[34](#_ENREF_34)] [[35](#_ENREF_35)].

The proliferation genes excluded from a second GSEA were the same as those used by Ben-Porath et al. and consisted of genes functionally involved in proliferation according to Gene Ontology annotations [[21](#_ENREF_21)], genes showing cell cycle stage-specific expression [[36](#_ENREF_36)], and genes belonging to a “proliferation cluster” defined in human breast tumor expression data [[37](#_ENREF_37)].

Despite enrichment in gene expression of the targets of Nanog, Oct4, and Sox2 in the stem-like subtype (Schwede et al., Table 1), we did not find that that these genes were differentially expressed between the stem-like and differentiated subtypes. Ben-Porath et al. similarly noted that known central regulators of embryonic stem cell identity—Oct4, Nanog, Sox2, Stat3, and Lin28—were not differentially expressed in high-grade breast cancer (27). Other stem cell-associated genes (CD24, CD44, PROM1, MYC) and epithelial-mesenchymal transition (EMT) markers (SNAI1, SNAI2, GSC, TWIST1, FN1, VIM, CDH1) were also not differentially expressed (p > 0.05 after FDR correction) between the stem-like and differentiated subtypes in the AOCS dataset.

## External validation of stemness gene signature

For validation, we reproduced the ISIS-generated stemness bipartition from the AOCS dataset in independent gene expression datasets. We learned diagonal linear discriminant (DLD) classifiers [[38](#_ENREF_38)] on the subset of the AOCS dataset used for ISIS clustering. This was performed using genes that ISIS used to score the patient bipartition (Table S2). The 95 genes that ISIS used were limited to the 51 that were in all 145 iterations of leave-one-patient-out cross-validation in the subset of the AOCS dataset used for clustering (n = 145). The DLD classifier assigns weights to each gene for a certain bipartition as follows:

|  | ${weight}_{g}\left( B \right)=\frac{\boldsymbol{\mu}_{M}\boldsymbol{-}\boldsymbol{\mu}_{N}}{s_{p}}$ |  |
| --- | --- | --- |

Where

|  | $s_{p}=\frac{\left( m-1 \right)\sigma_{gM}^{2}+\left( n-1 \right)\sigma_{gN}^{2}}{n+m-2}$ |  |
| --- | --- | --- |

${weight}_{g}\left( B \right)$ is the weight for a gene $g$ for bipartition $B$. $\boldsymbol{\mu}_{M}$ $and$ $\sigma_{gM}^{2}$ are the mean and variance, respectively, for gene $g$ in class $M$ (size $m$) of the bipartition, and so are $\boldsymbol{\mu}_{N}$ and $\sigma_{gN}^{2}$ for the other class, $N$ (size $n$).

To reproduce bipartitions in other datasets, due to different numbers of genes across datasets, we limited classifiers to the genes of the 51-gene signature that were in each independent dataset. The classifiers were a linear combination of gene expression values, using weights for each gene, which resulted in a score for each patient in each validation dataset. Scores were then grouped into clusters with Gaussian mixture modeling using the function $Mclust$ in R package “mclust” [[39](#_ENREF_39)]. Gaussian mixture modeling was used because in the ovarian cancer datasets, the DLD score was clearly bimodal (Fig. S1). Additionally, using mixture modeling allowed us to use a probability of class membership in class assignment, although we did not use probability restrictions, except for > 0.5 and < 0.5, because of the already low size of the differentiated subtype across datasets. Also, predicting classes using mixture modeling assumes that both classes are in a given dataset. We chose to make this assumption rather than using a numerical cutoff for the DLD score because the DLD score range and center varied greatly across datasets due to platform differences and potential technical differences when generating the datasets [[40](#_ENREF_40)].

Default parameters of $Mclust$ were used except for when reproducing the bipartition in the Desmedt, Veridex, and Miller breast cancer datasets. In these cases, the number of mixture components was specified to be 2 because the DLD score distribution in the breast cancer datasets was not obviously bimodal. Otherwise, $Mclust$ chose the optimal model among 1 to 9 mixture components according to the Bayesian Information Criterion (BIC). The fact that 2 components was optimal in the Crijns, Dressman, Tone, Wu, and “remaining” AOCS datasets provides evidence that in these datasets, there were indeed two classes for the DLD score. This is expected, because the analysis was meant to construct two subtypes. The need to specify the number of mixture components in the breast cancer datasets was likely due to cross-tissue differences in the breast cancer datasets.

## Simulations to justify similarity between stemness bipartition and basal-like breast cancer

After reproducing a bipartition in breast cancer datasets and realizing similarity to the basal-like subtype, we performed two sets of simulations to justify the specific similarity of the stemness bipartition to a basal/non-basal bipartition in the breast cancer datasets. The highly distinct gene expression patterns associated with estrogen receptor in breast cancer [[41](#_ENREF_41)] suggests that many gene expression patterns are related to basal-like breast cancer, and so the specific similarity of the stemness bipartition was in question.

Before the simulations, we generated two sets of DLD scores for the patients in the Desmedt breast cancer dataset. One set consisted of DLD scores trained on the stemness bipartition in the AOCS dataset, and the other set consisted of DLD scores trained on the basal/non-basal bipartition in the Desmedt dataset. The stemenss DLD score, as previously described, consisted of a weighted sum of 51 genes’ expression. In contrast, the basal/non-basal bipartition’s scores were a weighted sum of 41 genes strongly associated with the basal-like subtype. These select genes were the top 44 genes most differentially expressed in the basal-like subtype except for the top three most associated genes. This is because 41 out of the 51 genes used for classification were also in the Desmedt dataset due to platform differences, and three of the five genes most associated with the stemness bipartition (corresponding to ISIS parameter “p.offs”) were also in the Desmedt dataset. We then recorded the Spearman correlation between the basal-like subtype’s DLD scores and the stemness bipartition’s DLD score for comparison with simulated scores.

In the first simulation, we wanted to test the specificity of our bipartition’s genes’ relationship with the basal subtype. We created 10,000 random bipartitions in the AOCS dataset, and for each bipartition, we calculated DLD scores in a breast cancer dataset again using 41 genes that were in the Desmedt dataset due to cross platform differences. Random bipartitions were generated using a beta-binomial model where the beta parameters were estimated with maximum likelihood estimation (MLE). The data used for the MLE were the proportions of patients in bipartition classes in the original ISIS run for class discovery. Random bipartitions’ classes never had fewer than 10 patients. After calculating DLD scores for each patient in the Desmedt dataset, we then computed Spearman correlation between each bipartition’s scores and the basal subtype’s scores in the Desmedt dataset. This resulted in a distribution of Spearman correlations between random bipartitions in the AOCS dataset and the basal/non-basal bipartition in the Desmedt dataset. Thus, we generated an approximate two-sided p-value for the stemness bipartition’s correlation with the basal subtype by using this distribution of correlations.

In the second simulation, we tested the specificity of the stemness bipartition, given the stemness bipartition’s genes. We again generated 10,000 random bipartitions and this time trained these on the same 41 genes that were used to generate the stemness bipartition in the Desmedt dataset. We computed Spearman correlation between these bipartition’s genes’ scores and the basal subtype’s scores. Again, an approximate two-sided p-value for our bipartition’s specific relationship with the basal subtype, given the 51-gene signature, was generated by considering the distribution of these absolute Spearman correlations.

In the first simulation, the stemness bipartition’s scores had higher absolute correlation with the Desmedt dataset’s basal subtype’s DLD scores than all but 0.02% of random bipartitions. In the second simulation, the stemness bipartition’s scores were still more correlated with the basal subtype’s similar scores than all but 0.59% of random bipartitions. These simulations provide further evidence that both the genes associated with the stemness bipartition and the bipartition itself are specifically similar to basal-like breast cancer.

## p53 mutant gene signatures

To analyze enrichment for p53 mutations, we analyzed two gene signatures on GeneSigDB [[19](#_ENREF_19)] that purportedly characterize p53 mutants in breast cancer (Table S6). GeneSigDB provided the Ensembl identifiers for each signature that were used in analysis in the AOCS dataset. The first signatures was the 52-gene signature in Fig. 5 of Troester et al., which was developed to classify p53 mutants from p53 wildtype breast cancer [[42](#_ENREF_42)]. Melissa Troester emailed the signature directly to us. 49 of these genes were in the AOCS dataset and were used for analysis. The second was a 32-gene signature that was again a predictor of p53 mutation status in breast cancer [[43](#_ENREF_43)]. All 32 genes were in the AOCS dataset. We considered both how each gene related to the bipartition using the likelihood ratio test for logistic regression and how the gene set overall related using Gene-Set-Enrichment Analysis, specifically GSEA with nonparametric inference for linear models, as already described [[30](#_ENREF_30)].

## References

1. Tothill RW, Tinker AV, George J, Brown R, Fox SB, et al. (2008) Novel molecular subtypes of serous and endometrioid ovarian cancer linked to clinical outcome. Clin Cancer Res 14: 5198-5208.

2. Edgar R, Domrachev M, Lash AE (2002) Gene Expression Omnibus: NCBI gene expression and hybridization array data repository. Nucleic Acids Res 30: 207-210.

3. Dressman HK, Berchuck A, Chan G, Zhai J, Bild A, et al. (2007) An integrated genomic-based approach to individualized treatment of patients with advanced-stage ovarian cancer. J Clin Oncol 25: 517-525.

4. Crijns AP, Fehrmann RS, de Jong S, Gerbens F, Meersma GJ, et al. (2009) Survival-related profile, pathways, and transcription factors in ovarian cancer. PLoS Med 6: e24.

5. Wu R, Hendrix-Lucas N, Kuick R, Zhai Y, Schwartz DR, et al. (2007) Mouse model of human ovarian endometrioid adenocarcinoma based on somatic defects in the Wnt/beta-catenin and PI3K/Pten signaling pathways. Cancer Cell 11: 321-333.

6. Tone AA, Begley H, Sharma M, Murphy J, Rosen B, et al. (2008) Gene expression profiles of luteal phase fallopian tube epithelium from BRCA mutation carriers resemble high-grade serous carcinoma. Clin Cancer Res 14: 4067-4078.

7. Cancer Genome Atlas Research Network (2011) Integrated genomic analyses of ovarian carcinoma. Nature 474: 609-615.

8. Miller LD, Smeds J, George J, Vega VB, Vergara L, et al. (2005) An expression signature for p53 status in human breast cancer predicts mutation status, transcriptional effects, and patient survival. Proc Natl Acad Sci U S A 102: 13550-13555.

9. Desmedt C, Piette F, Loi S, Wang Y, Lallemand F, et al. (2007) Strong time dependence of the 76-gene prognostic signature for node-negative breast cancer patients in the TRANSBIG multicenter independent validation series. Clin Cancer Res 13: 3207-3214.

10. Wang Y, Klijn JG, Zhang Y, Sieuwerts AM, Look MP, et al. (2005) Gene-expression profiles to predict distant metastasis of lymph-node-negative primary breast cancer. Lancet 365: 671-679.

11. Minn AJ, Gupta GP, Padua D, Bos P, Nguyen DX, et al. (2007) Lung metastasis genes couple breast tumor size and metastatic spread. Proc Natl Acad Sci U S A 104: 6740-6745.

12. Sandberg R, Larsson O (2007) Improved precision and accuracy for microarrays using updated probe set definitions. BMC Bioinformatics 8: 48.

13. Irizarry RA, Hobbs B, Collin F, Beazer-Barclay YD, Antonellis KJ, et al. (2003) Exploration, normalization, and summaries of high density oligonucleotide array probe level data. Biostatistics 4: 249-264.

14. Irizarry RA, Bolstad BM, Collin F, Cope LM, Hobbs B, et al. (2003) Summaries of Affymetrix GeneChip probe level data. Nucleic Acids Res 31: e15.

15. Millenaar FF, Okyere J, May ST, van Zanten M, Voesenek LA, et al. (2006) How to decide? Different methods of calculating gene expression from short oligonucleotide array data will give different results. BMC Bioinformatics 7: 137.

16. Gentleman RC, Carey VJ, Bates DM, Bolstad B, Dettling M, et al. (2004) Bioconductor: open software development for computational biology and bioinformatics. Genome Biol 5: R80.

17. Li C, Wong WH (2001) Model-based analysis of oligonucleotide arrays: expression index computation and outlier detection. Proc Natl Acad Sci U S A 98: 31-36.

18. von Heydebreck A, Huber W, Poustka A, Vingron M (2001) Identifying splits with clear separation: a new class discovery method for gene expression data. Bioinformatics 17 Suppl 1: S107-114.

19. Culhane AC, Schwarzl T, Sultana R, Picard KC, Picard SC, et al. (2009) GeneSigDB--a curated database of gene expression signatures. Nucleic Acids Res.

20. Murat A, Migliavacca E, Gorlia T, Lambiv WL, Shay T, et al. (2008) Stem cell-related "self-renewal" signature and high epidermal growth factor receptor expression associated with resistance to concomitant chemoradiotherapy in glioblastoma. J Clin Oncol 26: 3015-3024.

21. Ben-Porath I, Thomson MW, Carey VJ, Ge R, Bell GW, et al. (2008) An embryonic stem cell-like gene expression signature in poorly differentiated aggressive human tumors. Nat Genet 40: 499-507.

22. Culhane AC, Schroder MS, Sultana R, Picard SC, Martinelli EN, et al. (2012) GeneSigDB: a manually curated database and resource for analysis of gene expression signatures. Nucleic Acids Res 40: D1060-1066.

23. Subramanian A, Tamayo P, Mootha VK, Mukherjee S, Ebert BL, et al. (2005) Gene set enrichment analysis: a knowledge-based approach for interpreting genome-wide expression profiles. Proc Natl Acad Sci U S A 102: 15545-15550.

24. Gusenleitner D, Howe EA, Bentink S, Quackenbush J, Culhane AC (2012) iBBiG: Iterative Binary Bi-clustering of Gene Sets. Bioinformatics. In Press

25. Liu F, White JA, Antonescu C, Gusenleitner D, Quackenbush J (2011) GCOD - GeneChip Oncology Database. BMC Bioinformatics 12: 46.

26. Smyth GK (2004) Linear models and empirical bayes methods for assessing differential expression in microarray experiments. Stat Appl Genet Mol Biol 3: Article3.

27. Harrington DPaF, Thomas R. (1982) A Class of Rank Test Procedures for Censored Survival Data. Biometrika 69: 553-566.

28. Hommel G (1988) A Stagewise Rejective Multiple Test Procedure Based on a Modified Bonferroni Test. Biometrika 75: 383-386.

29. Benjamini Y, Hochberg Y (1995) Controlling the False Discovery Rate: A Practical and Powerful Approach to Multiple Testing. Journal of the Royal Statistical Society Series B (Methodological) 57: 289-300.

30. Jiang Z, Gentleman R (2007) Extensions to gene set enrichment. Bioinformatics 23: 306-313.

31. Assou S, Le Carrour T, Tondeur S, Strom S, Gabelle A, et al. (2007) A meta-analysis of human embryonic stem cells transcriptome integrated into a web-based expression atlas. Stem Cells 25: 961-973.

32. Boyer LA, Lee TI, Cole MF, Johnstone SE, Levine SS, et al. (2005) Core transcriptional regulatory circuitry in human embryonic stem cells. Cell 122: 947-956.

33. Lee TI, Jenner RG, Boyer LA, Guenther MG, Levine SS, et al. (2006) Control of developmental regulators by Polycomb in human embryonic stem cells. Cell 125: 301-313.

34. Fernandez PC, Frank SR, Wang L, Schroeder M, Liu S, et al. (2003) Genomic targets of the human c-Myc protein. Genes Dev 17: 1115-1129.

35. Li Z, Van Calcar S, Qu C, Cavenee WK, Zhang MQ, et al. (2003) A global transcriptional regulatory role for c-Myc in Burkitt's lymphoma cells. Proc Natl Acad Sci U S A 100: 8164-8169.

36. Whitfield ML, Sherlock G, Saldanha AJ, Murray JI, Ball CA, et al. (2002) Identification of genes periodically expressed in the human cell cycle and their expression in tumors. Mol Biol Cell 13: 1977-2000.

37. Hu Z, Fan C, Oh DS, Marron JS, He X, et al. (2006) The molecular portraits of breast tumors are conserved across microarray platforms. BMC Genomics 7: 96.

38. Dudoit S, J. Fridlyand, and T. P. Speed (2002) Comparison of Discrimination Methods for the Classification of Tumors Using Gene Expression Data. Journal of the American Statistical Association 97: 77-87.

39. Fraley CaR, Adrian E. (2002) Model-Based Clustering, Discriminant Analysis, and Density Estimation. Journal of the American Statistical Association 97: 611-631.

40. Leek JT, Scharpf RB, Bravo HC, Simcha D, Langmead B, et al. (2010) Tackling the widespread and critical impact of batch effects in high-throughput data. Nat Rev Genet 11: 733-739.

41. Gruvberger S, Ringner M, Chen Y, Panavally S, Saal LH, et al. (2001) Estrogen receptor status in breast cancer is associated with remarkably distinct gene expression patterns. Cancer Res 61: 5979-5984.

42. Troester MA, Herschkowitz JI, Oh DS, He X, Hoadley KA, et al. (2006) Gene expression patterns associated with p53 status in breast cancer. BMC Cancer 6: 276.

43. Takahashi S, Moriya T, Ishida T, Shibata H, Sasano H, et al. (2008) Prediction of breast cancer prognosis by gene expression profile of TP53 status. Cancer Sci 99: 324-332.

**
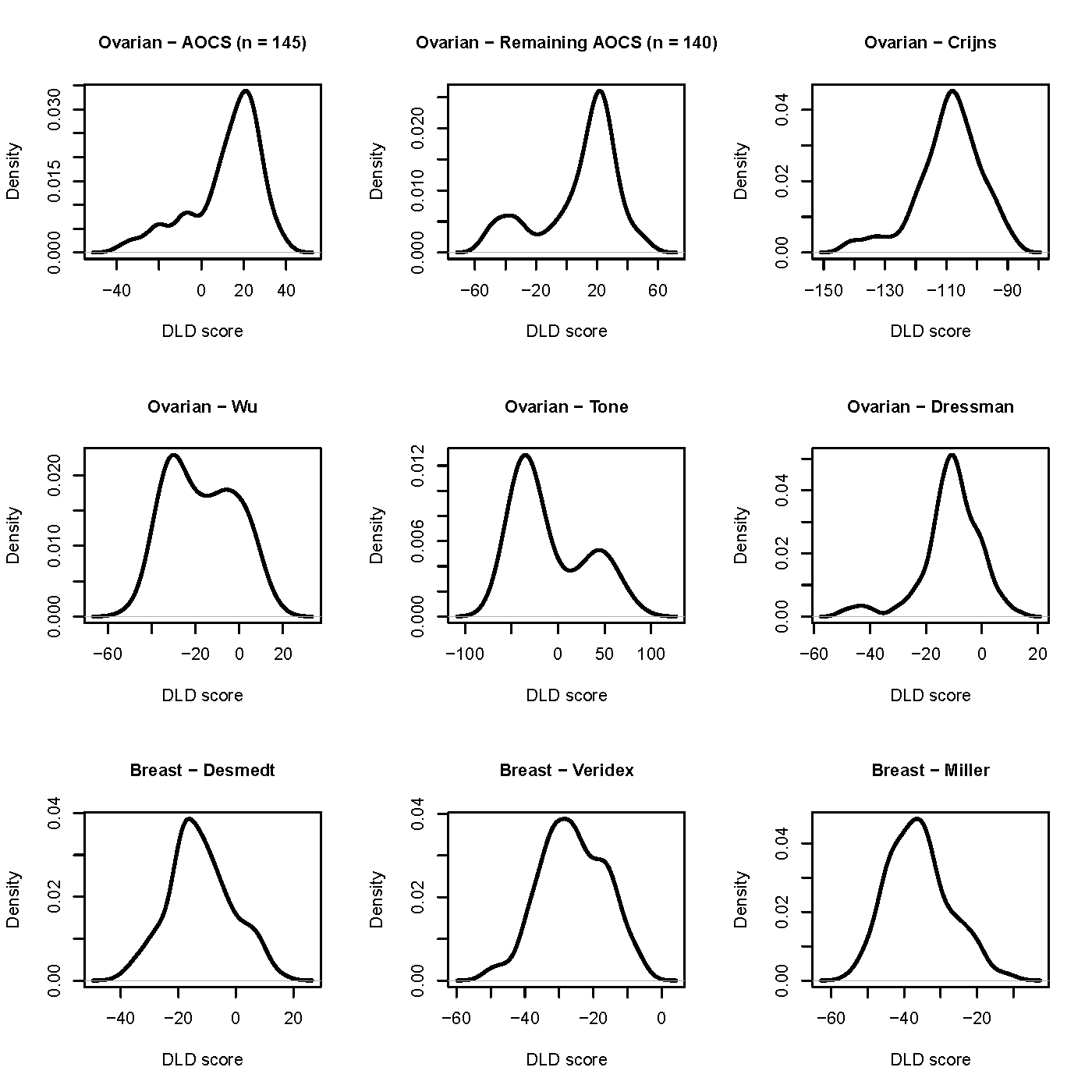
**

## Figures

**Fig. S1**: Distributions of stemness bipartition diagonal linear discriminant (DLD) scores in each dataset. In the ovarian cancer datasets, the DLD scores were all bimodal, and Gaussian mixture modeling [[39](#_ENREF_39)] of the score was used to classify the lower scoring group as differentiated and the higher scoring group as stem-like. In the breast cancer datasets, mixture modeling was still used to discover approximate stem-like and differentiated classes, despite lack of clear bimodality. The range of scores is inconsistent in different datasets, because genes were often lost when crossing microarray platforms and potentially because of batch effects across datasets.

## Tables

**Table S1:** A list of the 83 gene signatures from GeneSigDB [[19](#_ENREF_19)] that are reported to be associated with stem cells. Data are provided in MSigDB gmt and gmx file format on <http://bcb.dfci.harvard.edu/~aedin/publications/>

| **Gene Signature** | | | | **Article** | | | | |
| --- | --- | --- | --- | --- | --- | --- | --- | --- |
| **GeneSigDB ID** | **Organism** | **Description** | **Platform** | **PMID** | **First Author** | **Journal** | **Year** | **Title** |
| 15941854-table1 | Human | Table 1. Genes upregulated in slowly growing clones compared with fast-growing clones derived from individual CD34+CD38- cells from human umbilical cord blood | Affy U133A | 15941854 | K Bartolovic | Stem Cells | 2005 | Clonal heterogeneity in growth kinetics of CD34+CD38- human cord blood cells in vitro is correlated with gene expression pattern and telomere length. |
| 15941854-table2 | Human | Table 2. Genes downregulated in slowly growing clones compared with fast-growing clones derived from individual CD34+CD38- cells from human umbilical cord blood | Affy U133A | 15941854 | K Bartolovic | Stem Cells | 2005 | Clonal heterogeneity in growth kinetics of CD34+CD38- human cord blood cells in vitro is correlated with gene expression pattern and telomere length. |
| 15992799-table1 | Mouse | Table 1 Homologous expression between undifferentiated C17.2 NSCs, ESCs, HSCs, and CNS-derived neurospheres | Affy mg u74a | 15992799 | MA Parker | Exp Neurol | 2005 | Expression profile of an operationally-defined neural stem cell clone. |
| 15992799-table2 | Mouse | Table 2 Homologous expression between C17.2 NSC-derived neurospheres, ESCs, HSCs, and CNS-derived neurospheres | Affy mg u74a | 15992799 | MA Parker | Exp Neurol | 2005 | Expression profile of an operationally-defined neural stem cell clone. |
| 16014681-table2 | Human | Table 2 Down regulated genes expressed in PBMC from patients with SSc at 6 months after HSCT as indicated by a cDNA array | Custom cDNA Array | 16014681 | T Bohgaki | Ann Rheum Dis | 2005 | Up regulated expression of tumour necrosis factor alpha converting enzyme in peripheral monocytes of patients with early systemic sclerosis. |
| 16140871-Table1 | Human | Table 1. Genes upregulated by more than fivefold in CD133+ CB and PB cells compared with CD133- cells | affy U133A | 16140871 | A Toren | Stem Cells | 2005 | CD133-positive hematopoietic stem cell \stemness\" genes contain many genes mutated or abnormally expressed in leukemia." |
| 16140871-SuppTable7 | Human | Supplemental Table 7. Genes down-regulated by more than 2 fold in CD133+ as compared to CD133- cells | affy U133A | 16140871 | A Toren | Stem Cells | 2005 | CD133-positive hematopoietic stem cell \stemness\" genes contain many genes mutated or abnormally expressed in leukemia." |
| 16166251-table1 | Human | Table 1. First 50 most upregulated genes in CD34+ hematopoietic cells expanded in a collagen matrix | affy U133A | 16166251 | J Oswald | Stem Cells | 2006 | Gene-expression profiling of CD34+ hematopoietic cells expanded in a collagen I matrix. |
| 16188652-table1a | Human | Table la. Quartile 4 FDR 2.5% gene probe sets ? rank correlations with Iq21 amplification index, CKSIB and PC labeling index and adjusted P-values for associations with overall survival | affy U133 Plus2 | 16188652 | J Shaughnessy | Hematology | 2005 | Amplification and over expression of CKS1B at chromosome band 1q21 is associated with reduced levels of p27Kip1 and an aggressive clinical course in multiple myeloma. |
| 16188652-table1b | Human | Table Ib. Quartile 1 gene probe sets satisfying FDR 2.5% cutoff | affy U133 Plus2 | 16188652 | J Shaughnessy | Hematology | 2005 | Amplification and over expression of CKS1B at chromosome band 1q21 is associated with reduced levels of p27Kip1 and an aggressive clinical course in multiple myeloma. |
| 16210406-table1 | Human | Table 1. The genes representing the most significant biological processes in CD133+ cells | affy U133 Plus2 | 16210406 | T Jaatinen | Stem Cells | 2006 | Global gene expression profile of human cord blood-derived CD133+ cells. |
| 16210406-tableS4 | Human | Supplementary Table 4. Genes encoding putative membrane proteins in CD133+ cells. | affy U133 Plus2 | 16210406 | T Jaatinen | Stem Cells | 2006 | Global gene expression profile of human cord blood-derived CD133+ cells. |
| 16319131-table1 | Human | Tableÿ1. Human imprinted genes identified by GeneChip | affy U133A | 16319131 | BW Sun | Hum Mol Genet | 2006 | Temporal and parental-specific expression of imprinted genes in a newly derived Chinese human embryonic stem cell line and embryoid bodies. |
| 16355723-table2a | Human | TABLE 2a. GENES THAT WERE FOUND TO BE UPREGULATED AND DOWNREGULATED IN MSC VERSUS SKIN FIBROBLASTS | Custom cDNA Array | 16355723 | C Brendel | Gene Expr | 2005 | Distinct gene expression profile of human mesenchymal stem cells in comparison to skin fibroblasts employing cDNA microarray analysis of 9600 genes. |
| 16355723-table2b | Human | TABLE 2b. GENES THAT WERE FOUND TO BE UPREGULATED AND DOWNREGULATED IN MSC VERSUS SKIN FIBROBLASTS | Custom cDNA Array | 16355723 | C Brendel | Gene Expr | 2005 | Distinct gene expression profile of human mesenchymal stem cells in comparison to skin fibroblasts employing cDNA microarray analysis of 9600 genes. |
| 16414199-table2 | Human | Table 2. Signaling pathways and molecules involved in hNSC priming | Custom cDNA Array | 16414199 | Y Cai | Neuroscience | 2006 | Gene expression profiling and analysis of signaling pathways involved in priming and differentiation of human neural stem cells. |
| 16414199-table3 | Human | Table 3. Genes changed in opposite directions during priming and differentiation | Custom cDNA Array | 16414199 | Y Cai | Neuroscience | 2006 | Gene expression profiling and analysis of signaling pathways involved in priming and differentiation of human neural stem cells. |
| 16546165-table2 | Mouse | Table 2. UniGene clusters with the highest representations in the three stem cell cDNA libraries | affy Mouse 430 2 | 16546165 | C Williams | Exp Cell Res | 2006 | Catalog of gene expression in adult neural stem cells and their in vivo microenvironment. |
| 16546165-table5 | Mouse | Table 5. Genes expected to be expressed in the neural stem/progenitor cells | affy Mouse 430 2 | 16546165 | C Williams | Exp Cell Res | 2006 | Catalog of gene expression in adult neural stem cells and their in vivo microenvironment. |
| 16581771-tableS1a | Mouse | Table S1 (111 Positive Significant Genes >2.5 fold), upon Wnt3A stimulation, the expression of 220 genes changed more than 2.5-fold, 111 of which were significantly up-regulated and 109 of which were drastically down-regulated | affy Mouse 430 2 | 16581771 | W Si | Mol Cell Biol | 2006 | CCN1/Cyr61 is regulated by the canonical Wnt signal and plays an important role in Wnt3A-induced osteoblast differentiation of mesenchymal stem cells. |
| 16581771-tableS1b | Mouse | Table S1 (109 Negative Significant Genes >2.5 fold), Some of the top functions induced by Wnt3A in mesenchymal stem cells include cellular growth and proliferation | affy Mouse 430 2 | 16581771 | W Si | Mol Cell Biol | 2006 | CCN1/Cyr61 is regulated by the canonical Wnt signal and plays an important role in Wnt3A-induced osteoblast differentiation of mesenchymal stem cells. |
| 16675456-table1 | Mouse | TABLE 1 The top 50 differentially expressed genes in limbal versus corneal epithelial basal cells | affy Mouse 430 2 | 16675456 | M Zhou | J Biol Chem | 2006 | Transcriptional profiling of enriched populations of stem cells versus transient amplifying cells. A comparison of limbal and corneal epithelial basal cells. |
| 16705683-table2 | Mouse | TABLE 2. List of Genes Upregulated in Mouse GS Cells Compared to ES Cells | Custom cDNA Array | 16705683 | RS Fujino | Mol Reprod Dev | 2006 | Capillary morphogenesis gene (CMG)-1 is among the genes differentially expressed in mouse male germ line stem cells and embryonic stem cells. |
| 16705683-table3 | Mouse | TABLE 3. List of Genes Downregulated in Mouse GS Cells Compared to ES Cells | Custom cDNA Array | 16705683 | RS Fujino | Mol Reprod Dev | 2006 | Capillary morphogenesis gene (CMG)-1 is among the genes differentially expressed in mouse male germ line stem cells and embryonic stem cells. |
| 16799977-tableS1 | Human | Supplementary Table1. Upregulated (>2.0) genes in both Huh7 and PLC/PRF/5 SP cells | affy U133 Plus2 | 16799977 | T Chiba | Hepatology | 2006 | Side population purified from hepatocellular carcinoma cells harbors cancer stem cell-like properties. |
| 16863911-table1 | Human | Table 1. List of the 110 genes with differential expression (for increased expression: log2 ratio < 0.5, p < 0.0001; for decreased expression: log2 ratio > ?0.4, p < 0.0001) in AMD3100 + G-CSF-mobilized PBPC compared to G-CSF-mobilized cells | affy U133 Plus2 | 16863911 | S Fruehauf | Exp Hematol | 2006 | The CXCR4 antagonist AMD3100 releases a subset of G-CSF-primed peripheral blood progenitor cells with specific gene expression characteristics. |
| 16880536-table1 | Human | TABLE 1. qRT-PCR analysis of GLI1/EGF-regulated target gene expression in human keratinocytes | Custom cDNA Array | 16880536 | M Kasper | Mol Cell Biol | 2006 | Selective modulation of Hedgehog/GLI target gene expression by epidermal growth factor signaling in human keratinocytes. |
| 17009876-tableS3a | Mouse | Table S3a. Differentially expressed genes between lower side population (LSP) cells and upper side population (USP) cells | Affy mg u74av2 | 17009876 | CA Ramos | PLoS Genet | 2006 | Evidence for diversity in transcriptional profiles of single hematopoietic stem cells. |
| 17009876-tableS3b | Mouse | Table S3b. Differentially expressed genes between lower side population (LSP) cells and upper side population (USP) cells | Affy mg u74av2 | 17009876 | CA Ramos | PLoS Genet | 2006 | Evidence for diversity in transcriptional profiles of single hematopoietic stem cells. |
| 17053208-table1a | Human | Table 1a. Surface antigen gene expression, from a list of top 20 highly expressed genes encoding for putative membrane proteins in either hESCMSCs or hESCs: (Highly expressed membrane proteins in hESC-derived MSC over hESC) | Illumina Human WG-6 Bead Chips | 17053208 | Q Lian | Stem Cells | 2007 | Derivation of clinically compliant MSCs from CD105+, CD24- differentiated human ESCs. |
| 17053208-table1b | Human | Table 1b. Surface antigen gene expression, from a list of top 20 highly expressed genes encoding for putative membrane proteins in either hESCMSCs or hESCs: (Highly expressed membrane proteins in hESC over hESC-derived MSC) | Illumina Human WG-6 Bead Chips | 17053208 | Q Lian | Stem Cells | 2007 | Derivation of clinically compliant MSCs from CD105+, CD24- differentiated human ESCs. |
| 17170073-TableS1 | Mouse | Supplementary Table 1: Changes in gene expression at day 5 of differentiation compared to day 4: up-regulation | affy MOE 430A | 17170073 | NI zur Nieden | Mol Endocrinol | 2007 | Gene profiling on mixed embryonic stem cell populations reveals a biphasic role for beta-catenin in osteogenic differentiation. |
| 17170073-TableS2 | Mouse | Supplementary Table 2: Changes in gene expression at day 5 of differentiation compared to day 4: down-regulation | affy MOE 430A | 17170073 | NI zur Nieden | Mol Endocrinol | 2007 | Gene profiling on mixed embryonic stem cell populations reveals a biphasic role for beta-catenin in osteogenic differentiation. |
| 17170073-TableS3 | Mouse | Supplementary Table 3: Changes in gene expression over the time course of differentiation compared to time corresponding controls: up-regulation | affy MOE 430A | 17170073 | NI zur Nieden | Mol Endocrinol | 2007 | Gene profiling on mixed embryonic stem cell populations reveals a biphasic role for beta-catenin in osteogenic differentiation. |
| 17183690-Table1 | Mouse | Table 1. List of top 20 highly expressed genes encoding CD antigens generated by comparing gene expression profiles of E-RoSH and RoSH cells with E14 ESCs. | Illumina Human v2 | 17183690 | Q Lian | PLoS One | 2006 | Establishing clonal cell lines with endothelial-like potential from CD9(hi), SSEA-1(-) cells in embryonic stem cell-derived embryoid bodies. |
| 17617570-Table2 | Mouse | Table II. Genes expressed in MLN organizer cells >2-fold increase than in PP organizer cells | Affy mg u74av2 | 17617570 | M Okuda | J Immunol | 2007 | Distinct activities of stromal cells involved in the organogenesis of lymph nodes and Peyer's patches. |
| 17617570-Table3 | Mouse | Table III. Genes expressed in PP organizer cells >2-fold increase than in MLN organizer cells | Affy mg u74av2 | 17617570 | M Okuda | J Immunol | 2007 | Distinct activities of stromal cells involved in the organogenesis of lymph nodes and Peyer's patches. |
| 17676974-TableS4 | Mouse | Supplemental Table S4 - Genes Up in p53+/m compared to p53+/- HSC | affy MOE 430A | 17676974 | SM Chambers | PLoS Biol | 2007 | Aging hematopoietic stem cells decline in function and exhibit epigenetic dysregulation. |
| 17676974-TableS5 | Mouse | Supplemental Table S5 - Genes Up in p53+/- compared to p53+/m HSC | affy MOE 430A | 17676974 | SM Chambers | PLoS Biol | 2007 | Aging hematopoietic stem cells decline in function and exhibit epigenetic dysregulation. |
| 18005044-TableS3a | Human | Supplement 3a. Genes expressed differentially in odontoblasts and pulp tissue in selected Gene Ontology categories. (Odontoblasts) | affy U133A | 18005044 | V Pääkkönen | Int Endod J | 2008 | Comparative gene expression profile analysis between native human odontoblasts and pulp tissue. |
| 18005044-TableS3b | Human | Supplement 3b. Genes expressed differentially in odontoblasts and pulp tissue in selected Gene Ontology categories. (Pulp Tissue) | affy U133A | 18005044 | V Pääkkönen | Int Endod J | 2008 | Comparative gene expression profile analysis between native human odontoblasts and pulp tissue. |
| 18034892-Table1 | Human | Table 1: The approved gene symbols and names in reference to HUGO Gene Nomenclature | affy U133 Plus2 | 18034892 | DC Seo | Mol Cancer | 2007 | Gene expression profiling of cancer stem cell in human lung adenocarcinoma A549 cells. |
| 18034892-Table2 | Human | Table 2: Gene list up-regulated in SP cells compared to non-SP cells | affy U133 Plus2 | 18034892 | DC Seo | Mol Cancer | 2007 | Gene expression profiling of cancer stem cell in human lung adenocarcinoma A549 cells. |
| 18310505-Table1 | Human | Table 1. Gene Symbols and Titles (Only 69 genes that had an absolute value of at least 0.90 of the correlation coefficient were included in subsequent analysis) | affy U133A | 18310505 | I Matushansky | Am J Pathol | 2008 | A developmental model of sarcomagenesis defines a differentiation-based classification for liposarcomas. |
| 18310505-TableS6 | Human | Supplemental Table 6: GENES OVER EXPRESSED IN DAY21 DIFFERENTIATED hMSCs into adipocytes COMPARED TO DAY14 DIFFERENTIATING hMSCs. | affy U133A | 18310505 | I Matushansky | Am J Pathol | 2008 | A developmental model of sarcomagenesis defines a differentiation-based classification for liposarcomas. |
| 18310505-TableS8 | Human | Supplemental Table 8 (pathways represented by genes under expressed in dedifferentiated liposarcomas versus normal fat after excluding markers of differentiation genes) | affy U133A | 18310505 | I Matushansky | Am J Pathol | 2008 | A developmental model of sarcomagenesis defines a differentiation-based classification for liposarcomas. |
| 18332912-Table2 | Human | Table 2 Differentially expressed genes in chronic GVHD patients | CodeLink UniSet Human I Bioarray | 18332912 | SJ Oh | Bone Marrow Transplant | 2008 | Cell cycle and immune-related processes are significantly altered in chronic GVHD. |
| 18378853-Table3a | Human | Table 3a. Genes (including hypothetical genes/proteins and open reading frames) differentially expressed 2-fold or greater among the identified BAALC-associated gene-expression signature (Genes over expressed in the high BAALC group) | affy U133 Plus2 | 18378853 | C Langer | Blood | 2008 | High BAALC expression associates with other molecular prognostic markers, poor outcome, and a distinct gene-expression signature in cytogenetically normal patients younger than 60 years with acute myeloid leukemia: a Cancer and Leukemia Group B (CALGB) study. |
| 18378853-Table3b | Human | Table 3b. Genes (including hypothetical genes/proteins and open reading frames) differentially expressed 2-fold or greater among the identified BAALC-associated gene-expression signature (Genes under expressed in the high BAALC group) | affy U133 Plus2 | 18378853 | C Langer | Blood | 2008 | High BAALC expression associates with other molecular prognostic markers, poor outcome, and a distinct gene-expression signature in cytogenetically normal patients younger than 60 years with acute myeloid leukemia: a Cancer and Leukemia Group B (CALGB) study. |
| 18395814-Table1a | Human | Table 1a. Selected Genes Differentially Expressed in High-Grade Astrocytomas (Genes up-regulated in gliomas) | Custom cDNA Array | 18395814 | Y Yang | Mol Carcinog | 2008 | An identification of stem cell-resembling gene expression profiles in high-grade astrocytomas. |
| 18395814-Table1b | Human | Table 1b. Selected Genes Differentially Expressed in High-Grade Astrocytomas (Genes down-regulated in gliomas) | Custom cDNA Array | 18395814 | Y Yang | Mol Carcinog | 2008 | An identification of stem cell-resembling gene expression profiles in high-grade astrocytomas. |
| 18397756-TableS3a | Mouse | TableS3a: F-N^int lowest (genes with a >1.75-fold difference in expression between populations F-N^int and F-N^lo, focusing on those in which F-N^int or - N^lo expression was the highest or lowest of the four populations examined) | affy Mouse 430 2 | 18397756 | JS Haug | Cell Stem Cell | 2008 | N-cadherin expression level distinguishes reserved versus primed states of hematopoietic stem cells. |
| 18397756-TableS3b | Mouse | TableS3b: F-N^int highest (genes with a >1.75-fold difference in expression between populations F-N^int and F-N^lo, focusing on those in which F-N^int or - N^lo expression was the highest or lowest of the four populations examined) | affy Mouse 430 2 | 18397756 | JS Haug | Cell Stem Cell | 2008 | N-cadherin expression level distinguishes reserved versus primed states of hematopoietic stem cells. |
| 18397756-TableS3c | Mouse | TableS3c: F-N^lo lowest (genes with a >1.75-fold difference in expression between populations F-N^int and F-N^lo, focusing on those in which F-N^int or - N^lo expression was the highest or lowest of the four populations examined) | affy Mouse 430 2 | 18397756 | JS Haug | Cell Stem Cell | 2008 | N-cadherin expression level distinguishes reserved versus primed states of hematopoietic stem cells. |
| 18397756-TableS3d | Mouse | TableS3d: F-N^lo highest (genes with a >1.75-fold difference in expression between populations F-N^int and F-N^lo, focusing on those in which F-N^int or - N^lo expression was the highest or lowest of the four populations examined) | affy Mouse 430 2 | 18397756 | JS Haug | Cell Stem Cell | 2008 | N-cadherin expression level distinguishes reserved versus primed states of hematopoietic stem cells. |
| 18405367-Table2a | Human | Table 2a: List of genes detected by microarray analysis and modification of their expression after neurogenic differentiation (undifferentiated versus differentiated MSC) | affy U133 Plus2 | 18405367 | T Tondreau | BMC Genomics | 2008 | Gene expression pattern of functional neuronal cells derived from human bone marrow mesenchymal stromal cells. |
| 18405367-Table2b | Human | Table 2b: List of genes detected by microarray analysis and modification of their expression after neurogenic differentiation (undifferentiated versus differentiated MSC) | affy U133 Plus2 | 18405367 | T Tondreau | BMC Genomics | 2008 | Gene expression pattern of functional neuronal cells derived from human bone marrow mesenchymal stromal cells. |
| 18510698-Table1 | Human | Table 1 Top 50 most highly upregulated genes (Specific upregulated genes profiles in CD41+ cells) | affy U133 Plus2 | 18510698 | CK Lim | Eur J Haematol | 2008 | Study of gene expression profile during cord blood-associated megakaryopoiesis. |
| 18510698-Table2 | Human | Table 2 Top 25 most highly downregulated genes (negatively regulates erythropoiesis, which share identical upstream regulatory pathways with megakaryopoiesis) | affy U133 Plus2 | 18510698 | CK Lim | Eur J Haematol | 2008 | Study of gene expression profile during cord blood-associated megakaryopoiesis. |
| 18510698-Table3 | Human | Table 3 Genes associated with cell adhesion | affy U133 Plus2 | 18510698 | CK Lim | Eur J Haematol | 2008 | Study of gene expression profile during cord blood-associated megakaryopoiesis. |
| 18510698-Table4 | Human | Table 4 Genes associated with lipid biosynthesis | affy U133 Plus2 | 18510698 | CK Lim | Eur J Haematol | 2008 | Study of gene expression profile during cord blood-associated megakaryopoiesis. |
| 18510698-Table5 | Human | Table 5 Genes associated with transcription regulator activity | affy U133 Plus2 | 18510698 | CK Lim | Eur J Haematol | 2008 | Study of gene expression profile during cord blood-associated megakaryopoiesis. |
| 18565887-TableA10 | Human | Table A10. Cluster G25 Seven gene clusters were most influential for explaining survival in patients assigned to TMZ/RT->TMZ. G25, dominated by EGFR probe sets (P .002; HR 2.8; 95% CI, 1.4 to 5.4; Table 1; Fig 6).] | affy U133 Plus2 | 18565887 | A Murat | J Clin Oncol | 2008 | Stem cell-related \self-renewal\" signature and high epidermal growth factor receptor expression associated with resistance to concomitant chemoradiotherapy in glioblastoma." |
| 18565887-TableA6 | Human | Table A6. Cluster G13 Seven gene clusters were most influential for explaining survival in patients assigned to TMZ/RT->TMZ | affy U133 Plus2 | 18565887 | A Murat | J Clin Oncol | 2008 | Stem cell-related \self-renewal\" signature and high epidermal growth factor receptor expression associated with resistance to concomitant chemoradiotherapy in glioblastoma." |
| 18565887-TableA8 | Human | Table A8. Cluster G18, associated with tumor resistance displayed some correlation with EGFR expression in particular with Aquaporin 4 (AQP4). | affy U133 Plus2 | 18565887 | A Murat | J Clin Oncol | 2008 | Stem cell-related \self-renewal\" signature and high epidermal growth factor receptor expression associated with resistance to concomitant chemoradiotherapy in glioblastoma." |
| 18614019-TableS4b | Mouse | Table S4b. Excel File Including All Genes Found to Be Significantly Up regulated and Down regulated (B >/= 0) by Expression Array Analysis in Tip60- or p400-Knockdown ESCs (XLS 2268 kb) (Down-regulated) | Agilent Mouse oligo arrays | 18614019 | TG Fazzio | Cell | 2008 | An RNAi screen of chromatin proteins identifies Tip60-p400 as a regulator of embryonic stem cell identity. |
| 19050837-Table1 | Mouse | Table 1 Lists of genes identified as expressed in common among SCDb, CD34-KSL, and SP Lin- libraries | Custom cDNA Array | 19050837 | Y Yashiro | Int J Hematol | 2009 | Transcriptional profiling of hematopoietic stem cells by high-throughput sequencing. |
| 19050837-Table2 | Mouse | Table 2 Lists of genes identified as shared only between CD34-KSL and SP Lin- libraries | Custom cDNA Array | 19050837 | Y Yashiro | Int J Hematol | 2009 | Transcriptional profiling of hematopoietic stem cells by high-throughput sequencing. |
| 19123479-TableS1 | Human | Supplementary Table 1. Differentially expressed genes in A549 and MCF7 cells treated with i6A or untreated (P<0.001). | Illumina Human v2 | 19123479 | F Colombo | Int J Cancer | 2009 | Pharmacogenomics and analogues of the antitumour agent N6-isopentenyladenosine. |
| 19129520-TableS26 | Human | Supplementary Table 26. Genes driving positive enrichment of hematopoietic stem/progenitor gene set in P9906 high-risk ALL. | affy U133A | 19129520 | CG Mullighan | N Engl J Med | 2009 | Deletion of IKZF1 and prognosis in acute lymphoblastic leukemia. |
| 19129520-TableS27 | Human | Supplementary Table 27. Genes driving negative enrichment of the B-cell signal transduction gene set in P9906 high-risk ALL. | affy U133A | 19129520 | CG Mullighan | N Engl J Med | 2009 | Deletion of IKZF1 and prognosis in acute lymphoblastic leukemia. |
| 19224407-Table1 | Human | Table 1 Highly expressed genes indentified in MSCs during differentiation | Custom cDNA Array | 19224407 | D Menicanin | Stem Cell Rev | 2009 | Genomic profiling of mesenchymal stem cells. |
| 19224407-Table3 | Human | Table 3 Commonly highly expressed genes in bone marrow, adipose tissue and umbilical cord derived MSCs populations compared to fibroblast cell populations | Custom cDNA Array | 19224407 | D Menicanin | Stem Cell Rev | 2009 | Genomic profiling of mesenchymal stem cells. |
| 19224407-Table4 | Human | Table 4 Genes highly expressed (> 8 fold) by MSCs derived from the bone marrow, amniotic fluid, amniotic membrane and cord blood in comparison to fetal organs | Custom cDNA Array | 19224407 | D Menicanin | Stem Cell Rev | 2009 | Genomic profiling of mesenchymal stem cells. |
| 19269367-TableS1 | Mouse | Table S1: Intestinal Stem Cell Transcriptome | Agilent Mouse oligo arrays | 19269367 | LG van der Flier | Cell | 2009 | Transcription factor achaete scute-like 2 controls intestinal stem cell fate. |
| 19269367-TableS2 | Mouse | Table S2: Intestinal Stem Cell Transcriptome regulated by Ascl2 | Agilent Mouse oligo arrays | 19269367 | LG van der Flier | Cell | 2009 | Transcription factor achaete scute-like 2 controls intestinal stem cell fate. |
| 19395651-Table1 | Mouse | Table 1. Genes Selectively Up-Regulated by Cav-1 (P132L) that Are Associated with Stem Cells, Invasiveness, and Metastasis | affy Mouse 430 2 | 19395651 | G Bonuccelli | Am J Pathol | 2009 | Caveolin-1 (P132L), a common breast cancer mutation, confers mammary cell invasiveness and defines a novel stem cell/metastasis-associated gene signature. |
| 19395651-TableS1a | Mouse | Supplemental Table 1a: 120 gene probe sets (representing 111 transcripts) that are differentially regulated between Cav-1 P123L transduced Met-1 cells compared to vector alone control. Contrast plotted Cav-1 P132L minus vector. Genes with +ve are up regulated by Cav-1 P132L. | affy Mouse 430 2 | 19395651 | G Bonuccelli | Am J Pathol | 2009 | Caveolin-1 (P132L), a common breast cancer mutation, confers mammary cell invasiveness and defines a novel stem cell/metastasis-associated gene signature. |
| 19395651-TableS1b | Mouse | Supplemental Table 1b: 120 gene probe sets (representing 111 transcripts) that are differentially regulated between Cav-1 P123L transduced Met-1 cells compared to vector alone control. Contrast plotted Cav-1 P132L minus vector. Genes with +ve are up regulated by Cav-1 P132L. | affy Mouse 430 2 | 19395651 | G Bonuccelli | Am J Pathol | 2009 | Caveolin-1 (P132L), a common breast cancer mutation, confers mammary cell invasiveness and defines a novel stem cell/metastasis-associated gene signature. |
| 19395651-TableS2a | Mouse | Supplemental Table 2a: Genes differentially Up regulated by Cav-1 (WT) and Cav-1 (P132L), compared with the vector alone control (pBABE). | affy Mouse 430 2 | 19395651 | G Bonuccelli | Am J Pathol | 2009 | Caveolin-1 (P132L), a common breast cancer mutation, confers mammary cell invasiveness and defines a novel stem cell/metastasis-associated gene signature. |
| 19395651-TableS2b | Mouse | Supplemental Table 2b: Genes differentially Up regulated by Cav-1 (WT) and Cav-1 (P132L), compared with the vector alone control (pBABE). | affy Mouse 430 2 | 19395651 | G Bonuccelli | Am J Pathol | 2009 | Caveolin-1 (P132L), a common breast cancer mutation, confers mammary cell invasiveness and defines a novel stem cell/metastasis-associated gene signature. |
| 19395651-TableS3a | Mouse | Supplemental Table 3a: Genes differentially Down regulated by Cav-1 (WT) and Cav-1 (P132L), compared with the vector alone control (pBABE). | affy Mouse 430 2 | 19395651 | G Bonuccelli | Am J Pathol | 2009 | Caveolin-1 (P132L), a common breast cancer mutation, confers mammary cell invasiveness and defines a novel stem cell/metastasis-associated gene signature. |
| 19395651-TableS3b | Mouse | Supplemental Table 3b: Genes differentially Down regulated by Cav-1 (WT) and Cav-1 (P132L), compared with the vector alone control (pBABE). | affy Mouse 430 2 | 19395651 | G Bonuccelli | Am J Pathol | 2009 | Caveolin-1 (P132L), a common breast cancer mutation, confers mammary cell invasiveness and defines a novel stem cell/metastasis-associated gene signature. |

**Table S2**: Genes used to generate the stemness bipartition and their corresponding weights when predicting the bipartition in other datasets, as well as pooled *t*-statistics for differential expression between the stem-like and differentiated subtypes. Negative weights correspond to the differentiated subtype and positive weights correspond to the stem-like subtype.

| **Ensembl Gene ID** | **Gene symbol** | **Weight** | **t-statistic** | **Increased Expression** |
| --- | --- | --- | --- | --- |
| ENSG00000183196 | CHST6 | -1.35308 | -4.98162 | Differentiated |
| ENSG00000104218 | CSPP1 | -2.30251 | -4.8431 | Differentiated |
| ENSG00000125798 | FOXA2 | -1.79568 | -4.79559 | Differentiated |
| ENSG00000185015 | CA13 | -2.62845 | -4.66143 | Differentiated |
| ENSG00000124749 | COL21A1 | -1.15273 | -4.53737 | Differentiated |
| ENSG00000126217 | MCF2L | -5.05696 | -4.49472 | Differentiated |
| ENSG00000056736 | IL17RB | -1.34383 | -4.4421 | Differentiated |
| ENSG00000165475 | CRYL1 | -1.73554 | -4.43554 | Differentiated |
| ENSG00000103460 | TOX3 | -0.64459 | -4.16476 | Differentiated |
| ENSG00000111725 | PRKAB1 | -1.78668 | -4.13937 | Differentiated |
| ENSG00000173812 | EIF1 | -2.45266 | -4.13523 | Differentiated |
| ENSG00000129595 | EPB41L4A | -1.98767 | -4.1162 | Differentiated |
| ENSG00000078900 | TP73 | -5.16763 | -4.11592 | Differentiated |
| ENSG00000108511 | HOXB6 | -0.5358 | -4.04601 | Differentiated |
| ENSG00000166352 | C11orf74 | -1.87056 | -4.03016 | Differentiated |
| ENSG00000172915 | NBEA | -1.09438 | -3.99161 | Differentiated |
| ENSG00000170703 | TTLL6 | -3.98166 | -3.95052 | Differentiated |
| ENSG00000129422 | MTUS1 | -1.25017 | -3.9413 | Differentiated |
| ENSG00000141469 | SLC14A1 | -1.04643 | -3.93555 | Differentiated |
| ENSG00000095397 | DFNB31 | -2.46696 | -3.93448 | Differentiated |
| ENSG00000153721 | CNKSR3 | -1.09457 | -3.90031 | Differentiated |
| ENSG00000120306 | C5orf32 | -1.51111 | -3.89289 | Differentiated |
| ENSG00000131437 | KIF3A | -1.5666 | -3.89175 | Differentiated |
| ENSG00000106688 | SLC1A1 | -1.25224 | -3.88831 | Differentiated |
| ENSG00000106236 | NPTX2 | -0.51116 | -3.81086 | Differentiated |
| ENSG00000056972 | TRAF3IP2 | -2.03394 | -3.80513 | Differentiated |
| ENSG00000197375 | SLC22A5 | -1.24915 | -3.60841 | Differentiated |
| ENSG00000227507 | LTB | 0.899348 | 3.57393 | Stem-like |
| ENSG00000134028 | ADAMDEC1 | 0.485284 | 3.63961 | Stem-like |
| ENSG00000125144 | MT1G | 0.459218 | 3.67258 | Stem-like |
| ENSG00000126787 | DLGAP5 | 0.751386 | 3.71182 | Stem-like |
| ENSG00000160949 | NFKBIL2 | 2.790947 | 3.74424 | Stem-like |
| ENSG00000133216 | EPHB2 | 1.933228 | 3.74559 | Stem-like |
| ENSG00000204304 | PBX2 | 1.295178 | 3.78859 | Stem-like |
| ENSG00000137692 | DCUN1D5 | 1.547644 | 3.8576 | Stem-like |
| ENSG00000169245 | CXCL10 | 0.447729 | 3.89099 | Stem-like |
| ENSG00000165655 | ZNF503 | 2.651613 | 3.93414 | Stem-like |
| ENSG00000135069 | PSAT1 | 0.950256 | 3.99408 | Stem-like |
| ENSG00000181444 | ZNF467 | 2.214061 | 4.01337 | Stem-like |
| ENSG00000106484 | MEST | 0.871049 | 4.08704 | Stem-like |
| ENSG00000185189 | NRBP2 | 1.132845 | 4.12654 | Stem-like |
| ENSG00000109472 | CPE | 0.907217 | 4.14908 | Stem-like |
| ENSG00000148737 | TCF7L2 | 1.652112 | 4.15423 | Stem-like |
| ENSG00000111247 | RAD51AP1 | 1.051026 | 4.29077 | Stem-like |
| ENSG00000153767 | GTF2E1 | 2.073054 | 4.3432 | Stem-like |
| ENSG00000198467 | TPM2 | 1.358715 | 4.55816 | Stem-like |
| ENSG00000079308 | TNS1 | 2.322022 | 4.6188 | Stem-like |
| ENSG00000171700 | RGS19 | 2.633212 | 4.66528 | Stem-like |
| ENSG00000121966 | CXCR4 | 1.111057 | 4.92512 | Stem-like |
| ENSG00000148841 | ITPRIP | 2.008421 | 4.93211 | Stem-like |
| ENSG00000198382 | UVRAG | 1.67292 | 5.10815 | Stem-like |

**Table S3**: The 13 gene sets used by Ben-Porath et al. to characterize classes of cancer as stem cell-like or differentiated. Genes in these gene sets were limited to those that were in the DNA microarray platform (Affy U133 Plus 2.0 platform) of the AOCS data [[1](#_ENREF_1)]. GeneSets are available in gmt and gmx file format on <http://bcb.dfci.harvard.edu/~aedin/publications/>

| Gene Set Name | Length (gene symbols) |
| --- | --- |
| ES.exp1 | 379 |
| ES.exp2 | 40 |
| Nanog.targets | 988 |
| Oct4.targets | 290 |
| Sox2.targets | 734 |
| NOS.targets | 179 |
| NOS.TFs | 37 |
| Suz12.targets | 1040 |
| Eed.targets | 1066 |
| H3K27.bound | 1118 |
| PRC2.targets | 654 |
| Myc.targets1 | 230 |
| Myc.targets2 | 775 |

ES.exp1 ACTA1,ACTC1,ACTN3,ADD2,PARP1,ALPL,AMD1,BIRC5,ATP1A2,BMPR1A,BUB1,BUB1B,C1QBP,CASP3,CBS,CCNA2,CCNB1,CD24,CDC2,CDC6,CDC20,CDC25A,CTSC,CHEK1,CRABP1,CRABP2,CRMP1,CSE1L,CXADR,CYP26A1,COCH,DHFR,DIAPH2,DLG3,DNA2L,DNMT3A,DNMT3B,DSG2,ECT2,SLC29A1,EPHA1,EPRS,ERBB2,ETV1,ETV4,FABP5,FEN1,GPC4,FGF2,FGF13,FGFR1,FKBP5,FOXO1,GABRA5,GABRB3,GAD1,GART,GJA1,GLDC,GPM6B,GPR19,MSH6,HAS3,HELLS,HMGB3,HMGA1,HMMR,HNRPAB,HSPA4,HSPA8,HSPD1,ILF3,INDO,ITPR3,JARID2,KAL1,KCNS3,KIF5C,KLKB1,KPNA2,KRT8,LCK,LGALS8,TACSTD1,M6PR,MAN2A1,MARS,MAT2A,MCM2,MCM3,MCM4,MCM5,MCM6,MCM7,MFGE8,MGST1,MICB,MRE11A,MSH2,NUDT1,MTHFD1,NASP,NFYB,NODAL,NPM1,NTHL1,NTS,OAZ2,ORC1L,PAK1,PCDH1,PDCD2,PDK1,PFAS,PIK3CB,PLCB3,PMAIP1,EXOSC9,PNN,PODXL,POLE2,POU5F1,PPM1B,PPP2R1B,PPP2R2B,PRIM1,PRIM2,PRKX,PRPS1,PTPN2,PTPRZ1,RAB3B,RARRES2,RBBP8,RFC3,RFC4,ABCE1,ROBO1,RPS24,RRM2,SALL2,SALL1,SCNN1A,SFRP1,SFRP2,SFRS1,SFRS7,ST6GAL1,SLC6A8,SLC16A1,SMS,SNRPA,SNRPN,SORL1,SOX2,SSB,TDGF1,TEAD4,TERF1,TFAM,TIA1,TMPO,TNNT1,UGP2,UNG,VSNL1,ZIC2,ZIC3,ZNF195,LRP8,FZD5,DEK,FXR1,USP9X,FZD7,UTF1,IFITM1,TMEFF1,RUVBL1,PPAP2A,USO1,ADAM23,TRIM24,HESX1,PROM1,FUBP1,DDX18,MAP7,CLDN6,SYNGR3,BUB3,DDX21,DCLK1,AURKB,NOLC1,PTTG1,MED14,CER1,HOMER1,C1orf38,GDF3,NFE2L3,DLG7,G3BP2,RABGAP1L,SRA1,CHAF1A,DNAJB6,AP1M2,G3BP1,GPR64,CEBPZ,AASS,PRMT3,ZNF267,TRIM22,NPM3,TUBB2C,LYPLA1,OLFM1,MAD2L2,NOL5A,PAICS,POLR3G,RAD51AP1,LEFTY1,IGF2BP3,IGF2BP2,MTHFD2,NMU,KIF2C,PIM2,NUDT21,LECT1,DIDO1,MYST2,HRASLS3,PSIP1,WDHD1,CHEK2,GPR176,OIP5,RRAS2,MTF2,SEPHS1,GARNL4,TTLL12,PASK,MDN1,COBL,BOP1,NCAPH,FRAT2,SIRT1,CBX5,TNPO3,PRKD3,KIF4A,RAD54B,NOL11,SFRS18,LRIG1,CNTNAP2,AUTS2,SERBP1,PITPNC1,GNL3,FOXD3,ITGB1BP3,C6orf66,CYP2S1,PYCR2,RRP15,GMNN,GAL,FAM108B1,PIPOX,ZNF589,RNF138,HSPC111,LARP7,ESF1,AZIN1,LSR,GINS2,GPRC5B,CECR1,BRWD1,C21orf45,FAM64A,PUS7,EPB41L4B,L1TD1,ERCC6L,RBM35A,NCAPG2,ZNF770,PAK1IP1,C12orf48,FANCL,DPPA4,C14orf115,NUDT15,BXDC2,C14orf106,LGR4,MCM10,PRPF40A,TMEM48,C12orf11,CCAR1,WDR12,RCC2,KLHL7,CHST7,EXOSC5,NUP107,SALL4,SLC39A10,MRS2L,SPC25,NLN,NLGN4X,MTA3,ZNF398,SEMA6A,LRRN1,CACHD1,FAM60A,PRDM14,NOC3L,ISG20L1,SLC13A3,CAPRIN2,DBNDD1,CAMKV,NUP37,ELOVL6,DCC1,GNPTAB,C1orf108,NARG2,LIN28,NANOG,PHF17,NARG1,MYO19,PUS1,TMEM177,SLC38A1,TXNDC1,WBSCR16,CDT1,SPRY4,TCF7L1,BCL2L12,USP44,GINS4,HPS3,RBM13,SLC7A3,ZSCAN10,ANGEL2,KIFC2,LOC91431,C20orf72,DMKN,EGLN3,CDCA5,MAL2,FAM46B,SCGB3A2,GYLTL1B,LOC157627,C8orf42,C11orf82,ARL5B,TUBB2B,CKMT1A

ES.exp2 CDC25A,CYP26A1,DNMT3A,DTYMK,EPHA1,ETV4,GPR19,GPR23,HELLS,MYBL2,ORC1L,ORC2L,POU5F1,PWP2,RBM4,TDGF1,ZIC3,HESX1,SLC5A6,CLDN6,RRP9,GDF3,GJA7,CHEK2,WSCD1,NCAPH,PRKD3,GOLGA7,L1TD1,ERCC6L,C14orf115,MCM10,CXorf15,PRDM14,ISG20L1,DBNDD1,DCC1,LIN28,ABHD9,NANOG

Nanog.targets ABCB7,ACADM,ACAT2,ACO2,ADAR,ADD3,ADFP,ADRBK2,AP2A1,AP1G1,AK3L1,ALCAM,ANGPT1,ANXA1,APLP2,APOA2,APOB,AQP2,ARF3,ARF4,ARHGAP1,ASAH1,ATF4,ATP5F1,ATP6V1A,B2M,BCAT1,BCKDHA,BCL9,BMP2,BMP7,POLR3D,BNIP1,ZFP36L1,KLF5,BUB1B,CA2,CA4,CACNA1A,CACNA2D1,CALB1,CALD1,CALM2,CALR,CAPZA2,CASP9,CAV1,RUNX1T1,SERPINH1,ENTPD1,CDC2,CDH1,CDH2,CDK6,CETN3,CFL1,CHD2,FOXN3,CKS2,CLIC1,CLN3,CNN2,CNN3,COL4A5,COL4A6,COL7A1,COL12A1,COX6A1,CPT1A,CRYZ,CS,MAPK14,CSNK1E,VCAN,CSTF1,CSTF3,CTGF,CTSL2,CXADR,CYLD,CYP1B1,DCX,DDX5,DMXL1,DHCR7,DNM2,DPAGT1,DPYSL2,DPYSL3,DTNA,DUSP6,DVL2,E2F3,EEF2,EIF4B,EIF4G2,ENSA,EPHA1,ERBB2,ERCC1,ERH,EXTL2,FANCA,ACSL4,FANCF,FBLN1,FARSA,FAT,FDPS,GPC4,FGF2,FGFR1,FGFR2,FHIT,FKBP1B,FOXO1,FOXO3,FUS,FZD2,GALK2,GANC,GART,GATA6,GJA1,GLA,GLG1,GLUD1,GNAI1,GNG10,GOLGA4,GSK3A,GSTT2,GTF2E1,GTF2H2,GYPC,H2AFX,H2AFZ,HIST1H2BD,HAS2,HELLS,HHEX,HNRNPA1,HNRNPA2B1,HNRPH2,HOXB5,HSPA4,HSPA5,TNC,ID1,ID2,IDH3A,IFI16,IGFBP2,IGFBP3,CYR61,ILF2,ILF3,ING1,INPP4A,ITGB1,JARID2,JUN,JUP,KCNN2,KDR,KPNA3,TNPO1,KRT18,LAMA4,LOH11CR2A,LRP2,LRP3,NBR1,SMAD3,MAN2C1,MCC,MDH1,MEF2A,MICA,MICB,MLH1,MAP3K11,MMP2,MMP9,MOBP,MOV10,MRE11A,MSH2,MTM1,NAP1L2,NDUFB3,NDUFS2,NKTR,NMT1,NODAL,NP,NPAS2,ROR1,NVL,OAZ2,ORC1L,OXA1L,P4HA1,PDCL,PBX1,PCBP1,PCNA,PCTK1,PCTK2,PDHB,ENPP2,PEX1,PFTK1,SERPINA1,PIK3R2,PITX2,EXOSC9,PODXL,POU2F1,POU4F1,POU5F1,PPP1R2,PPP2R1A,PPP2R1B,PPP2R3A,PPP2R5C,PRCP,PKIB,MAPK8,PRNP,PRSS8,PSEN2,PSMA1,PSMB1,PSMB4,PSMB5,PSMC2,PSMD9,PTN,PTPN1,PTPN2,PXMP3,QARS,RAB5A,RAB5B,RAD23A,RANGAP1,RAP1A,RARB,RASA1,RASGRF2,RBBP4,RBM4,RBP1,REST,RFX1,RGS10,RNF2,RPL17,RPL21,RPL32,RPLP1,RPS3A,RPS13,RPS18,RPS26,RPS27A,RYR3,S100A11,VPS52,SALL2,SALL1,SC5DL,SCNN1A,SDC4,SEPP1,SET,SFRP1,SFRP2,SFRS4,SGK,SCG5,ST3GAL2,SILV,SKIL,SLC1A1,HLTF,SNRP70,SNRPA,SNRPE,SNRPN,SNX1,SON,SOX2,SP2,SPARC,STAT3,STC1,AURKA,STRN,STXBP2,SUPT4H1,TAF4B,TAF12,TAL1,TALDO1,TARBP2,TBCC,TBL1X,TBP,TCF7L2,TCF20,TDGF1,TERF1,LEFTY2,THBS2,TIA1,TIAL1,TIAM1,TIMP4,TLE2,TLE3,TSPAN6,TOP2A,TSSC1,TTF1,TUBG1,TXNRD1,UBC,UBE2D3,SUMO1,UBP1,UFD1L,VIM,VLDLR,VRK2,WARS,WEE1,ZIC1,ZIC2,ZIC3,CNBP,ZNF174,ZNF185,ZNF202,ZNF217,ZNF226,ZNF228,MAP3K12,USP7,BAT3,MYST3,MLLT10,FXR1,ANP32A,SLC7A5,MKKS,PICALM,AXIN2,CDC7,CDC45L,EOMES,FZD7,FZD8,HIST2H2AA3,HIST1H4C,TTF2,PPFIBP1,PARG,AP3B1,CDC14B,KHSRP,KLF7,PPAP2A,EIF3D,EIF3F,PEA15,B3GALT4,GBF1,RIPK1,CDS2,MTMR1,MPDZ,TRIM24,DPM1,HESX1,ALKBH1,TSC22D1,CDC123,CDC16,DDX18,AP1S2,SPAG9,NFS1,PAPSS2,CLDN6,PKMYT1,USP10,SEC22C,SDCCAG1,TMSB10,BUB3,LARGE,VAPB,NOLC1,LRAT,PNMA1,MSC,DHRS3,TRIP10,TRIP4,GTF3C4,B4GALT6,CNOT8,CER1,DDX23,CDYL,MED23,EIF2AK3,PIGL,EEF1E1,TMEM59,BAG5,ATP6V1G1,SEC22B,GTPBP1,GDF3,PREPL,RBM39,NFE2L3,NCOR1,FEZ1,MORF4L2,ZNF516,KIAA0391,FAM131B,TMEM63A,SART3,KNTC1,FAM115A,KIAA0101,BCLAF1,KIAA0652,DHX38,DLG7,SFI1,TSC22D2,ARHGAP11A,LCMT2,ZEB2,EPM2AIP1,SUPT7L,NCAPD2,JOSD1,USP3,MED12,NAALAD2,ACOT8,ABCF2,SNUPN,GPC6,ACTR1B,ACTR1A,SFRS14,CEBPZ,CHST4,RBM7,DDX39,PSMD14,SPRY1,KATNB1,TRIM22,HMG20A,CITED2,CEPT1,YAP1,TIMM23,RBM14,HMGN4,SEMA4F,SEMA3C,DDX17,NEBL,ANP32B,CCT7,SORBS1,AHSA1,USP16,POLR3G,TRIM16,PDPN,GAS2L1,LEFTY1,IGF2BP3,KHDRBS1,CUGBP2,GNA13,CCT6B,MGEA5,NFAT5,RAI1,SEC24A,UTP14A,C5orf4,NMU,BLCAP,TCERG1,MORF4L1,MSL3L1,SERINC3,TMED10,KDELR3,RABL4,RBPMS,PIM2,ABHD2,LECT1,DIDO1,HNRPUL1,KRR1,PWP1,IL1RAPL1,PKIG,NUDT5,WDHD1,WDR6,FZD10,DDX20,RNF24,DUSP12,COPE,XAB1,CBX3,OIP5,DNAJC8,ELL2,DKK1,SLC4A1AP,TPX2,NT5C2,FBXL11,WDTC1,KIAA0241,EXPH5,SPG20,PHF8,39331,ANKRD15,RRS1,SULF1,DNAJC9,KLHL18,FBXW11,EHBP1,SIN3B,NEDD4L,SASH1,DNAJC16,OBSL1,SMG5,KIAA0368,FRAT2,COTL1,COMMD3,SLC44A1,SF3B1,ICMT,CBX5,ISCU,LEPROTL1,HEY2,KCTD2,R3HDM1,ZNF281,RBM9,CDC42EP4,ORC6L,MKRN1,SSBP2,SSBP3,SLC7A11,LSM5,SGK3,RAB38,GSPT2,C9orf5,RAB3GAP2,RAD54B,BAMBI,ARIH1,METTL7A,WDSOF1,POLR1A,ZNF473,RP11-529I10.4,ZNF521,CLIC4,C20orf194,SFRS18,TMEM87A,TBC1D10B,GORASP2,PLEKHG3,PPP1R16B,GGA1,PRPF31,TRPC4AP,KIF26A,PHGDH,BSCL2,HBP1,ZRF1,ANKRD1,STK36,SALL3,SERP1,LSM3,APEX2,TNRC6A,GOLIM4,UBE2S,PRPF19,POLL,MAT2B,BZW2,MCTS1,ATAD2,C16orf72,UBE2T,HSPC171,TBK1,CYP2S1,UCRC,TFPT,NME7,LMCD1,SLC40A1,EHD4,MYEF2,AK3,F11R,C14orf122,C1orf121,NDUFA13,KLHL5,IFT52,C8orf70,APH1A,HSD17B12,ING4,HN1,SS18L2,NUSAP1,VRK3,C3orf19,MRPL37,CXorf26,MRPL27,PIPOX,ARMCX1,TNFRSF12A,UBR5,NIP7,DDX41,HSPC111,LARS,UFM1,MIR16,LARP7,ASB1,TMEM66,MPP6,SUFU,UIMC1,WBP11,NUP54,FXYD5,MYO3A,PPIL3,ZFAND6,CCDC93,FAM35A,DDX49,FAM63B,RRN3,DYM,C10orf26,LRRC49,ANKRD49,TMEM103,WHSC1L1,RPP25,ZNF434,TMEM160,HCFC1R1,ZNF770,PIH1D1,PPP2R3C,PRPF39,PTCD3,RNF31,FANCL,C6orf166,THAP1,DARS2,RIF1,RIC8B,NADSYN1,C12orf35,P15RS,DPPA4,EXDL2,C14orf115,UBE2W,RNF121,SLC39A9,ZNF331,C2orf56,ETNK1,FEM1A,OTUB1,C20orf42,OSGEP,IWS1,RBM22,CCDC94,VPS35,FLJ10769,ZNF701,H2AFJ,PRR11,FOXJ2,JMJD1A,CAND1,UBAP2,WWC3,C3orf10,CISD1,PSENEN,APOM,LIN37,GNG12,KLHL4,NDNL2,KIAA1217,EIF4ENIF1,C21orf59,GRIPAP1,C15orf24,UBQLN4,EXOSC5,SMARCAD1,GPR108,FEM1C,OLFML3,XAB2,OTUD7B,FAM20C,KIF15,ANKMY2,RAB25,INTS12,PHTF2,ZNF286A,KIAA1143,SFRS15,ODF2L,ARID1B,ZNF398,SLAIN2,RANBP10,LRRN1,CACHD1,TMEM16H,NOPE,GATAD1,SCAF1,EPS15L1,C6orf115,SENP2,EXOC4,SAV1,NIF3L1,C14orf133,NSUN3,PRDM14,PERP,LHPP,KIF9,TFB2M,RAB17,NOC3L,RNF25,DCLRE1C,TTC31,NUCKS1,C11orf1,39148,NOL6,TMEM135,RSRC2,PLEKHA3,RASL11B,TMEM108,SCNM1,GIYD2,C19orf58,KCTD15,SECISBP2,TMEM109,DCC1,WDR77,C19orf42,PHF23,GNPTAB,TMEM43,LRFN3,CARS2,C13orf7,C14orf138,HMBOX1,C1orf54,C1orf108,HSPBAP1,PARP8,TMEM149,TBL1XR1,PALB2,ISOC2,C15orf29,FBXO31,ALG9,GSTCD,MOBKL2B,ASAM,METTL8,DCAKD,C9orf82,NANOG,CNTNAP3,DHDDS,PHF17,GRHL2,DNAJB14,NIP30,UXS1,FLJ22795,MUS81,C2orf44,WDR23,CYB5B,ECOP,C1orf21,TXNDC5,URM1,MAP1LC3B,MED25,C14orf156,SLC7A5P1,TCF7L1,SF3B5,ABHD11,EIF2A,USP44,TOMM40L,ARID5B,ASCC2,FAM96A,TRAF7,DCUN1D5,ALKBH7,CHCHD5,LSMD1,C14orf153,SPIRE2,DCTN5,GTPBP3,FKSG24,C14orf151,SFT2D3,ADO,ZSCAN10,LINGO1,DIRC2,ATG4C,PRPF38A,JUB,TMEM60,MST150,ZCCHC3,ZCRB1,RSPRY1,ATPBD4,ZNF551,CCDC45,TMEM55B,TCEAL8,C19orf6,C22orf32,CABLES1,WDR20,COG7,ZNF300,HIST3H2A,PERLD1,ARMC6,ATPIF1,EGLN3,PRKCDBP,SAT2,C6orf117,CCDC104,FAM54A,DTX2,STK11IP,OSBPL1A,TMEM123,TLCD1,LYPD1,SCGB3A2,SLC36A4,CYP2R1,LRIG3,NAT12,C16orf63,IQCK,FAM100A,TMEM170,HEXIM2,WDR81,C18orf37,TYW3,ZNF684,C1orf83,TMEM77,MBOAT2,OSR1,ZFP42,AASDH,GRPEL2,WDR36,FAM92A1,UNC5D,C20orf96,C20orf52,HECTD2,ZNF664,C12orf60,FBXL14,C1orf211,SLC30A7,C1orf213,COMMD7,CCDC12,C9orf19,MARVELD2,IRX2,AMOTL1,C8orf42,SPRED1,ADAL,FAM134C,ASXL1,HIGD2A,THAP8,FLJ25801,C11orf82,FAM124A,ZNRF2,SGMS1,BCL9L,GLT8D3,FAM100B,ZIK1,C19orf54,WDR62,RABL3,ATP11C,AMIGO2,FAM33A,UNQ501,RAB15,IMAA,FLJ45455,IER5L,PCNXL3,FAM128A,FAM72B

Oct4.targets ADD3,AUH,BMP7,ZFP36L1,KLF5,BUB1B,CA2,CA4,CACNA2D1,CAPZA2,ENTPD1,CDH1,COL12A1,CPT1A,CTGF,DPYSL2,DPYSL3,DTNA,DUSP6,EIF2S1,ELAVL2,EPHA1,FGF2,FGFR1,FGFR2,FOXO1,FUS,GAP43,GAS1,GATA6,GJA1,GPC3,GLDC,GNG10,GPS1,GRID2,HAS2,HHEX,HMGB2,HOXB5,TNC,ID2,IFI16,JARID2,JUND,JUP,KCNN2,KDR,LAMA4,MAN2C1,MCC,MEIS2,MAP3K3,MMP2,MTM1,NCBP1,NEFM,NEFL,ROR1,ORC1L,PAK1,PDCL,PCTK2,ENPP2,PFTK1,EXOSC9,POU5F1,PPP2R1B,PPP2R3A,PRPS1,PSMA3,PTPN2,RAB5A,RAD51C,RASGRF2,REST,RFNG,RPL32,RPS3A,RPS18,VPS52,SALL1,SET,SFRP1,SFRP2,SFRS4,SKIL,SNRPN,SOX2,STAT3,TAF12,TAL1,TALDO1,TCF4,TCF12,TCF20,TDGF1,NR2F2,LEFTY2,THBS2,TLE3,TOP2A,TRPS1,UBE2D3,WEE1,ZIC1,ZIC2,ZIC3,USP7,MYST3,MLLT10,CDC7,EOMES,HIST2H2BE,PARG,CDC14B,KLF7,PPAP2A,B3GALT4,TRIM24,HESX1,ALKBH1,TSC22D1,CDC123,SPAG9,BUB3,LARGE,LRAT,MSC,DHRS3,TRIP4,GTF3C4,PLAA,CDYL,BAG5,ATP6V1G1,GTPBP1,NFE2L3,FEZ1,KIAA0101,KIAA0174,ZEB2,SUPT7L,JOSD1,MED12,NAALAD2,ARPC5,ZMPSTE24,TRIM22,HMG20A,TIMM23,NEBL,SORBS1,POLR3G,MGEA5,BLCAP,GADD45G,MAGED2,STMN2,IL1RAPL1,HHLA3,HYPE,NUDT5,FZD10,RNF24,DUSP12,ZHX2,DKK1,SLC4A1AP,WDFY3,PHF8,ANKRD15,SULF1,DNAJC9,FBXW11,OBSL1,SMG5,PIP5K1C,FRAT2,COMMD3,SLC44A1,ICMT,CHST5,MKRN1,SSBP2,ZKSCAN5,CBY1,RAD54B,BAMBI,TXN2,ABTB2,SFRS18,PRPF31,KIAA1279,PHGDH,ANKRD1,SNX5,TJP3,TNRC6A,KCNMB4,MRPL13,ATAD2,TFPT,TRA2A,MYEF2,KLHL5,HN1,DBR1,CXorf26,PIPOX,UBR5,ATP6V1D,UFM1,ANKHD1,WDR70,RIF1,RIC8B,C12orf35,DPPA4,LSG1,TBC1D22B,NPLOC4,RBM22,CCDC94,FLJ10769,TMEM30A,H2AFJ,PRR11,JMJD1A,KLHL4,PARD3,SMARCAD1,OLFML3,KIF15,KIAA1143,ARID1B,LRRN1,TMEM16H,TGIF2,PRDM14,LHPP,SOX17,ZDHHC6,NUCKS1,DDX31,ARMCX5,ACD,GNPTAB,LRFN3,C13orf7,EFTUD1,PRKRIP1,TBL1XR1,C15orf29,NANOG,CNTNAP3,DHDDS,PHF17,GRHL2,URM1,C14orf156,TCF7L1,USP44,C14orf153,GTPBP3,C14orf151,LINGO1,PRPF38A,MST150,ATPBD4,CABLES1,WDR20,SFXN1,PRKCDBP,NAT12,TMEM170,OSR1,AASDH,WDR36,C1orf211,COMMD7,IRX2,RDH10,C9orf97,SPRED1,SGMS1,AMIGO2,FAM33A,IER5L,MED11

### Sox2.targets

ABCB7,ACO2,ACOX1,ADAR,ADD3,ADFP,ADRBK2,AP2A1,ALCAM,ALPL,APEX1,APP,AQP2,ARF3,ARF4,ARL4D,ARHGAP1,ASNA1,ATF3,ATF4,ALDH7A1,ATP5F1,AUH,BCAT1,BCKDHA,CCND1,BCL9,BMP2,BMP7,ZFP36L1,KLF5,BTG1,BUB1B,CA2,CA4,CACNA1A,CACNA2D1,CALM2,CALR,CAPZA2,CASP9,CDC2,CDH2,CDH3,CDK6,CETN3,CFL1,FOXN3,CLN3,COL12A1,COPB1,CPT1A,CSNK1E,VCAN,CTGF,CTH,DDX5,DNM2,DPAGT1,DPYSL2,DPYSL3,DTNA,DUSP6,DVL2,E2F3,EEF2,EGR3,EIF4G2,ELAVL2,ENSA,EPHA1,ERBB2,FANCC,FANCF,FARSA,FGF2,FGFR1,FGFR2,FOXO1,FTL,FUS,FZD2,GJA1,GNAI1,GNG10,GRK6,GRID2,GSK3A,GSK3B,H2AFX,H3F3B,HAS2,HDAC2,HELLS,HHEX,HMOX1,HNRNPA1,HNRNPA2B1,HNRNPC,HNRPK,HNRNPL,HSP90AB1,TNC,ID1,IDH3G,IFI16,IGFBP2,CYR61,IK,ILF2,ILF3,INHBA,JARID2,JUP,KCNN2,KDR,KIF11,KPNA3,LAMA4,LASP1,ABLIM1,LOH11CR2A,LOXL2,LRP2,CAPRIN1,NBR1,SMAD3,MAN2C1,MLH1,MAP3K11,MOBP,MTM1,MYO9A,NCBP1,NDUFA2,NDUFB5,NDUFB8,NIT1,NKTR,NOTCH1,NP,ROR1,OAZ2,ORC1L,OXA1L,PRDX1,PAK1,PDCL,PCBP1,PCSK5,PCTK1,PCTK2,ENPP2,PFTK1,SERPINA1,PIN4,PITX2,EXOSC9,POU2F1,POU5F1,PPM1B,PPP1R2,PPP1R10,PPP2R1A,PPP2R1B,PPP2R3A,PPP2R5C,PRCC,PRKAR1A,PRNP,PRPSAP1,PRSS8,PSEN2,PSMB1,PSMC2,PTN,PTPN2,PTPN3,RAB5A,RAD23A,RASA1,RASGRF2,REST,RFX1,RGS10,RPL7,RPL9,RPL15,RPL30,RPL36A,RPLP1,RPS3A,RPS18,RPS26,RPS29,RTN2,SORT1,VPS52,SALL1,SAT1,SC5DL,SCNN1A,CXCL5,SDHD,SET,SFPQ,SFRP1,SFRP2,SFRS4,SFRS7,SGTA,SH3GL3,ST3GAL2,SKIL,SLC3A2,HLTF,SNAPC1,SNAPC3,SNRPA,SNRPD3,SNRPN,SNX1,SOX2,SP2,SSR4,STAT3,STC1,STCH,STXBP2,TAF12,TAL1,TALDO1,TARBP2,TBP,TCF7L2,TCF20,PPP1R11,TDGF1,LEFTY2,THBS2,THOP1,TIA1,TIAL1,TIMP4,TLE1,TLE2,TLE3,TNFAIP2,TOP2A,TPM3,HSP90B1,TUBG1,TXNRD1,UBC,UBE2D3,UBP1,UFD1L,UGT8,VASP,VIM,ZIC1,ZIC2,ZIC3,ZNF140,ZNF217,MAP3K12,USP7,FZD3,MYST3,MLLT10,ANP32A,SLC7A5,TAF15,MKKS,CDC7,CDC45L,EOMES,FZD1,FZD7,HIST2H2AA3,HIST2H2BE,HIST1H4C,TTF2,TEAD2,OGT,CDC42BPA,PIK3R3,PARG,AP3B1,CDC14B,PPAP2A,USO1,EIF3F,B3GALT4,GBF1,RIPK1,TRIM24,SAP30,HESX1,TSC22D1,PER2,SPAG9,SDCCAG1,TMSB10,BUB3,DDX21,LARGE,LRAT,PNMA1,MSC,DHRS3,B4GALT6,CNOT8,CER1,CDYL,MED17,GGPS1,ATP6V1G1,RBM39,MPHOSPH1,NFE2L3,RNF14,FEZ1,MORF4L2,MDC1,ZNF516,KIAA0391,PUM1,TMEM63A,ZNF646,HDAC9,KNTC1,FAM115A,KIAA0247,BCLAF1,DHX38,MTSS1,SPCS2,ARHGAP11A,ZEB2,HEPH,EPM2AIP1,SMG7,MED12,NAALAD2,ACOT8,ABCF2,DPP3,HUWE1,GPC6,ACTR1A,G3BP1,SFRS14,DDX39,MRPS31,TRIM22,HMG20A,TIMM23,VAT1,DDX17,NEBL,ANP32B,ARFGEF1,POLR3G,TRIM16,PDPN,LEFTY1,EXOC5,IGF2BP3,GNA13,MGEA5,RBBP9,SDCCAG8,C5orf4,NMU,GADD45G,MAGED2,MORF4L1,MSL3L1,CLP1,MAPRE2,KIF2C,RBPMS,UBE2C,DIDO1,HNRPUL1,PWP1,HYPE,NUDT4,FZD10,RNF24,DUSP12,CBX3,OIP5,MTF2,LPHN1,DKK1,MAST1,ACIN1,AZI1,EXPH5,MRPS27,PHF8,NCDN,39331,ZCCHC14,ANKRD15,KIAA0280,RRS1,SULF1,NUP160,FBXW11,NEDD4L,SASH1,DNAJC16,OBSL1,KIAA0368,PIP5K1C,FRAT2,COMMD3,SLC44A1,ICMT,CBX5,ETHE1,ZNF281,ORC6L,PPP1R15A,SSBP3,SLC7A11,SGK3,GSPT2,C9orf5,CBY1,RAB3GAP2,RAD54B,LSM4,BAMBI,ARIH1,ABTB2,USP49,CLIC4,FAM98A,C20orf194,SFRS18,TBC1D10B,PLEKHG3,GGA1,KIAA1279,TRPC4AP,KIF26A,TIMM9,TIMM8B,HBP1,ZRF1,ANKRD1,FOXP1,TJP3,EIF2C2,SLC39A1,LSM3,TNRC6A,PRPF19,KCNMB4,MAT2B,NKIRAS1,MAPBPIP,C11orf67,MRPS18B,MCTS1,ATAD2,MRPL15,UBE2T,C16orf80,SCG3,USP25,UCRC,TRA2A,NME7,AK3,COPS7A,C14orf122,TXNDC12,KLHL5,APH1A,RDH11,HSD17B12,ING4,HN1,NUSAP1,CRIM1,PIPOX,MEX3C,UBR5,NIP7,C11orf73,UFM1,LARP7,PCF11,NAG,MRPS23,SUFU,CYB5R2,UIMC1,WBP11,ARID4B,CRKRS,RSF1,RNUXA,FXYD5,RAB4B,POLE3,ERRFI1,GDAP1,SLC38A2,CCDC93,FBXL19,FAM63B,RRN3,TRIT1,TMEM103,ANKHD1,FLJ20309,WHSC1L1,TMEM160,TIPIN,ZNF770,ZSCAN2,PIH1D1,PPP2R3C,C14orf119,RNF31,C6orf166,RBM23,DARS2,RIF1,RIC8B,C12orf35,DPPA4,C14orf115,UBE2W,LSG1,ZNF331,FEM1A,STAP2,OSGEP,HIF1AN,IWS1,RBM22,CCDC94,POLR3E,VPS35,FLJ10769,C14orf108,RCOR3,ZNF701,H2AFJ,PRR11,FOXJ2,JMJD1A,EAPP,CISD1,MYNN,KIAA1166,UBQLN4,BDH2,SPIRE1,EXOSC5,SMARCAD1,OLFML3,XAB2,TNFSF5IP1,KIF15,AVEN,RAB25,MRPL47,KIAA0495,ZNF286A,KIAA1143,SFRS15,ODF2L,ARID1B,NUFIP2,SEMA6A,ARRDC3,RANBP10,LRRN1,CACHD1,TMEM16H,SCAF1,FAM60A,SENP2,TGIF2,SAV1,NSUN3,ZNF335,PRDM14,LHPP,RAB17,NOC3L,MOSPD3,NUCKS1,MRPS11,NOL6,RSRC2,C1orf163,PLEKHA3,RASL11B,TMEM108,C19orf43,SCNM1,C19orf58,TMEM109,DCC1,WDR77,PHF23,GNPTAB,LRFN3,C13orf7,C14orf138,HMBOX1,C1orf54,C1orf108,PRKRIP1,TBL1XR1,TBC1D17,ZNF668,C15orf29,VASH2,DCAKD,NANOG,KIAA0319L,DHDDS,PHF17,GRHL2,RMI1,C14orf159,PIF1,MUS81,FBXO11,WDR23,C1orf21,URM1,FIP1L1,DIAPH3,MED25,SLC7A5P1,TCF7L1,SF3B5,ABHD11,C22orf13,USP44,ARID5B,FAM96A,POLDIP3,LSMD1,AKT1S1,PPAPDC1B,MRPL43,GTPBP3,HDGF2,LINGO1,CCDC123,ZFYVE19,PRPF38A,MPND,JUB,MST150,ZCCHC3,ZCRB1,ATPBD4,CCDC45,TMEM55B,BTF3L4,CABLES1,ZNF300,HIST3H2A,PERLD1,ARMC6,SFXN1,C21orf66,TSGA14,EGLN3,PRKCDBP,OSBPL1A,TMEM123,WDFY2,LYPD1,NAT12,TMEM170,HEXIM2,WDR81,C18orf37,ZNF428,NDUFA11,AASDH,WDR36,C20orf96,HECTD2,XRRA1,FAM76B,ZNF664,C12orf60,FBXL14,FLJ40125,CREB3L4,C1orf211,COMMD7,IRX2,AMOTL1,RDH10,FBXO16,C8orf42,C9orf97,SPRED1,FAM134C,C1orf55,UBR1,TUBB,FLJ25801,FAM124A,C6orf130,SGMS1,PGM2L1,GLT8D3,FAM100B,ZIK1,C19orf54,C1orf174,ZNF677,FAM33A,RAB15,IMAA,IER5L,PCNXL3

NOS.targets ADD3,BMP7,ZFP36L1,KLF5,BUB1B,CA2,CA4,CACNA2D1,CAPZA2,COL12A1,CPT1A,CTGF,DPYSL2,DPYSL3,DTNA,DUSP6,EPHA1,FGF2,FGFR1,FGFR2,FOXO1,FUS,GJA1,GNG10,HAS2,HHEX,TNC,IFI16,JARID2,JUP,KCNN2,KDR,LAMA4,MAN2C1,MTM1,ROR1,ORC1L,PDCL,PCTK2,ENPP2,PFTK1,EXOSC9,POU5F1,PPP2R1B,PPP2R3A,PTPN2,RAB5A,RASGRF2,REST,RPS3A,RPS18,VPS52,SALL1,SET,SFRP1,SFRP2,SFRS4,SKIL,SNRPN,SOX2,STAT3,TAF12,TAL1,TALDO1,TCF20,TDGF1,LEFTY2,THBS2,TLE3,TOP2A,UBE2D3,ZIC1,ZIC2,ZIC3,USP7,MYST3,MLLT10,CDC7,EOMES,PARG,CDC14B,PPAP2A,B3GALT4,TRIM24,HESX1,TSC22D1,SPAG9,BUB3,LARGE,LRAT,MSC,DHRS3,CDYL,ATP6V1G1,NFE2L3,FEZ1,ZEB2,MED12,NAALAD2,TRIM22,HMG20A,TIMM23,NEBL,POLR3G,MGEA5,FZD10,RNF24,DUSP12,DKK1,PHF8,ANKRD15,SULF1,FBXW11,OBSL1,FRAT2,COMMD3,SLC44A1,ICMT,RAD54B,BAMBI,SFRS18,ANKRD1,TNRC6A,ATAD2,KLHL5,HN1,PIPOX,UBR5,UFM1,RIF1,RIC8B,C12orf35,DPPA4,RBM22,CCDC94,FLJ10769,H2AFJ,PRR11,JMJD1A,SMARCAD1,OLFML3,KIF15,KIAA1143,ARID1B,LRRN1,TMEM16H,PRDM14,LHPP,NUCKS1,GNPTAB,LRFN3,C13orf7,TBL1XR1,C15orf29,NANOG,DHDDS,PHF17,GRHL2,FAM130A1,URM1,TCF7L1,USP44,GTPBP3,LINGO1,PRPF38A,MST150,ATPBD4,CABLES1,PRKCDBP,TMEM170,AASDH,WDR36,C1orf211,COMMD7,IRX2,SPRED1,SGMS1,FAM33A,IER5L

NOS.TFs ZFP36L1,KLF5,FOXO1,HHEX,IFI16,JARID2,POU5F1,REST,SALL1,SOX2,STAT3,TAF12,TAL1,TCF20,TLE3,ZIC1,ZIC2,ZIC3,MYST3,MLLT10,EOMES,HESX1,MSC,NFE2L3,ZEB2,MED12,HMG20A,POLR3G,PHF8,COMMD3,ARID1B,PRDM14,TBL1XR1,NANOG,PHF17,TCF7L1,IRX2,

Suz12.targets ACADL,ACCN1,ADARB2,ADCY8,ADCYAP1,ADM,ADRA1A,ADRA2A,ADRB1,ADRB3,ADRBK2,ACAN,NR0B1,ALOX5,ALOX15,ALX3,AQP5,ABCC6,RHOB,ARHGAP6,PHOX2A,ASCL1,ASCL2,ASTN1,ZFHX3,ATF3,ATOH1,BAI2,NKX3-2,BCL2,BHMT,BNC1,BMI1,BMP3,BMX,FOXL2,CA3,CACNA1A,CACNA1B,CACNA1D,CACNA1E,CACNB4,CALCA,CAV2,CBLN1,CBR3,CD8A,CD34,CD38,CD44,CD70,CDH6,CDH7,CDH11,CDH13,CDKN2C,CDX2,CDX4,CEBPD,CFTR,CHRNA3,CIDEA,CLCN5,CNTFR,COL2A1,COL4A5,COL4A6,COL9A2,COMP,CRABP1,CRHBP,CRHR1,CRIP1,CRMP1,CRYBA2,CSF1,CTNND2,CYP1B1,CYP2J2,CYP24A1,CYP26A1,CYP27B1,DACH1,DGKG,DCC,COCH,DIO3,DLX1,DLX2,DLX3,DLX4,DMRT1,DPP6,DRD5,DSC3,TSC22D3,DUSP4,DUSP6,E2F4,EDN3,EFNA1,EFNA3,EFNB1,EGR2,EGR3,EGR4,ELAVL3,ELF4,EMX1,EMX2,EN1,EN2,EPAS1,EPHA4,EPHA5,EPHB1,EPHB3,EPS8,ERBB4,ERG,ETFA,EYA2,F2R,FBN1,FBN2,FBP1,GPC4,FGF3,FGF5,FGF9,FGF13,GPC5,FOXG1,FOXF1,FOXL1,FOXE3,FOXJ1,FOXE1,FOXD2,FLI1,FLT3,AFF2,FUT4,FZD2,GABRA2,GABRA4,GAD2,B4GALNT1,GATA2,GATA3,GATA4,GATA6,GATM,GBX2,GDNF,GFI1,GFRA1,GFRA2,GHR,GHSR,GJB2,GPC3,GNAS,GPM6B,PRLHR,GPR12,GPR27,MLNR,GRIA2,GRID1,GRIK1,GRIK3,GRIN2B,GRIN2D,GRM7,CXCL1,GSC2,GSTM3,GUCY1A2,GUCY1A3,GUCY2D,HBA1,HBA2,NRG1,HHEX,MNX1,HLF,HLX,HMX2,FOXA1,FOXA2,ONECUT1,TLX1,TLX2,HOXB1,HOXB2,HOXB3,HOXB6,HOXB7,HOXB8,HOXC4,HOXC5,HOXC6,HOXC8,HOXC9,HOXC11,HOXC12,HOXC13,HOXD1,HOXD3,HOXD4,HOXD8,HOXD9,HOXD11,HOXD12,HOXD13,HPCAL1,HSF4,HSPA6,HTR1A,HTR2C,HTR6,HTR7,ICAM4,IGF2,IGFBP5,IHH,IL7,IL15RA,TNFRSF9,IMPDH1,INSM1,INSRR,PDX1,ISL1,ITGA4,ITPKA,ITPKB,JUN,KAL1,KCNA1,KCNA3,KCNA5,KCNC2,KCNC4,KCND3,KCNH1,KCNJ2,KCNJ3,KCNJ5,KCNJ6,KCNK2,KCNMA1,KCNQ3,LAMA3,LGALS3,LMX1B,LOX,LPL,LRP2,LTBP2,LTK,TACSTD2,MAB21L1,SMAD6,MAF,MAL,MAP6,MAPT,MDS1,CD99,MID1,MLLT3,FOXO4,CITED1,MSX1,MT1A,MT1B,MT1H,MT1JP,MT1M,MT1X,MTM1,MYF6,MYO5B,MYOD1,NBL1,NCAM1,NDP,NEFM,NEFH,NELL1,NEFL,NEUROD1,NEUROD2,NEUROG1,NFIA,NFIC,NFIX,NGFB,NGFR,NHS,NKX2-2,NKX3-1,NKX6-1,NOS1,NOTCH2,NPAS1,NPAS2,NPR3,NPTX1,NPY1R,NPY5R,NRCAM,NTRK1,NTRK2,NTRK3,OCA2,OPRD1,OPRK1,SIX6,OTX1,OTX2,OVOL1,PAPPA,PAX1,PAX2,PAX3,PAX5,PAX6,PAX7,PAX9,PCDH8,PCTK1,PDGFRA,SLC26A4,PENK,PGM5,PGR,PITX1,PITX2,PITX3,PKP1,PLEC1,PLS3,PLXNA2,PMP22,POLE,POMC,POU3F1,POU3F4,POU4F1,POU4F2,POU4F3,PRKCE,PRKCH,PRKG1,MAPK4,MAPK11,HTRA1,PYY,PTGDR,PTGER2,PTGER3,PTGER4,PTGFR,PTHLH,PXMP2,RARRES1,RASGRF1,RASGRF2,RBBP7,RBP4,RGS10,RORB,RXRG,RYR3,SCN4B,SCNN1G,SCP2,SCTR,SDC2,SECTM1,SFRP1,SFRP5,SHH,SHOX,SHOX2,ST3GAL1,ST8SIA1,SIM2,SIX1,SIX3,SLC1A2,SLC1A4,SLC5A5,SLC6A1,SLC6A2,SLC6A3,SLC6A11,SLC8A3,SLC9A2,SLC9A3,SLC18A3,SLCO2A1,SLIT1,SOX1,SOX3,SOX9,SOX10,SPTB,SRD5A2,SSTR1,SSTR2,ABCC8,TACR1,TAL1,TBX1,TBL1X,TBX2,TBX5,TCEA3,TBX3,HNF1B,TFAP2C,NR2F2,THBD,NKX2-1,ICAM5,TLL1,TLL2,TSPAN6,GPR137B,TNFRSF1B,TP73,TRH,TRPC5,TRPC6,TUBA4A,UCN,UCP1,VDR,VIPR2,WNT1,WNT2,WNT3,WNT6,WNT7A,WNT10B,WNT11,WT1,ZIC1,ZNF711,ZBTB16,SLC30A2,SLC30A3,SLC30A4,NPHS2,BTG2,PAX8,KCNAB1,ST8SIA4,NR4A3,ADAM12,ALX1,DPF3,ST8SIA2,DGCR6,EOMES,PIP5K1B,SOX14,IRS4,GAS7,BARX2,PIR,LGR5,AKR7A2,RGS20,PDE8B,UNC5C,CHRD,NOL4,TRADD,HRK,TNFRSF11A,GALR2,NRP1,ARHGEF7,CCNA1,CACNA1G,PHOX2B,CDK5R2,TRPA1,HAP1,CH25H,INA,SLC6A5,DGKI,REPS2,NOG,MSC,CRLF1,GPR50,KLF4,TCEAL1,SLIT2,LHX2,RAB33A,KL,LIPG,NTN1,ECEL1,HAND2,ONECUT2,NRG2,CXCL14,GABBR2,SPAG6,GDA,GNA14,FEZ1,PDE4DIP,FRMPD4,SPOCK2,ZEB2,LPPR4,SV2B,MAFB,AMMECR1,HS3ST4,HS3ST3B1,HS3ST2,USP3,HCN4,PTPRU,RASGRP1,OLIG2,IRX5,DLC1,NDRG1,ST3GAL6,SPON2,SPON1,HOXB13,SEMA4F,MAB21L2,RASL10A,DMRT2,LBX1,TBR1,SIX2,RAI2,ZMYND11,ALDH1L1,GPR83,GADD45G,RAB40B,SLC27A2,VAX1,RAB31,RIPK3,RAPGEF4,STMN2,ADAMTS5,PTPRT,IL1RAPL1,SOX21,TNRC4,FZD10,MGLL,NID2,ITGA11,IKZF3,HSPA4L,PPM1E,FRMPD1,VASH1,SLITRK3,ZNF365,DKK1,SORCS3,PDZD2,GLT25D2,RFTN1,PHLDB1,CYFIP1,TBC1D1,PLCB1,ASTN2,LPHN3,PPP1R13B,COMMD3,KCNH3,OTP,HEY1,MAPK8IP2,DDAH1,VSIG2,TMEFF2,IL17RA,FLRT2,C20orf103,TMEM59L,VAX2,ABTB2,DKFZP564O0823,CHRDL2,OLFML2B,C2orf32,GPR124,EGFL6,SLITRK5,NKX2-8,FBXO3,IL1RAPL2,FGF20,LHX6,CNNM1,KCNV1,FOXD3,FOXB1,DKK2,PCDH17,SRPX2,BHLHB5,PCDH11X,RPS6KA6,PCSK1N,SLCO4A1,C13orf15,STXBP6,PYCARD,ANKRD11,TMOD2,TRHDE,TBX21,SLC40A1,RAX,VSX1,WDR8,DUOX2,CUZD1,NEUROG3,KCNK4,IRX4,DHH,SPOCK3,TBX22,KCTD3,LEF1,NIN,RAB9B,IGF2AS,WIT1,SNX7,WNT16,HPCAL4,TPPP3,UPB1,TUBA8,DUOX1,RIPK4,CLIC6,GPR88,FLJ11235,APBB1IP,DLL4,HES2,SLC6A20,FEV,KIAA1546,DCHS2,GIPC2,SIDT1,PAQR5,FAM46C,RP11-35N6.1,PQLC2,CASZ1,FAM70A,SUSD4,FEZF2,CRTAC1,MCOLN3,RNF121,FBXL8,GIMAP5,STK32B,CAMK2N1,BATF3,TRIM36,ELMOD1,SLC30A10,PIGV,HHAT,CENTA2,MESP1,THSD1,GNG12,BARX1,PARD3,PRMT8,CTPS2,CYP26B1,KCNK13,KCNK12,BARHL1,DPYSL5,NDUFA4L2,CA10,ADAMTSL3,KIAA1199,CBX8,ZNF287,TTYH1,GJA9,TMEM27,ABHD6,DSCAML1,SLC12A5,GPR158,KIAA1324,ARHGAP20,LRCH2,ZFYVE28,FAM5B,CADM3,SLC46A2,NGB,CXCL16,SQRDL,DMRT3,C21orf63,PRDM12,ALOXE3,HPSE2,ALX4,PROK2,PKNOX2,NEUROG2,OXCT2,CDH23,CLSTN2,EPB41L4A,XYLT1,ABCG4,LHX5,ROBO3,SOX17,CHST8,HHIP,CSMD1,ISL2,IRX3,IRX1,BHLHB3,FAM65A,RNF128,C4orf31,YRDC,C1orf115,LONRF3,RIN3,FLJ13236,SCD5,GPR177,KIAA1772,SEMA6D,TUBA4B,ZNF703,FLJ21511,PIGZ,WNT10A,KCNIP4,ESX1,ZNF436,SETD7,COLEC12,LBH,TMEM163,KAZALD1,AMN,SLCO5A1,PCDH11Y,GPR101,RASSF5,SOX7,ESPN,FAM62C,ANKRD27,RAB6C,ZIC4,GPR103,TMEM164,ANKRD20A1,NBPF3,ZMYND15,LRRC8C,PRAC,PHYHIPL,MEGF11,NKX6-2,HOPX,ACSS1,COL25A1,NT5C1A,KIRREL3,NTNG2,SLITRK2,HES7,INSM2,OSAP,MAG1,ZNF503,ATOH8,CORO6,JUB,IGSF21,TOX2,TCEAL3,COL27A1,KIAA1666,NKD1,TSLP,WNT3A,NAV2,C3orf15,LHX4,DUOXA1,ESAM,SCARF2,SLFN11,SYT12,TMEM88,RCSD1,OTOP2,DNER,CGB7,CGB8,SYTL4,TIP39,TMEM54,C21orf81,TRIM9,CSMD3,SLITRK1,SORCS1,OSR2,LRRC3B,RBP7,LYPD1,GRIN3A,OLIG1,FAM129A,DACH2,ASCL4,KLHDC1,SLC24A4,NOXO1,USH1G,TMEM132E,B4GALNT2,C1orf194,PODN,C1orf122,BHLHB4,FBLN7,SGPP2,OSR1,SPATA18,ARL9,OTOP1,EGFLAM,KLF14,LETM2,ANKRD19,SLITRK4,PTCHD1,CHODL,TCEAL2,GATA5,SLC32A1,CBLN4,MT1P3,LAYN,PRICKLE1,GSC,THSD3,LRFN5,NLF1,FAM81A,CMTM2,MGAT5B,CCBE1,SIX5,SYT6,SLC35F3,C1orf76,MFSD4,C1orf213,C1orf92,LOC150221,FLJ32063,CCDC140,FAM84A,GDF7,FAM19A4,C5orf38,LOC153684,AMOT,RDH10,RASEF,TCEAL6,NKX2-3,CLEC14A,TMEM30B,EML5,NAGS,FBXO39,FAM43B,APCDD1L,DCLK2,CDC20B,ZCCHC12,ADAMTS15,ADAMTS18,GSX2,RDHE2,FAM24B,ADCY4,C15orf51,GGN,NBPF11,FOXD4L1,VWA3B,SLC10A4,C4orf34,HTRA4,C8orf47,LGI3,NRK,FAM123A,GSX1,TMEM26,UNC5B,DOK6,OAF,ANKRD18A,EBF3,LCORL,C4orf22,COL24A1,PCSK9,NPNT,PTF1A,LYSMD2,MAP7D2,FRMD3,CYP4X1,HS6ST3,NPAS4,MKX,FLJ33790,LOC283392,DPY19L2,LOC283514,METRNL,ZADH2,RSPO1,EPHA10,FAM80A,FOXD4L2,MGC39900,EIF4E3,ZAR1,MT1DP,GPR120,VSX2,CLEC4G,FAM5C,TFAP2E,KY,RSPO2,RBM32A,KIAA2022,ZCCHC16,CYP26C1,HMX3,SYT10,BARHL2,C2orf55,FIGLA,LRRTM1,OTOP3,MGC52498,DPY19L2P2,FOXD4L4,RTN4RL2,BMP8A,GALNTL4,SFT2D2,FAM89A,FOXD4L3,C1orf32,TMEM46,FLJ45455,RPRML,C17orf82,RGS9BP,HS6ST1P,NOTCH2NL,FLJ39739,ANKRD20B,FLJ46347,C5orf39,UNQ9433,IER5L,ARL5C,GDF6,SHC4,FLJ45983,LOC399947,LOC400120,LOC400464,FLJ44815,FLJ35409,FLJ45832,LOC401611,MGC39545,DUOXA2,LHX8,DBX2,MGC26718,TRIM67,LOC440804,LOC441413,ANKRD20A3,LOC441426,ANKRD20A2,ANKRD18B,,

Eed.targets ADARB2,ADCY8,ADCYAP1,ADM,ADRA1A,ADRA2A,ADRB1,ADRB3,ALOX5,ALOX15,ALX3,AQP5,PHOX2A,ASCL1,ASCL2,ASTN1,ZFHX3,ATF3,ATOH1,NKX3-2,BCL2,BNC1,BMI1,BMP3,FOXL2,CA3,CACNA1B,CACNA1D,CACNA1E,CALCA,CBLN1,CBR3,CD8A,CD34,CD44,CD70,CDH7,CDKN2C,CDX2,CEBPD,CFTR,CIDEA,CLCN5,CNTFR,COL2A1,COL4A5,COL4A6,COL9A2,COMP,CRHBP,CRHR1,CRYBA2,CTNND2,CYP1B1,CYP24A1,CYP26A1,CYP27B1,DACH1,DGKG,DCC,COCH,DIO3,DLX1,DLX2,DLX3,DLX4,DMRT1,DRD5,DSC3,DUSP4,EFNA1,EFNA3,EGR3,EGR4,EMX2,EN1,EN2,EPAS1,EPHA5,EPHB1,EPHB3,EPS8,ERBB4,EYA2,F2R,FBN2,FBP1,FGF3,FGF5,FGF9,GPC5,FOXG1,FOXF1,FOXL1,FOXJ1,FOXE1,FOXD2,FLI1,AFF2,FUT4,FZD2,GABRA2,GABRA4,GAD2,B4GALNT1,GATA2,GATA3,GATA4,GATA6,GBX2,GDNF,GFI1,GFRA1,GHR,GHSR,GJB2,GPM6B,PRLHR,GPR12,MLNR,GRIA2,GRID1,GRIK1,GRIK3,GRM7,GSC2,GUCY1A3,GUCY2D,HBA1,HBA2,NRG1,HHEX,MNX1,HLF,HLX,HMX2,FOXA2,ONECUT1,TLX1,TLX2,HOXB1,HOXB2,HOXB3,HOXB6,HOXB7,HOXB8,HOXC4,HOXC5,HOXC6,HOXC8,HOXC9,HOXC11,HOXC12,HOXC13,HOXD1,HOXD3,HOXD4,HOXD8,HOXD9,HOXD11,HOXD12,HOXD13,HSF4,HSPA6,HTR1A,HTR2C,HTR7,IGF2,IL7,INSRR,PDX1,ISL1,ITGA4,ITPKA,JUN,KAL1,KCNA1,KCNA3,KCNC2,KCNC4,KCND3,KCNH1,KCNJ2,KCNJ6,KCNK2,KCNMA1,KCNQ3,LGALS3,LMX1B,LOX,LPL,LRP2,LTBP2,LTK,MAB21L1,MAF,MAL,MAPT,MID1,MLLT3,CITED1,MSX1,MT1A,MT1B,MT1H,MT1JP,MT1M,MT1X,MYF6,MYO5B,MYOD1,NCAM1,NEFM,NELL1,NEFL,NEUROD1,NEUROD2,NEUROG1,NFIX,NKX2-2,NKX3-1,NKX6-1,NPAS1,NPR3,NPTX1,NPY1R,NRCAM,NTRK1,NTRK2,OCA2,OPRD1,SIX6,OTX1,OTX2,PAPPA,PAX1,PAX2,PAX3,PAX6,PAX7,PAX9,PCDH8,PDGFRA,SLC26A4,PENK,PGM5,PGR,PITX1,PITX2,PITX3,PKP1,PLEC1,PLXNA2,PMP22,POLE,POU3F1,POU3F4,POU4F1,POU4F2,POU4F3,PRKCE,PRKG1,MAPK4,HTRA1,PYY,PTGDR,PTGER2,PTGER3,PTGER4,PTGFR,PTHLH,PXMP2,RASGRF1,RBP4,RGS10,RYR3,SCN4B,SCNN1G,SCTR,SDC2,SFRP1,SFRP5,SHH,SHOX,SHOX2,ST8SIA1,SIM2,SIX1,SIX3,SLC1A2,SLC1A4,SLC6A1,SLC6A3,SLC8A3,SLC9A2,SLC9A3,SLC18A3,SLCO2A1,SLIT1,SOX1,SOX3,SRD5A2,SSTR1,SSTR2,ABCC8,TAL1,TBX1,TBX2,TBX5,TCEA3,TBX3,HNF1B,NR2F2,THBD,NKX2-1,ICAM5,TLL1,TSPAN6,TP73,TRH,TRPC5,TUBA4A,UCN,UCP1,VDR,VIPR2,WNT1,WNT2,WNT6,WNT7A,WNT10B,WNT11,WT1,ZIC1,ZNF711,ZBTB16,SLC30A2,SLC30A3,SLC30A4,BTG2,PAX8,KCNAB1,NR4A3,ALX1,DPF3,ST8SIA2,EOMES,PIP5K1B,SOX14,BARX2,PIR,LGR5,RGS20,UNC5C,CHRD,NOL4,TRADD,HRK,TNFRSF11A,GALR2,CACNA1G,PHOX2B,CDK5R2,TRPA1,HAP1,CH25H,INA,SLC6A5,DGKI,REPS2,NOG,MSC,CRLF1,GPR50,KLF4,SLIT2,LHX2,KL,NTN1,ECEL1,HAND2,ONECUT2,NRG2,CXCL14,SPAG6,GDA,GNA14,FEZ1,PDE4DIP,FRMPD4,ZEB2,SV2B,MAFB,HS3ST3B1,PTPRU,RASGRP1,OLIG2,IRX5,SPON1,HOXB13,MAB21L2,RASL10A,DMRT2,LBX1,TBR1,SIX2,RAI2,GADD45G,SLC27A2,VAX1,RIPK3,STMN2,ADAMTS5,PTPRT,SOX21,FZD10,IKZF3,PPM1E,VASH1,SLITRK3,ZNF365,DKK1,SORCS3,PDZD2,GLT25D2,PLCB1,ASTN2,LPHN3,KCNH3,OTP,HEY1,DDAH1,VSIG2,TMEFF2,FLRT2,C20orf103,TMEM59L,VAX2,ABTB2,DKFZP564O0823,CHRDL2,OLFML2B,C2orf32,EGFL6,NKX2-8,FBXO3,IL1RAPL2,FGF20,LHX6,CNNM1,KCNV1,FOXD3,FOXB1,DKK2,PCDH17,BHLHB5,RPS6KA6,PCSK1N,SLCO4A1,C13orf15,STXBP6,TMOD2,TRHDE,TBX21,RAX,VSX1,WDR8,DUOX2,NEUROG3,KCNK4,IRX4,DHH,SPOCK3,TBX22,IGF2AS,WIT1,WNT16,HPCAL4,TPPP3,DUOX1,GPR88,FLJ11235,APBB1IP,DLL4,HES2,FEV,KIAA1546,DCHS2,SIDT1,RP11-35N6.1,CASZ1,SUSD4,FEZF2,CRTAC1,MCOLN3,FBXL8,GIMAP5,STK32B,CAMK2N1,BATF3,TRIM36,ELMOD1,HHAT,CENTA2,MESP1,BARX1,PARD3,CYP26B1,KCNK13,KCNK12,BARHL1,NDUFA4L2,CA10,KIAA1199,CBX8,TTYH1,GJA9,TMEM27,DSCAML1,KIAA1324,ARHGAP20,LRCH2,ZFYVE28,FAM5B,NGB,CXCL16,DMRT3,C21orf63,PRDM12,HPSE2,ALX4,PROK2,PKNOX2,NEUROG2,OXCT2,CDH23,CLSTN2,EPB41L4A,LHX5,ROBO3,SOX17,CHST8,HHIP,CSMD1,ISL2,IRX3,BHLHB3,RNF128,YRDC,LONRF3,RIN3,FLJ13236,SCD5,GPR177,KIAA1772,SEMA6D,TUBA4B,PIGZ,WNT10A,ESX1,ZNF436,SETD7,COLEC12,KAZALD1,SLCO5A1,GPR101,RASSF5,SOX7,ESPN,FAM62C,ANKRD27,RAB6C,ZIC4,GPR103,ANKRD20A1,ZMYND15,PRAC,MEGF11,NKX6-2,HOPX,COL25A1,NT5C1A,KIRREL3,NTNG2,HES7,INSM2,OSAP,MAG1,ZNF503,ATOH8,CORO6,IGSF21,COL27A1,KIAA1666,TSLP,WNT3A,NAV2,C3orf15,LHX4,DUOXA1,ESAM,SLFN11,SYT12,TMEM88,OTOP2,CGB7,CGB8,TIP39,TRIM9,CSMD3,SLITRK1,SORCS1,LYPD1,GRIN3A,OLIG1,DACH2,SLC24A4,USH1G,TMEM132E,B4GALNT2,C1orf194,PODN,C1orf122,BHLHB4,FBLN7,SGPP2,OSR1,ARL9,OTOP1,ANKRD19,SLITRK4,CHODL,SLC32A1,CBLN4,MT1P3,LAYN,PRICKLE1,GSC,LRFN5,NLF1,FAM81A,CMTM2,CCBE1,SIX5,SLC35F3,C1orf76,C1orf213,C1orf92,LOC150221,FLJ32063,CCDC140,FAM84A,GDF7,FAM19A4,C5orf38,LOC153684,AMOT,NKX2-3,CLEC14A,TMEM30B,EML5,NAGS,FBXO39,FAM43B,DCLK2,ADAMTS15,ADAMTS18,GSX2,ADCY4,NBPF11,FOXD4L1,VWA3B,SLC10A4,C8orf47,GSX1,TMEM26,DOK6,OAF,ANKRD18A,LCORL,C4orf22,COL24A1,NPNT,PTF1A,LYSMD2,MAP7D2,FRMD3,HS6ST3,NPAS4,MKX,FLJ33790,LOC283392,DPY19L2,METRNL,ZADH2,RSPO1,FAM80A,FOXD4L2,MT1DP,GPR120,VSX2,FAM5C,TFAP2E,KY,RSPO2,RBM32A,ZCCHC16,CYP26C1,HMX3,SYT10,BARHL2,C2orf55,FIGLA,LRRTM1,OTOP3,DPY19L2P2,FOXD4L4,RTN4RL2,BMP8A,GALNTL4,FAM89A,FOXD4L3,C1orf32,TMEM46,FLJ45455,RPRML,C17orf82,RGS9BP,HS6ST1P,ANKRD20B,FLJ46347,C5orf39,UNQ9433,IER5L,GDF6,SHC4,FLJ45983,LOC399947,LOC400120,FLJ44815,FLJ35409,MGC39545,DUOXA2,LHX8,MGC26718,TRIM67,LOC440804,LOC441413,ANKRD20A3,LOC441426,ANKRD20A2,ANKRD18B,ACYP2,AHR,AIM1,ARNTL,ASAH1,ATP1B3,BMP6,BMPR1B,CALCR,CAMK2B,RUNX2,CHN2,TBCB,COL12A1,COL19A1,CSNK1E,NKX2-5,CTGF,CTSD,DFNA5,DLX5,DRD4,DSP,EBF1,EEF1D,EEF2,EGFR,ELAVL2,ELAVL1,EYA4,EVX1,FARSA,FGF10,FOXF2,FOXC1,FOS,FUCA2,GABRB2,GAD1,GPR6,GRK5,GRM3,GSTA4,H1F0,H2AFX,HIC1,HIP2,HLA-A,HLA-B,HLA-C,HLA-G,HOXA1,HOXA2,HOXA3,HOXA4,HOXA6,HOXA7,HOXA9,HOXA10,HOXA13,HSPA1A,HSPA1B,HSPA1L,HTR1B,HTR1E,IGFBP3,IGFBP7,INHBB,INPP4A,KCNQ1,LMO1,MAP3K3,MAP3K5,MEOX2,MICB,MSX2,MYB,HNRPM,NPPC,NPTX2,P2RX5,PDE8A,POLR2I,POU3F2,PPP1R10,PRKCB1,RELN,CXCL12,SFRP4,SIM1,SLC22A3,SOD3,SOX5,T,TAC1,TAP1,TCF15,TCF21,TFAP2B,TGFA,TGFB2,NR2E1,TTPA,TWIST1,VGF,ZIC2,PLA2G7,SF3A2,ARID1A,HIST1H4I,CDC7,FZD1,FZD8,HIST1H2AI,HIST1H2AK,HIST1H2AL,HIST1H2AM,HIST2H2AA3,HIST1H2BL,HIST1H2BO,HIST1H3A,HIST1H3D,HIST1H3C,HIST1H3E,HIST1H3I,HIST1H3G,HIST1H3J,HIST1H3H,HIST1H3B,HIST1H4A,HIST1H4D,HIST1H4F,HIST1H4K,HIST1H4J,HIST1H4C,HIST1H4H,HIST1H4B,HIST1H4E,HIST1H4L,HIST2H4A,CUL4A,STC2,TNFRSF25,C19orf2,DLK1,HIST1H3F,HIST1H2AG,WASL,NEURL,DCLK1,GCM2,STK17A,B4GALT6,HAND1,KLK4,RGS6,MORF4L2,DLG7,MTSS1,SCRN1,GINS1,MAGI2,SNAP91,BCL2L10,YAF2,PLXNC1,MPHOSPH6,HCG9,ABCA8,BAIAP2,HYOU1,TBL3,CTCF,PDE10A,PAPOLA,RAB32,HHLA3,BVES,IRAK3,NXPH4,U2AF2,DENND3,RP4-691N24.1,PLXND1,ZCCHC14,PHF3,SRRM2,MOXD1,AUTS2,RSL1D1,AATF,INTU,PRELID1,MRPS18B,UCRC,ST8SIA5,TLX3,MED31,NDUFA13,NRN1,ZBTB7A,ETV7,C9orf156,OTUD6B,TM6SF1,CLIC5,IL20RA,RAB24,EPB41L4B,BTG4,CNNM2,ZCCHC2,ELOVL2,PLEKHJ1,DARS2,SHQ1,GALNT10,PNRC2,EDEM2,PCID2,SELS,ZMAT5,KCNQ5,NRIP3,TBX20,GPR126,LRRC47,LRFN2,SORCS2,PCDH10,NTN4,PRDM13,BACH2,ZNF335,DMRTC1,PRDM14,PARP12,UPF3A,TMEM106C,APOO,OGFRL1,STEAP4,CLIP4,ABHD9,C6orf97,FUZ,ULBP2,ULBP1,HCCA2,ANKRD13C,CDT1,PPP1R14C,TRIM7,TFAP2D,ATAD3B,TSSK6,PSD2,SLC25A33,MCHR2,FNDC1,RSPO3,TIGD5,HIST1H2AH,HIST1H2BK,SCIN,KCNK17,TCEAL8,UAP1L1,CENPL,FOXQ1,LOC116236,HIST4H4,ANKRD9,LOC130074,ACVR1C,SHROOM1,C7orf57,ARID3C,DOCK11,FBXL14,DHRS13,C9orf19,NKAIN2,TMEM65,OLIG3,ASXL1,C10orf72,CREG2,CXorf50,RNF152,JMJD1C,RBM24,MMD2,RFXDC1,SCUBE3,FERD3L,VGLL2,CXorf58,MDGA1,NKPD1,FLJ25037,SLC35D3,ZNF517,LHFPL3,PTAR1,SLC6A10P,hCG_1985469,LOC389039,OR8A1,FLJ16165,MED11,CUEDC1,FLJ90757,LOC441189,, ,

H3K27.bound ADARB2,ADCY8,ADCYAP1,ADRA1A,ADRA2A,ADRB1,ADRB3,ALOX15,ALX3,AQP5,PHOX2A,ASCL1,ASCL2,ASTN1,ZFHX3,ATF3,ATOH1,NKX3-2,BCL2,BNC1,FOXL2,CACNA1B,CACNA1D,CACNA1E,CALCA,CBLN1,CBR3,CD8A,CD34,CD70,CDH7,CDKN2C,CDX2,CIDEA,CLCN5,CNTFR,COL2A1,COL4A5,COL4A6,COL9A2,COMP,CRHR1,CRYBA2,CTNND2,CYP24A1,CYP26A1,CYP27B1,DACH1,DGKG,DCC,DIO3,DLX1,DLX2,DLX3,DLX4,DMRT1,DRD5,DSC3,DUSP4,EFNA1,EFNA3,EGR3,EGR4,EN1,EN2,EPAS1,EPHA5,EPHB1,EPHB3,ERBB4,F2R,FBN2,FBP1,FGF3,FGF5,FGF9,GPC5,FOXG1,FOXF1,FOXL1,FOXJ1,FOXE1,FOXD2,FLI1,FUT4,FZD2,GABRA2,GABRA4,GAD2,B4GALNT1,GATA2,GATA3,GATA4,GATA6,GBX2,GDNF,GHR,GHSR,GJB2,GPM6B,PRLHR,GPR12,GRIA2,GRID1,GRIK1,GRIK3,GRM7,GSC2,GUCY1A3,GUCY2D,HBA1,HBA2,NRG1,HHEX,MNX1,HLX,HMX2,FOXA2,ONECUT1,TLX1,TLX2,HOXB1,HOXB2,HOXB3,HOXB6,HOXB7,HOXB8,HOXC4,HOXC5,HOXC6,HOXC8,HOXC11,HOXC12,HOXD1,HOXD3,HOXD4,HOXD8,HOXD9,HOXD12,HOXD13,HSF4,HSPA6,HTR1A,HTR2C,HTR7,IL7,INSRR,PDX1,ISL1,ITGA4,ITPKA,JUN,KCNA1,KCNA3,KCNC2,KCNC4,KCND3,KCNH1,KCNK2,KCNMA1,KCNQ3,LGALS3,LMX1B,LPL,LRP2,LTBP2,LTK,MAB21L1,MAL,MAPT,MLLT3,CITED1,MSX1,MT1A,MT1B,MT1H,MT1M,MYF6,MYO5B,MYOD1,NCAM1,NEFM,NELL1,NEFL,NEUROD1,NEUROD2,NEUROG1,NFIX,NKX2-2,NKX3-1,NKX6-1,NPAS1,NPR3,NPTX1,NPY1R,NTRK1,NTRK2,OCA2,OPRD1,SIX6,OTX1,OTX2,PAPPA,PAX1,PAX2,PAX3,PAX6,PAX7,PAX9,PCDH8,PDGFRA,SLC26A4,PENK,PGM5,PGR,PITX1,PITX2,PITX3,PKP1,PLEC1,PLXNA2,PMP22,POLE,POU3F1,POU3F4,POU4F1,POU4F2,POU4F3,PRKCE,PRKG1,MAPK4,PYY,PTGDR,PTGER2,PTGER3,PTGER4,PTGFR,PTHLH,PXMP2,RASGRF1,RBP4,RGS10,RYR3,SCN4B,SCNN1G,SCTR,SFRP1,SFRP5,SHH,SHOX,SHOX2,SIM2,SIX1,SIX3,SLC1A2,SLC1A4,SLC6A1,SLC6A3,SLC9A2,SLC9A3,SLCO2A1,SLIT1,SRD5A2,SSTR1,SSTR2,ABCC8,TAL1,TBX1,TBX2,TBX5,TCEA3,TBX3,HNF1B,NR2F2,THBD,NKX2-1,ICAM5,TLL1,TP73,TRH,TRPC5,UCN,UCP1,VDR,WNT1,WNT2,WNT6,WNT7A,WNT10B,WNT11,WT1,ZIC1,ZBTB16,SLC30A2,SLC30A3,SLC30A4,BTG2,PAX8,KCNAB1,NR4A3,DPF3,ST8SIA2,EOMES,PIP5K1B,SOX14,BARX2,PIR,LGR5,RGS20,UNC5C,CHRD,NOL4,TRADD,HRK,GALR2,CACNA1G,PHOX2B,CDK5R2,CH25H,INA,SLC6A5,DGKI,REPS2,MSC,CRLF1,KLF4,SLIT2,LHX2,KL,NTN1,ECEL1,HAND2,ONECUT2,NRG2,CXCL14,SPAG6,GNA14,FEZ1,PDE4DIP,FRMPD4,ZEB2,SV2B,MAFB,HS3ST3B1,PTPRU,OLIG2,IRX5,SPON1,HOXB13,MAB21L2,RASL10A,DMRT2,LBX1,TBR1,SIX2,SLC27A2,VAX1,RIPK3,STMN2,PTPRT,FZD10,IKZF3,PPM1E,VASH1,SLITRK3,DKK1,SORCS3,PDZD2,GLT25D2,ASTN2,LPHN3,KCNH3,OTP,HEY1,DDAH1,TMEFF2,FLRT2,C20orf103,TMEM59L,VAX2,ABTB2,DKFZP564O0823,CHRDL2,OLFML2B,C2orf32,EGFL6,NKX2-8,FBXO3,IL1RAPL2,FGF20,LHX6,CNNM1,KCNV1,FOXD3,FOXB1,DKK2,PCDH17,BHLHB5,RPS6KA6,C13orf15,STXBP6,TMOD2,TBX21,RAX,VSX1,WDR8,DUOX2,NEUROG3,KCNK4,IRX4,DHH,SPOCK3,IGF2AS,WIT1,WNT16,HPCAL4,TPPP3,DUOX1,GPR88,FLJ11235,DLL4,HES2,FEV,KIAA1546,DCHS2,SIDT1,RP11-35N6.1,CASZ1,SUSD4,FEZF2,CRTAC1,MCOLN3,FBXL8,GIMAP5,STK32B,CAMK2N1,BATF3,TRIM36,ELMOD1,HHAT,CENTA2,MESP1,BARX1,CYP26B1,KCNK13,KCNK12,BARHL1,NDUFA4L2,CA10,KIAA1199,CBX8,TTYH1,GJA9,TMEM27,DSCAML1,KIAA1324,ARHGAP20,LRCH2,ZFYVE28,FAM5B,CXCL16,DMRT3,C21orf63,PRDM12,HPSE2,ALX4,PROK2,PKNOX2,NEUROG2,OXCT2,CDH23,CLSTN2,EPB41L4A,LHX5,ROBO3,SOX17,CHST8,HHIP,CSMD1,ISL2,IRX3,BHLHB3,RNF128,LONRF3,FLJ13236,SCD5,SEMA6D,PIGZ,WNT10A,ESX1,ZNF436,COLEC12,KAZALD1,SLCO5A1,GPR101,RASSF5,SOX7,ESPN,ANKRD27,RAB6C,ZIC4,ANKRD20A1,ZMYND15,PRAC,NKX6-2,COL25A1,NT5C1A,KIRREL3,NTNG2,HES7,INSM2,OSAP,MAG1,ZNF503,ATOH8,CORO6,IGSF21,COL27A1,KIAA1666,TSLP,WNT3A,NAV2,C3orf15,LHX4,DUOXA1,ESAM,SLFN11,SYT12,TMEM88,OTOP2,CGB7,CGB8,TIP39,TRIM9,CSMD3,SLITRK1,SORCS1,GRIN3A,DACH2,SLC24A4,USH1G,TMEM132E,B4GALNT2,C1orf194,PODN,BHLHB4,FBLN7,SGPP2,OSR1,ARL9,OTOP1,ANKRD19,CHODL,SLC32A1,CBLN4,LAYN,GSC,LRFN5,NLF1,FAM81A,CMTM2,SLC35F3,C1orf76,C1orf213,C1orf92,LOC150221,FLJ32063,CCDC140,FAM84A,GDF7,FAM19A4,LOC153684,NKX2-3,CLEC14A,TMEM30B,NAGS,FAM43B,DCLK2,ADAMTS15,ADAMTS18,GSX2,ADCY4,NBPF11,FOXD4L1,SLC10A4,C8orf47,GSX1,DOK6,OAF,ANKRD18A,COL24A1,NPNT,PTF1A,LYSMD2,FRMD3,HS6ST3,NPAS4,MKX,FLJ33790,DPY19L2,METRNL,ZADH2,RSPO1,FAM80A,FOXD4L2,MT1DP,GPR120,VSX2,FAM5C,TFAP2E,KY,RSPO2,RBM32A,ZCCHC16,CYP26C1,HMX3,BARHL2,FIGLA,LRRTM1,OTOP3,DPY19L2P2,FOXD4L4,RTN4RL2,BMP8A,GALNTL4,FAM89A,FOXD4L3,C1orf32,FLJ45455,RPRML,C17orf82,RGS9BP,HS6ST1P,ANKRD20B,FLJ46347,C5orf39,UNQ9433,GDF6,SHC4,FLJ45983,LOC400120,FLJ44815,FLJ35409,MGC39545,DUOXA2,LHX8,MGC26718,TRIM67,LOC440804,LOC441413,ANKRD20A3,LOC441426,ANKRD20A2,ANKRD18B,AIM1,ARNTL,BMP6,CALCR,CAMK2B,RUNX2,CHN2,COL12A1,NKX2-5,DLX5,DRD4,EBF1,ELAVL2,EYA4,EVX1,FOXF2,FOXC1,GABRB2,GPR6,GRK5,HIC1,HLA-A,HLA-B,HLA-C,HOXA1,HOXA2,HOXA3,HOXA4,HOXA6,HOXA7,HOXA9,HOXA10,HOXA13,HSPA1A,HSPA1B,HSPA1L,IGFBP3,KCNQ1,LMO1,MEOX2,MICB,MSX2,MYB,P2RX5,PDE8A,POU3F2,PRKCB1,SFRP4,SIM1,SLC22A3,T,TAC1,TAP1,TCF21,TFAP2B,TGFA,NR2E1,TTPA,TWIST1,FZD1,STC2,DLK1,NEURL,DCLK1,GCM2,HAND1,KLK4,YAF2,PLXNC1,HCG9,PDE10A,NXPH4,AATF,ST8SIA5,TLX3,MED31,NRN1,ZBTB7A,ETV7,CLIC5,CNNM2,ELOVL2,KCNQ5,NRIP3,TBX20,LRFN2,PRDM13,TMEM106C,CLIP4,ULBP2,ULBP1,PPP1R14C,TRIM7,TFAP2D,PSD2,FNDC1,RSPO3,SCIN,KCNK17,TCEAL8,FOXQ1,ARID3C,FBXL14,C9orf19,NKAIN2,OLIG3,MMD2,RFXDC1,SCUBE3,FERD3L,VGLL2,NKPD1,FLJ25037,SLC35D3,LHFPL3,hCG_1985469,FLJ16165,LOC441189,ACADL,ADRBK2,ACAN,BAI2,BMX,CACNA1A,CACNB4,CD38,CDH6,CRIP1,CRMP1,CSF1,CYP2J2,DPP6,DUSP6,EGR2,ELAVL3,EPHA4,FBN1,FOXE3,GPC3,GRIN2D,HTR6,ICAM4,IHH,IMPDH1,KCNA5,KCNJ3,TACSTD2,MAP6,MDS1,NBL1,NEFH,NFIA,NFIC,NGFB,NGFR,NPAS2,NPY5R,OVOL1,PAX5,POMC,PRKCH,RBBP7,RXRG,SECTM1,SLC6A2,SLC6A11,SOX9,SPTB,TACR1,TNFRSF1B,ST8SIA4,PDE8B,ARHGEF7,CCNA1,TCEAL1,RAB33A,GABBR2,HS3ST4,HS3ST2,HCN4,NDRG1,SPON2,SEMA4F,RAB40B,RAPGEF4,FRMPD1,PHLDB1,PPP1R13B,COMMD3,MAPK8IP2,GPR124,SLC40A1,LEF1,NIN,RAB9B,SLC6A20,SLC30A10,DPYSL5,ALOXE3,XYLT1,ABCG4,IRX1,C1orf115,FLJ21511,KCNIP4,TMEM163,AMN,PHYHIPL,RCSD1,DNER,TMEM54,OSR2,RBP7,FAM129A,ASCL4,NOXO1,EGFLAM,SYT6,MFSD4,CDC20B,LGI3,FAM123A,UNC5B,EBF3,EPHA10,EIF4E3,CLEC4G,MGC52498,FLJ39739,ARL5C,ACCN2,AMELX,AMPH,APBA1,ATP1A3,AVPR1B,BLVRB,CA7,CACNB3,ENTPD2,ENTPD3,CD47,LRBA,CDH4,CDX1,CHAD,COL8A1,CPM,CR2,CRH,CYP2A13,DCX,DYNC1I1,DTNB,DUSP8,EPHA3,ESR1,FGF8,FGF11,FUCA1,GAST,GABRG3,GNAO1,GPR26,GRB10,GRIN1,GRM6,GSN,HLA-F,IGFBP1,IL10RA,IL11,IRF4,KCNF1,KCNJ4,KCNJ9,KCNJ10,KCNK3,KCNS2,KCNS3,LAMB1,LY6H,MEIS1,MMP2,MT3,NOVA2,NPR1,NRGN,PCSK2,PDE1B,PLP1,PRKD1,PTPRN2,RARA,RARRES2,RFX4,RNF2,RPS6KA2,SFTPC,SH3GL2,SLIT3,SNAI2,SNTB1,STX1A,CNTN2,TBXAS1,THBS2,TLE2,SEMA3B,LHX3,MADCAM1,PRSS12,CBX4,SKAP1,ABCC3,TNFSF9,ADAM15,RGS9,SYT7,LRAT,NRXN1,SLC25A27,SNCAIP,RAB11FIP3,FRAT1,TRIM28,RAMP1,CACNG3,VAV3,FBLN5,FUT9,HPSE,C1QL1,PADI2,GPD1L,WSCD1,SATB2,SYNE1,OSBP2,LMOD1,CHD5,NOC2L,FBXO25,HEYL,SLCO3A1,DDX25,SOX8,KCNIP2,CRYL1,MLXIPL,PLLP,CYP39A1,ACTL6B,PRKAG2,IL17D,ADAM22,FXYD7,FLJ20184,CHDH,SMPD3,IL17RB,HR,PCDHGC4,ERO1LB,C14orf132,SLC17A7,SLC17A6,MAN1C1,RNPEPL1,KIAA1191,GALNTL1,KLHL14,KLHL1,SPTBN4,GRHL3,CACNG8,BCAN,SMOC2,REEP1,DLK2,MLPH,GCC1,EFCAB1,PARP8,TTLL7,GKAP1,ITIH5,PRRT1,WNT5B,ELOVL3,RTBDN,C8orf13,ARID5B,POLR3GL,PCGF5,KIAA1853,TMEM185A,KISS1R,GPR174,FLJ14816,C1orf94,LOC89944,KCNH7,KLHL13,EXOC3L2,CHRDL1,HTRA3,CAMK2N2,ADC,CSMD2,C1QTNF5,CENTG1,PARD3B,GPR62,SEZ6,COX6B2,DUSP15,CNTNAP5,ANKRD43,C6orf159,GAB3,RIMS4,NOTUM,SAMD11,C1orf51,ANKRD35,CCDC50,LOC154761,PRUNE2,TMEM20,ZFPM1,SLC16A11,TTLL9,MGC42105,SSBP4,ADAMTS17,CADM4,TCTE1,C11orf45,TIGD3,FNDC5,RASGEF1C,TMEM151,NEGR1,CCDC96,PRR10,ARL10,GPR150,PRR18,FAM139A,FAM78A,KLHL17,RGAG4,LOC342897,FAM70B,LGICZ1,CERKL,MAST4,SLC26A5,HES5,C3orf54,FLJ16641,HES3,MXRA7

PRC2.targets ABCC8,ABTB2,ADAMTS15,ADAMTS18,ADARB2,ADCY4,ADCY8,ADCYAP1,ADRA1A,ADRA2A,ADRB1,ADRB3,ALOX15,ALX3,ALX4,ANKRD19,ANKRD20A1,ANKRD20B,ANKRD27,AQP5,ARHGAP20,ARL9,ASCL1,ASCL2,ASTN1,ASTN2,ZFHX3,ATF3,ATOH1,ATOH8,NKX3-2,BARHL1,BARHL2,BARX1,BARX2,BCL2,BHLHB3,BHLHB4,BHLHB5,BMP8A,BNC1,BTG2,MKX,TMEM59L,FAM89A,C1orf32,C1orf76,C1orf92,C20orf103,C21orf63,C2orf32,C3orf15,CA10,CACNA1B,CACNA1D,CACNA1E,CACNA1G,CALCA,CAMK2N1,CASZ1,CBLN1,CBLN4,CBR3,CBX8,CD34,CD8A,CDH23,CDH7,CDK5R2,CDKN2C,CDX2,CENTA2,CGB7,CGB8,TPPP3,CH25H,CHODL,CHRD,CHRDL2,CHST8,VSX2,CIDEA,CITED1,CMTM2,CLCN5,CLEC14A,CLSTN2,CNNM1,CNTFR,COL24A1,COL25A1,COL27A1,COL2A1,COL4A5,COL4A6,COL9A2,COLEC12,COMP,CORO6,CRHR1,CRLF1,CRTAC1,CRYBA2,CSMD1,CSMD3,CTNND2,GJA9,CXCL14,CXCL16,CYP24A1,CYP26A1,CYP26B1,CYP26C1,CYP27B1,DACH1,DACH2,DCLK2,DCC,DCHS2,DDAH1,DGKG,DGKI,DHH,DIO3,DKFZP564O0823,DKK1,DKK2,DLL4,DLX1,DLX2,DLX3,DLX4,DMRT1,DMRT2,DMRT3,DOK6,DPF3,DPY19L2,DRD5,DSC3,DSCAML1,DUOX1,DUOX2,DUSP4,ECEL1,EFNA1,EFNA3,EGFL6,EGR3,EGR4,ELMOD1,EN1,EN2,EOMES,EPAS1,EPB41L4A,EPHA5,EPHB1,EPHB3,ERBB4,ESAM,ESPN,ESX1,F2R,FAM19A4,FAM43B,FAM5B,FAM5C,FAM80A,FAM84A,FBN2,FBP1,FBXL8,FBXO3,FEV,FEZ1,FGF20,FGF3,FGF5,FGF9,FIGLA,FLI1,FLJ11235,FLJ13236,KIAA1546,FLJ32063,CCDC140,FLJ33790,SLFN11,FLJ35409,ANKRD18A,DPY19L2P2,FBLN7,C8orf47,FLJ44815,FLJ45455,FLJ45983,FLJ46347,FLRT2,FOXA2,FOXB1,FOXD2,FOXD3,FOXD4L4,FOXD4L1,FOXD4L2,FOXD4L3,FOXE1,FOXF1,FOXG1,FOXJ1,FOXL1,FOXL2,FRMD3,FUT4,FZD10,FZD2,GABRA2,GABRA4,GAD2,B4GALNT1,B4GALNT2,GALNTL4,GALR2,GATA2,GATA3,GATA4,GATA6,GBX2,GDF6,GDF7,GDNF,GHR,GHSR,GIMAP5,GJB2,GLT25D2,GNA14,GPC5,GPM6B,PRLHR,GPR101,GPR12,GPR120,GPR88,GRIA2,GRID1,GRIK1,GRIK3,GRIN3A,GRM7,GSC,GSC2,GSX1,GSX2,GUCY1A3,GUCY2D,HAND2,HBA1,HBA2,HES2,HES7,HEY1,HHAT,HHEX,HHIP,HLX,MNX1,HMX2,HMX3,HOXB1,HOXB13,HOXB2,HOXB3,HOXB6,HOXB7,HOXB8,HOXC11,HOXC12,HOXC4,HOXC5,HOXC6,HOXC8,HOXD1,HOXD12,HOXD13,HOXD3,HOXD4,HOXD8,HOXD9,HPCAL4,HPSE2,HRK,HS3ST3B1,HS6ST1P,HS6ST3,HSF4,HSPA6,HTR1A,HTR2C,HTR7,ICAM5,IGF2AS,IGSF21,IL1RAPL2,IL7,INA,INSM2,INSRR,PDX1,IRX3,IRX4,IRX5,ISL1,ISL2,ITGA4,ITPKA,JUN,KAZALD1,KCNA1,KCNA3,KCNAB1,KCNC2,KCNC4,KCND3,KCNH1,KCNH3,KCNK12,KCNK13,KCNK2,KCNK4,KCNMA1,KCNQ3,KCNV1,VASH1,KIAA1199,KIAA1324,KIAA1666,KIRREL3,KL,KLF4,KY,LBX1,LGALS3,LGR5,LHX2,LHX4,LHX5,LHX6,LHX8,LMX1B,TMEM132E,C1orf194,LAYN,C1orf213,LOC150221,LOC153684,NBPF11,RBM32A,RPRML,C17orf82,C5orf39,LOC400120,DUOXA2,LOC440804,LOC441413,ANKRD20A3,LOC441426,ANKRD20A2,ANKRD18B,NDUFA4L2,TMEM88,LPHN3,LPL,LRCH2,LRFN5,LRP2,LRRTM1,LTBP2,LTK,LYSMD2,MAB21L1,MAB21L2,MAFB,MAL,MAPK4,MAPT,MCOLN3,MESP1,METRNL,MAG1,FAM81A,MGC26718,RSPO2,MGC39545,MLLT3,MSC,MSX1,MT1A,MT1B,MT1H,MT1M,MT1DP,MYF6,MYO5B,MYOD1,NAGS,NAV2,NCAM1,NEFM,NEFL,NELL1,NEUROD1,NEUROD2,NEUROG1,NEUROG2,NEUROG3,NFIX,DUOXA1,NKX2-2,NKX2-3,NKX2-8,NKX3-1,NKX6-1,NKX6-2,NLF1,NOL4,NPAS1,NPNT,NPR3,NPTX1,NPY1R,NR2F2,NR4A3,NRG1,NRG2,OAF,NT5C1A,NTN1,NTNG2,NTRK1,NTRK2,NPAS4,OCA2,OLFML2B,OLIG2,ONECUT1,ONECUT2,OPRD1,OSAP,OSR1,OTOP1,OTOP2,OTOP3,OTP,OTX1,OTX2,OXCT2,PAPPA,PAX1,PAX2,PAX3,PAX6,PAX7,PAX8,PAX9,PCDH17,PCDH8,PDE4DIP,PDGFRA,FRMPD4,PDZD2,PENK,PGM5,PGR,PHOX2A,PHOX2B,PIP5K1B,PIR,PITX1,PITX2,PITX3,PKNOX2,PKP1,PLEC1,PLXNA2,PMP22,PODN,POLE,POU3F1,POU3F4,POU4F1,POU4F2,POU4F3,PPM1E,PRAC,PRDM12,RP11-35N6.1,PRKCE,PRKG1,PROK2,PTF1A,PTGDR,PTGER2,PTGER3,PTGER4,PTGFR,PTHLH,PTPRT,PTPRU,PXMP2,PYY,RAB6C,SHC4,RASGRF1,RASSF5,RAX,RBP4,REPS2,C13orf15,RGS10,RGS20,RGS9BP,RIPK3,LONRF3,RNF128,ROBO3,RPS6KA6,RASL10A,RSPO1,RTN4RL2,RYR3,SCD5,SCN4B,SCNN1G,SCTR,SEMA6D,SFRP1,SFRP5,SGPP2,SHH,SHOX,SHOX2,SIDT1,SIM2,SIX1,SIX2,SIX3,SIX6,SLC10A4,SLC1A2,SLC1A4,SLC24A4,SLC26A4,SLC27A2,SLC30A2,SLC30A3,SLC30A4,SLC32A1,SLC35F3,SLC6A1,SLC6A3,SLC6A5,SLC9A2,SLC9A3,SLCO2A1,SLCO5A1,SLIT1,SLIT2,SLITRK1,SLITRK3,PIGZ,BATF3,SORCS1,SORCS3,SOX14,SOX17,SOX7,SPAG6,SPOCK3,SPON1,SRD5A2,SSTR1,SSTR2,ST8SIA2,STK32B,STMN2,STXBP6,SUSD4,SV2B,SYT12,TAL1,TBR1,TBX1,TBX2,TBX21,TBX3,TBX5,TCEA3,HNF1B,TFAP2E,THBD,TIP39,NKX2-1,TLL1,TLX1,TLX2,TMEFF2,TMEM27,TMEM30B,TMOD2,CD70,TP73,TRADD,TRH,TRIM36,TRIM67,TRIM9,TRPC5,TSLP,TTYH1,UCN,UCP1,UNC5C,UNQ9433,USH1G,VAX1,VAX2,VDR,VSX1,WDR8,WIT1,WNT1,WNT10A,WNT10B,WNT11,WNT16,WNT2,WNT3A,WNT6,WNT7A,WT1,ZADH2,ZBTB16,ZCCHC16,ZEB2,ZFYVE28,ZIC1,ZIC4,ZMYND15,FEZF2,ZNF436,ZNF503,IKZF3,

Myc.targets1 CDK4,TIMM17B,HRSP12,OPRS1,TCIRG1,AKR1A1,CFDP1,CEACAM5,CEBPA,RIG,PAICS,KLF1,EBP,ATF7,RCC1,PIM2,CHRNB1,CLCN6,CLNS1A,HIST4H4,PARP1,CSTB,DBI,DCK,ACE,DKC1,E2F1,EEF1A2,EGR3,EIF4A1,EIF4E,ELK1,ENO1,ETS2,ALDH2,FAH,FASN,FGFR4,FOXM1,SIRT1,RPL13A,FPGS,FXN,GALNS,GALT,GAPDH,AMD1,GAS8,SNORD4A,SNORD32A,PABPC1,AMPD2,BBC3,GLA,AMPD3,GPR4,GSK3B,GSTP1,H2AFZ,HBA2,HBAP1,HBB,HBE1,HIF1A,MNX1,HMBS,HMGN2,HNRNPA1,HNRNPA2B1,APC,APEX1,HES1,HSD11B2,HSPA8,HSP90AA1,HSP90AB1,HSPD1,ICAM1,ID3,ACADM,APP,IL11RA,IMPA2,INSR,IRF2,IRF3,ISG20,ITGB1,JUN,JUNB,ARF1,ARF4,SNHG5,RHOG,STMN1,LBR,LMNA,SMAD7,MAGEA3,MAGEA6,ARSB,MAN2A2,MCM4,MCM7,MDM2,MET,MFI2,MGST1,ASCL2,MPO,MSN,MST1,MTHFR,MUC1,MYCL1,ATF4,NBN,NCL,NEFM,NIT1,NME1,NOL1,NOTCH4,NTHL1,NTN2L,OCA2,ACO2,SERPINE1,ACOX1,GOLGA7,PDCD1,PDHA1,PER1,PHB,PLA2G4A,PLK1,POLB,POLD2,PPID,NSUN2,AVP,PPP1R7,HIST2H4B,PRTN3,PSEN2,EIF5A2,PSMA3,PSMB1,PTEN,PTGER2,PTMA,BAX,QDPR,RARB,RBM3,CCND1,BCL2,RGS2,ACTB,BCL3,RPL10,RPL19,RPL22,RPL23A,RPL27A,RPL36A,RPS6,RPS15,RPS19,RXRB,BLK,SLC2A4,BMP4,SLC4A2,SLC19A1,POLR3D,SRD5A1,SRM,BRCA2,STAT6,ZFP36L2,BSG,SURF6,VAMP2,TCF12,TERT,TFRC,TGFB1,TGFB3,ACTG1,TOP1,TPM2,TSC2,TXN,UBA52,UCP3,UROD,VHL,WEE1,WT1,ZNF146,IFRD2,SLBP,FOSL1,AKAP1,BCL2L12,HIST1H4E,CASP8,LMNB2,PEX3,LOC85389,CDK10,CTSF,CBS,HDAC3,CCKBR,SQSTM1,CCNB1,CCND2,CD2,SYNGR2,AP4M1,SLC9A3R2,TNFRSF8,NR1D1,CD63,HERPUD1,CD79B,TOMM20,HNRPDL

Myc.targets2 AMD1,ARL1,ARRB2,ASAH1,ATF4,ATM,ATP5B,ATP5C1,ATP5E,ATP5G2,BARD1,BCKDHA,BCKDHB,BLK,CACNB1,CAD,CALM2,CALR,CAPZA1,CD79B,CD97,CDC6,CDC25B,CDK6,CDKN1B,CTSC,CISH,CKS2,CLCN2,CLCN3,CLCN6,CLN3,SLC31A1,COX7C,COX15,CR2,CREB1,CREBL2,CSF2RB,CSTF3,CYBA,DAXX,DBI,DBP,DDB1,DDX1,DDX3X,DDX5,DDX10,DPAGT1,DPYSL3,EIF2S1,EIF2S3,ENO2,STOM,ERCC6,ETF1,ETFA,FABP5,FANCF,FKBP2,FLOT2,FRAP1,FTH1,XRCC6,GABPA,GAK,GALC,NR6A1,GCLC,GMDS,GNA12,GNL1,GNS,GRK4,H2AFX,H2AFZ,HADHB,HARS,HIP2,HLA-A,HLA-F,HMMR,HNRNPA1,HNRNPL,HSPA2,HSPA9,HSPE1,IDH3B,IFNAR1,IFNGR1,IGBP1,ILK,ING1,IRF3,JARID2,KHK,KIF11,KPNA3,RANBP5,LAMP1,LDHA,FADS3,LNPEP,LSP1,LTA4H,M6PR,NBR1,MAD2L1,SMAD3,MAGOH,MAN2A1,MARS,MAT2A,MBNL1,MCM3,MCM5,MDH1,MAP3K5,RAB8A,METTL1,MFAP1,MFNG,MGAT2,MKLN1,MMP8,MMP15,MOCS2,MSH2,MST1R,MT2A,MT3,MTF1,MTHFD1,PPP1R12A,NACA,NAGA,NUBP1,NCBP1,NCL,NDUFA1,NDUFA2,NDUFA6,NDUFB1,NDUFB2,NDUFB3,NDUFB4,NDUFB5,NDUFB6,NDUFS1,NDUFV1,NDUFS6,DRG1,NFKBIB,NME1,CNOT2,CNOT4,NUP88,OAZ1,PA2G4,PCM1,PCMT1,PCNA,PCTK1,PCYT1A,PDE4C,PDE6D,PDK1,PDK2,PDK3,SLC26A4,PER1,PET112L,PEX6,PFKFB4,PFN1,PIGF,PKM2,PMM2,PMS1,PMS2L3,UBL3,POLH,POU2F1,PPA1,PPIA,PPP1CB,PPP1R7,PPP2CA,PPP2R1B,PPP2R4,PPP3CA,PREP,PRKAB1,PRKAB2,PRKAG1,PKN2,PRKCSH,PRKDC,MAPK7,MAP2K5,MAP2K7,PRKRIR,PRPS2,PRPSAP1,PSMA1,PSMA5,PSMB1,PSMB5,PSMB7,PSMC4,PSMC5,PSMD3,PSMD5,PSMD7,PSMD8,PSMD10,PTPN1,PTPN6,PTPRF,PYCR1,ALDH18A1,RAB1A,RGL2,RAB3A,RAC2,RAD9A,RAP2B,RASA1,ARID4A,JARID1A,RBBP8,RBL1,RBM4,RECQL,RFX5,RGS16,RHEB,RLN1,RNF4,ABCE1,RPL5,RPL8,RPL9,RPL13,RPL15,RPL18,RPL19,RPL27,RPL27A,RPL31,RPL32,RPL37,RPLP1,RPS5,RPS6,RPS6KA2,RPS6KB1,RPS13,RPS17,RPS19,RPS20,RPS21,RPS25,RPS26,RPS27A,RPS29,RRAS,RXRB,VPS52,SARS,SCD,SCO1,SDF2,SFRS2,SFRS5,SFRS7,SLC1A4,SLC2A4,SLC7A1,SLC12A2,SLC22A3,SLC22A4,SNAPC1,SNAPC3,SNRPA,SNRPD3,SOD1,SOX12,SP4,SPIB,SRD5A1,SRI,SREBF2,SRP54,SRP68,SRPR,SSB,SUPT5H,VAMP1,SYK,SYPL1,CNTN2,TCEB3,C2orf3,TCF12,MLX,TLE3,TLL2,TLOC1,TMF1,TMPO,TMSB4X,TNFAIP1,TP53,TPP2,TPR,TPT1,HSP90B1,TSC2,TSPY1,TTC4,TUBG1,TULP3,TYK2,U2AF1,UBE2D3,UBE2G2,UCHL1,USP4,UQCRC2,USP1,VASP,VCP,VDAC2,VGF,EIF4H,XK,YY1,YWHAH,ZFPL1,ZNF7,ZNF12,ZNF35,ZNF85,ZNF134,ZNF136,ZNF142,TRIM25,ZNF174,ZNF192,ZNF225,PTP4A1,CSDE1,DAP3,FZD5,ARMET,SLC39A7,EPM2A,JTV1,UBXD6,GLRA3,CCDC6,CUL5,COIL,ELL,DPF1,DGCR6,LZTR1,HDHD1A,USP11,JARID1C,SMC1A,TMEM187,LAGE3,HIST1H4I,ACOX3,HIST1H2AI,HIST1H2AK,HIST1H2AJ,HIST1H2AC,HIST1H2AM,HIST2H2AA3,HIST1H2BG,HIST1H2BN,HIST1H2BM,HIST1H2BF,HIST1H2BE,HIST1H2BH,HIST1H2BI,HIST1H2BC,HIST2H2BE,HIST1H4B,HIST1H4L,PLA2G6,SLC25A11,TAGLN2,UXT,EEA1,STX7,CMAH,RAD54L,GNPAT,SIP1,RANBP3,SLC43A1,PEX3,PARG,CSDA,COPS3,AGPS,BLZF1,PIAS1,DEGS1,MADD,MKNK1,KHSRP,AKR7A2,PRKRA,USO1,DSCR2,RNASET2,EIF3I,STX16,STX10,PLA2G4B,JRKL,SNX3,CRADD,CD164,RAB11A,RIPK2,PEX11B,SUCLG1,TRIM24,DPM1,DPM2,SAP30,GGH,ALKBH1,DLEU2,ARHGEF7,SQSTM1,SGPL1,CDC16,ZNF259,SLC5A6,HERC1,MBD4,HIST1H2AG,HIST1H2BJ,PLOD3,CH25H,BAZ1B,NFS1,ASH2L,LATS1,RQCD1,AIFM1,RRP9,ATG12,EXO1,COX7A2L,AP4M1,VAPA,RPS6KA5,PSCD2,WDR46,SFRS11,COPS2,TRIP13,GTF3C5,GTF3C4,EFTUD2,PPT2,CIAO1,RECQL5,PEX16,MED17,GSTO1,LY86,EIF4E2,MED20,PMPCB,FXR2,SPTLC2,EEF1E1,POLR1C,CIR,APBA3,H2AFY,CHD1L,H6PD,NR1D1,SPAG6,RBM39,MPHOSPH1,IER2,PSCDBP,RNF7,PTDSS1,MAML1,KEAP1,GINS1,AMMECR1,GOLGA5,HS3ST2,THRAP3,NUP153,DMTF1,HDAC6,SH2D3C,SAE1,ABCB6,SCAMP3,ARPC4,ACTR3,NUBP2,RAD50,KIF20A,FRY,TRAP1,ARFRP1,AKAP9,CEBPZ,RCL1,PSME3,MPHOSPH6,USPL1,PSMD14,TRIB1,NBR2,HRSP12,ABCC4,ZMPSTE24,STUB1,OPRS1,SDCCAG10,SAP18,DNAJA2,MAEA,DLEU1,LANCL1,TMEM5,TMEM4,HMG20A,CACNG3,TUBA1B,SCML2,CEPT1,ANAPC10,PIAS3,PRMT5,TESK2,TIMM23,IFI30,TIMM17A,HAX1,COG5,TIMM44,MYBBP1A,CIB1,DDX17,HBXIP,ARL6IP5,CCT7,ERLIN1,SPAG5,POLR3G,IVNS1ABP,RAD51AP1,TUSC4,PMVK,RRAGA,CCT8,AP4B1,MGEA5,NFAT5,PTGES3,AHCYL1,ARPP-19,ZNF274,WDR4,YIF1A,SUGT1,RNPS1,SEC61B,TMED10,MAPRE2,CYB561D2,TOPBP1,HSF2BP,HNRPUL1,BTN2A1,POLR3A,PWP1,AP4S1,NUDT6,MAP4K5,RASSF1,SUPT16H,AKAP10,RPL35,PDCD10,DCTN3,PHB2,GABARAP,MTF2,NCBP2,MAPRE1,ATF6,KCNH4,ITGB3BP,AP4E1,ABCB10,CBX5,TTC33,DDX58,CORO1C,CD2AP,MKRN1,NUP62,ARFIP2,LYPLA3,ZKSCAN5,CARKL,SDF2L1,MAFF,FTSJ1,RAB3GAP2,SLC39A6,ATXN10,PRDX5,APPL1,FBXO5,MYCBP,TIMM9,TIMM8B,NUFIP1,DNAI1,ACAD8,ST6GALNAC4,TRAPPC3,CACYBP,ATP5S,SNX5,IL17C,SERP1,ARHGEF16,TNFRSF21,LSM1,MOCS3,EIF3K,RABGEF1,HTRA2,MAT2B,NXT1,TMOD2,ABT1,UCRC,RBM15B,CPSF1,SNX15,HOOK2,HCFC2,PYCR2,TIMM22,GPR132,SERTAD1,POMT2,UBQLN1,ERO1L,CXXC1,ZNRD1,DEF6,WDR42A,RNF141,HEBP1,LOC51035,ZNF593,RPS27L,POLR1D,CCDC41,LOC51136,DYNC1LI1,MRTO4,DCTN4,NAGPA,HSPA14,ATPBD1C,C15orf15,ABI3,PIGP,CDKL3,IER5,SCAND1,GMIP,FKBP11,ZDHHC3,ATP6V1D,PPME1,NOL7,DDX41,HSD17B7,SCLY,MIR16,AZIN1,PCF11,TRIM33,CUTA,LSR,LIPT1,KIAA0859,TRMT6,STYXL1,GINS2,BRP44L,ASB1,MPP6,LSM8,VPS29,PPP6C,POLR3K,RAPGEF6,RP6-213H19.1,ZAK,BTBD1,NUP54,ADAM22,PTOV1,DSCR6,POLE3,H2BFS,GDAP1,CCDC76,GNB1L,LZTFL1,TRIM44,OCIAD1,CNDP2,GLT8D1,CBWD1,ACSS2,DMAP1,C20orf24,SLC25A40,DIABLO,SERF1B,PNO1,KIF15,ENTPD7,PCNP,ZNF286A,BIRC6,POLD4,ZNF410,NGB,ALOXE3,TGIF2,SAV1,GPSM3,MCCC2,MAGEF1,SLC39A8,NCAPG,LEPRE1,ELOVL1,MRPL40,TSEN34,EPC1,ULBP1,WDR23,PRR3,POLR1B,WBSCR22

**Table S4**: Gene-Set-Enrichment p-values for enrichment in stem-like and differentiated subtypes for all of the curated gene sets on MSigDB v 3.0. No multiple testing correction was used. The complete table with p-values for all genesets tested is available on <http://bcb.dfci.harvard.edu/~aedin/publications/>

| **MSigDB gene signature** | **Differentiated** | **Stem-like** |
| --- | --- | --- |
| AMUNDSON POOR SURVIVAL AFTER GAMMA RADIATION 2G | 1 | 0 |
| BYSTRYKH HEMATOPOIESIS STEM CELL FLI1 | 1 | 0 |
| CHANG CORE SERUM RESPONSE UP | 1 | 0 |
| CHIARADONNA NEOPLASTIC TRANSFORMATION KRAS UP | 1 | 0 |
| COLLER MYC TARGETS UP | 1 | 0 |
| CREIGHTON ENDOCRINE THERAPY RESISTANCE 1 | 1 | 0 |
| DODD NASOPHARYNGEAL CARCINOMA DN | 1 | 0 |
| HOFMANN CELL LYMPHOMA UP | 1 | 0 |
| KYNG DNA DAMAGE BY UV | 1 | 0 |
| LEE TARGETS OF PTCH1 AND SUFU DN | 1 | 0 |
| MEINHOLD OVARIAN CANCER LOW GRADE DN | 1 | 0 |
| NUNODA RESPONSE TO DASATINIB IMATINIB UP | 1 | 0 |
| PENG LEUCINE DEPRIVATION DN | 1 | 0 |
| PODAR RESPONSE TO ADAPHOSTIN DN | 1 | 0 |
| RHODES CANCER META SIGNATURE | 1 | 0 |
| RUGO UV RESPONSE | 1 | 0 |
| SENGUPTA NASOPHARYNGEAL CARCINOMA UP | 1 | 0 |
| TONKS TARGETS OF RUNX1 RUNX1T1 FUSION MONOCYTE UP | 1 | 0 |
| YE METASTATIC LIVER CANCER | 1 | 0 |
| BENPORATH ES CORE NINE CORRELATED | 0.99999 | 1.00E-05 |
| CHANDRAN METASTASIS UP | 0.99999 | 1.00E-05 |
| LEE LIVER CANCER SURVIVAL DN | 0.99999 | 1.00E-05 |
| LIANG SILENCED BY METHYLATION DN | 0.99999 | 1.00E-05 |
| LY AGING OLD DN | 0.99999 | 1.00E-05 |
| OLSSON E2F3 TARGETS DN | 0.99999 | 1.00E-05 |
| OUELLET OVARIAN CANCER INVASIVE VS LMP UP | 0.99999 | 1.00E-05 |
| REACTOME EARLY PHASE OF HIV LIFE CYCLE | 0.99999 | 1.00E-05 |
| SHEPARD BMYB MORPHOLINO DN | 0.99999 | 1.00E-05 |
| SUNG METASTASIS STROMA DN | 0.99999 | 1.00E-05 |
| TANG SENESCENCE TP53 TARGETS DN | 0.99999 | 1.00E-05 |
| TSENG IRS1 TARGETS UP | 0.99999 | 1.00E-05 |
| BHATTACHARYA EMBRYONIC STEM CELL | 0.99998 | 2.00E-05 |
| CHIARETTI T ALL RELAPSE PROGNOSIS | 0.99998 | 2.00E-05 |
| GRAHAM CML QUIESCENT VS NORMAL QUIESCENT UP | 0.99998 | 2.00E-05 |
| HEDENFALK BREAST CANCER HEREDITARY VS SPORADIC | 0.99998 | 2.00E-05 |
| LY AGING MIDDLE DN | 0.99998 | 2.00E-05 |
| MUNSHI MULTIPLE MYELOMA DN | 0.99998 | 2.00E-05 |
| NIELSEN LIPOSARCOMA DN | 0.99998 | 2.00E-05 |
| SCIAN CELL CYCLE TARGETS OF TP53 AND TP73 DN | 0.99998 | 2.00E-05 |
| ST T CELL SIGNAL TRANSDUCTION | 0.99998 | 2.00E-05 |
| VANTVEER BREAST CANCER METASTASIS DN | 0.99998 | 2.00E-05 |
| VANTVEER BREAST CANCER POOR PROGNOSIS | 0.99998 | 2.00E-05 |
| CHIANG LIVER CANCER SUBCLASS PROLIFERATION UP | 0.99997 | 3.00E-05 |
| MCCOLLUM GELDANAMYCIN RESISTANCE DN | 0.99997 | 3.00E-05 |
| PUJANA BREAST CANCER LIT INT NETWORK | 0.99997 | 3.00E-05 |
| RASHI RESPONSE TO IONIZING RADIATION 4 | 0.99997 | 3.00E-05 |
| CHEMNITZ RESPONSE TO PROSTAGLANDIN E2 UP | 0.99996 | 4.00E-05 |
| CHIN BREAST CANCER COPY NUMBER UP | 0.99996 | 4.00E-05 |
| CROONQUIST NRAS VS STROMAL STIMULATION DN | 0.99996 | 4.00E-05 |
| GRASEMANN RETINOBLASTOMA WITH 6P AMPLIFICATION | 0.99996 | 4.00E-05 |
| KARAKAS TGFB1 SIGNALING | 0.99996 | 4.00E-05 |
| KORKOLA EMBRYONAL CARCINOMA | 0.99996 | 4.00E-05 |
| LY AGING PREMATURE DN | 0.99996 | 4.00E-05 |
| VANTVEER BREAST CANCER ESR1 DN | 0.99996 | 4.00E-05 |
| BERENJENO TRANSFORMED BY RHOA UP | 0.99995 | 5.00E-05 |
| CAIRO HEPATOBLASTOMA POOR SURVIVAL | 0.99995 | 5.00E-05 |
| CHIARETTI T ALL REFRACTORY TO THERAPY | 0.99995 | 5.00E-05 |
| MOREAUX B LYMPHOCYTE MATURATION BY TACI DN | 0.99995 | 5.00E-05 |
| MORI SMALL PRE BII LYMPHOCYTE DN | 0.99995 | 5.00E-05 |
| PUJANA ATM PCC NETWORK | 0.99995 | 5.00E-05 |
| PUJANA BREAST CANCER WITH BRCA1 MUTATED UP | 0.99995 | 5.00E-05 |
| REACTOME GLUCOSE TRANSPORT | 0.99995 | 5.00E-05 |
| RHODES UNDIFFERENTIATED CANCER | 0.99995 | 5.00E-05 |
| SMID BREAST CANCER LUMINAL A DN | 0.99995 | 5.00E-05 |
| SMID BREAST CANCER RELAPSE IN BRAIN UP | 0.99995 | 5.00E-05 |
| GLINSKY CANCER DEATH UP | 0.99994 | 6.00E-05 |
| GRAHAM CML QUIESCENT VS CML DIVIDING DN | 0.99994 | 6.00E-05 |
| FLOTHO PEDIATRIC ALL THERAPY RESPONSE DN | 0.99993 | 7.00E-05 |
| KENNY CTNNB1 TARGETS UP | 0.99993 | 7.00E-05 |
| SMID BREAST CANCER BASAL UP | 0.99993 | 7.00E-05 |
| YAGI AML SURVIVAL | 0.99993 | 7.00E-05 |
| ABRAHAM ALPC VS MULTIPLE MYELOMA DN | 0.99992 | 8.00E-05 |
| BENPORATH ES 1 | 0.99992 | 8.00E-05 |
| FURUKAWA DUSP6 TARGETS PCI35 DN | 0.99992 | 8.00E-05 |
| SCHUHMACHER MYC TARGETS UP | 0.99992 | 8.00E-05 |
| SONG TARGETS OF IE86 CMV PROTEIN | 0.99992 | 8.00E-05 |
| ZHAN EARLY DIFFERENTIATION GENES DN | 0.99992 | 8.00E-05 |
| MITSIADES RESPONSE TO APLIDIN DN | 0.99991 | 9.00E-05 |
| NADERI BREAST CANCER PROGNOSIS UP | 0.99991 | 9.00E-05 |
| REACTOME SHC MEDIATED SIGNALLING | 0.99991 | 9.00E-05 |
| BIOCARTA SET PATHWAY | 0.9999 | 1.00E-04 |
| CHANG CYCLING GENES | 0.9999 | 1.00E-04 |
| FOURNIER ACINAR DEVELOPMENT LATE 2 | 0.9999 | 1.00E-04 |
| MARKEY RB1 ACUTE LOF DN | 0.9999 | 1.00E-04 |
| SASAKI ADULT T CELL LEUKEMIA | 0.9999 | 1.00E-04 |
| TARTE PLASMA CELL VS PLASMABLAST DN | 0.9999 | 1.00E-04 |
| SCHWAB TARGETS OF BMYB I624M DN | 1.00E-04 | 0.9999 |
| SCHWAB TARGETS OF BMYB S427G DN | 1.00E-04 | 0.9999 |
| VECCHI GASTRIC CANCER ADVANCED VS EARLY DN | 1.00E-04 | 0.9999 |
| DAVICIONI MOLECULAR ARMS VS ERMS UP | 7.00E-05 | 0.99993 |
| REACTOME TRIACYLGLYCERIDE BIOSYNTHESIS | 5.00E-05 | 0.99995 |
| YANG BREAST CANCER ESR1 LASER UP | 5.00E-05 | 0.99995 |
| FINETTI BREAST CANCERS KINOME BLUE | 4.00E-05 | 0.99996 |
| KEGG PEROXISOME | 4.00E-05 | 0.99996 |
| FLECHNER BIOPSY KIDNEY TRANSPLANT REJECTED VS OK DN | 3.00E-05 | 0.99997 |
| OUELLET OVARIAN CANCER INVASIVE VS LMP DN | 3.00E-05 | 0.99997 |
| REACTOME GLUTATHIONE CONJUGATION | 1.00E-05 | 0.99999 |
| WEBER METHYLATED HCP IN FIBROBLAST UP | 1.00E-05 | 0.99999 |
| CHEMNITZ RESPONSE TO PROSTAGLANDIN E2 DN | 0 | 1 |
| CONRAD GERMLINE STEM CELL | 0 | 1 |
| CREIGHTON ENDOCRINE THERAPY RESISTANCE 2 | 0 | 1 |
| DOANE BREAST CANCER CLASSES UP | 0 | 1 |
| DOANE BREAST CANCER ESR1 UP | 0 | 1 |
| DODD NASOPHARYNGEAL CARCINOMA UP | 0 | 1 |
| LIEN BREAST CARCINOMA METAPLASTIC VS DUCTAL DN | 0 | 1 |
| MEINHOLD OVARIAN CANCER LOW GRADE UP | 0 | 1 |
| NAKAMURA BRONCHIAL AND BRONCHIOLAR EPITHELIA | 0 | 1 |
| SENGUPTA NASOPHARYNGEAL CARCINOMA DN | 0 | 1 |
| SMID BREAST CANCER BASAL DN | 0 | 1 |
| SMID BREAST CANCER LUMINAL B UP | 0 | 1 |
| SMID BREAST CANCER RELAPSE IN BONE UP | 0 | 1 |
| SMID BREAST CANCER RELAPSE IN BRAIN DN | 0 | 1 |
| VANTVEER BREAST CANCER ESR1 UP | 0 | 1 |
| YANG BREAST CANCER ESR1 BULK UP | 0 | 1 |

**Table S5**: Gene-Set-Enrichment p-values for enrichment in stem-like and differentiated subtypes for all of the Gene Ontology gene sets on MSigDB v 3.0. No multiple testing correction was used. The complete table with p-values for all genesets tested is available on <http://bcb.dfci.harvard.edu/~aedin/publications/>

| **MSigDB gene signature** | **Differentiated** | **Stem-like** |
| --- | --- | --- |
| NUCLEAR TRANSPORT | 1 | 0 |
| NEGATIVE REGULATION OF IMMUNE SYSTEM PROCESS | 1 | 0 |
| MITOTIC SPINDLE ORGANIZATION AND BIOGENESIS | 1 | 0 |
| NUCLEOCYTOPLASMIC TRANSPORT | 1 | 0 |
| TRANSCRIPTION COREPRESSOR ACTIVITY | 1 | 0 |
| KINESIN COMPLEX | 0.99999 | 1.00E-05 |
| VIRAL GENOME REPLICATION | 0.99999 | 1.00E-05 |
| VIRAL INFECTIOUS CYCLE | 0.99998 | 2.00E-05 |
| VIRAL REPRODUCTIVE PROCESS | 0.99998 | 2.00E-05 |
| PROTEIN IMPORT INTO NUCLEUS | 0.99998 | 2.00E-05 |
| MITOTIC SISTER CHROMATID SEGREGATION | 0.99997 | 3.00E-05 |
| SISTER CHROMATID SEGREGATION | 0.99997 | 3.00E-05 |
| CONDENSED CHROMOSOME | 0.99996 | 4.00E-05 |
| NUCLEAR IMPORT | 0.99996 | 4.00E-05 |
| CELL DIVISION | 0.99994 | 6.00E-05 |
| RHO GUANYL NUCLEOTIDE EXCHANGE FACTOR ACTIVITY | 0.99994 | 6.00E-05 |
| GTP BINDING | 0.99992 | 8.00E-05 |
| GUANYL NUCLEOTIDE BINDING | 0.99992 | 8.00E-05 |
| ENDODEOXYRIBONUCLEASE ACTIVITY | 0.99991 | 9.00E-05 |
| ENDONUCLEASE ACTIVITY | 0.99991 | 9.00E-05 |
| CHROMOSOME PERICENTRIC REGION | 0.9999 | 1.00E-04 |
| REGULATION OF CELL CYCLE | 0.9999 | 1.00E-04 |
| NITROGEN COMPOUND CATABOLIC PROCESS | 1.00E-04 | 0.9999 |
| AMINE CATABOLIC PROCESS | 8.00E-05 | 0.99992 |
| COFACTOR TRANSPORTER ACTIVITY | 6.00E-05 | 0.99994 |
| APICAL PART OF CELL | 5.00E-05 | 0.99995 |
| AMINO ACID CATABOLIC PROCESS | 5.00E-05 | 0.99995 |
| OXIDOREDUCTASE ACTIVITY ACTING ON PEROXIDE AS ACCEPTOR | 3.00E-05 | 0.99997 |
| APICAL PLASMA MEMBRANE | 2.00E-05 | 0.99998 |
| CELL PROJECTION PART | 0 | 1 |

**Table S6**: Genes over-expressed in breast cancer cells containing wildtype and mutant p53 from Fig. 5 of Troester et al. [[42](#_ENREF_42)]

| **Gene signature** | **Gene** | **Over-expressed in WT or mutant p53 cells** | **Over-expressed in stem-like or differentiated subtype** | **P-value** |
| --- | --- | --- | --- | --- |
| **Troester et al.** | MAP2K4 | WT | Differentiated | 0.002 |
|  | GATA3 | WT | Differentiated | 0.03 |
|  | NPEPPS | WT | Differentiated | 0.002 |
|  | KIAA1370 | WT | Differentiated | 0.244 |
|  | FAM198B | WT | Stem-like | 0.554 |
|  | FAM174A | WT | Differentiated | < 0.001 |
|  | KIAA0040 | WT | Differentiated | 0.334 |
|  | CDKN1A | WT | Differentiated | < 0.001 |
|  | CCND1 | WT | Differentiated | 0.333 |
|  | NEO1 | WT | Stem-like | 0.025 |
|  | RERG | WT | Stem-like | 0.173 |
|  | APH1B | WT | Differentiated | 0.702 |
|  | FNBP1 | WT | Differentiated | 0.116 |
|  | DDB2 | WT | Differentiated | 0.073 |
|  | TC2N | WT | Differentiated | 0.438 |
|  | BTG2 | WT | Differentiated | 0.749 |
|  | SLC39A6 | WT | Differentiated | 0.277 |
|  | TCEAL1 | WT | Differentiated | 0.038 |
|  | TUBA3C | WT | Stem-like | 0.304 |
|  | CEP55 | Mutant | Stem-like | < 0.001 |
|  | CDC25B | Mutant | Stem-like | < 0.001 |
|  | TOP2A | Mutant | Stem-like | 0.006 |
|  | MCM3 | Mutant | Stem-like | 0.08 |
|  | MYBL2 | Mutant | Stem-like | < 0.001 |
|  | SCO2 | Mutant | Stem-like | 0.46 |
|  | CHMP4C | Mutant | Differentiated | 0.157 |
|  | KIFC1 | Mutant | Stem-like | 0.087 |
|  | AURKA | Mutant | Stem-like | 0.013 |
|  | KIF23 | Mutant | Stem-like | < 0.001 |
|  | POLD1 | Mutant | Stem-like | 0.012 |
|  | ATAD2 | Mutant | Stem-like | < 0.001 |
|  | CDK1 | Mutant | Stem-like | 0.005 |
|  | PTTG1 | Mutant | Stem-like | 0.012 |
|  | RFC4 | Mutant | Stem-like | 0.006 |
|  | CDCA7L | Mutant | Stem-like | 0.012 |
|  | TAP1 | Mutant | Stem-like | 0.175 |
|  | NUDT1 | Mutant | Stem-like | 0.124 |
|  | PREP | Mutant | Stem-like | 0.985 |
|  | TRIP13 | Mutant | Stem-like | < 0.001 |
|  | C21orf45 | Mutant | Stem-like | < 0.001 |
|  | FOXM1 | Mutant | Stem-like | < 0.001 |
|  | CDC25C | Mutant | Stem-like | 0.826 |
|  | TUBA4A | Mutant | Stem-like | 0.081 |
|  | CENPF | Mutant | Stem-like | < 0.001 |
|  | GGH | Mutant | Stem-like | 0.532 |
|  | MAD2L1 | Mutant | Stem-like | 0.006 |
|  | MKI67 | Mutant | Stem-like | < 0.001 |
|  | CDKN3 | Mutant | Stem-like | 0.002 |
|  | CCNA2 | Mutant | Stem-like | 0.001 |
|  | UBE2C | Mutant | Stem-like | < 0.001 |

| **Gene signature** | **Gene** | **Over-expressed in WT or mutant p53 cells** | **Over-expressed in stem-like or differentiated subtype** | **P-value** |
| --- | --- | --- | --- | --- |
| **Takahashi et al.** | PTTG1 | WT | Differentiated | 0.373 |
|  | PLK1 | WT | Differentiated | 0.015 |
|  | UBE2C | WT | Stem-like | 0.165 |
|  | LIN9 | WT | Stem-like | 0.763 |
|  | PTP4A2 | WT | Differentiated | 0.066 |
|  | RPS27L | WT | Differentiated | 0.45 |
|  | HEXIM1 | WT | Differentiated | 0.017 |
|  | SULF2 | WT | Differentiated | < 0.001 |
|  | PRC1 | WT | Differentiated | 0.497 |
|  | ASPM | Mutant | Stem-like | 0.007 |
|  | UBE2T | Mutant | Stem-like | < 0.001 |
|  | C3orf18 | Mutant | Stem-like | 0.321 |
|  | BIRC5 | Mutant | Stem-like | 0.001 |
|  | CDC45 | Mutant | Stem-like | 0.004 |
|  | MKNK2 | Mutant | Stem-like | < 0.001 |
|  | MAPRE1 | Mutant | Stem-like | 0.009 |
|  | STMN1 | Mutant | Stem-like | 0.001 |
|  | CENPF | Mutant | Stem-like | 0.001 |
|  | BCL11A | Mutant | Stem-like | 0.012 |
|  | PKMYT1 | Mutant | Stem-like | 0.037 |
|  | MUTYH | Mutant | Stem-like | 0.002 |
|  | CDCA8 | Mutant | Stem-like | 0.003 |
|  | KIF23 | Mutant | Stem-like | < 0.001 |
|  | CEP55 | Mutant | Stem-like | 0.01 |
|  | CENPE | Mutant | Stem-like | < 0.001 |
|  | FANCI | Mutant | Stem-like | 0.002 |
|  | C15orf42 | Mutant | Stem-like | 0.009 |
|  | KIF2C | Mutant | Stem-like | < 0.001 |
|  | FAM63A | Mutant | Stem-like | 0.01 |
|  | TMEM25 | Mutant | Stem-like | 0.091 |
|  | RILPL2 | Mutant | Stem-like | 0.046 |
|  | CCNB2 | Mutant | Stem-like | < 0.001 |

**Table S7:** EnsEMBL Gene IDs (n=-580) of proliferation genes (see SI Materials and Methods) that were removed from the set of 83 GeneSigDB [[19](#_ENREF_19)] gene signatures that were used for class discovery with ISIS [[18](#_ENREF_18)]. After removing these genes, 2,632 genes were still used for class discovery. Further information on these genes and a one column list of these genes are available on <http://bcb.dfci.harvard.edu/~aedin/publications/>

| ENSG00000128739 | ENSG00000096696 | ENSG00000205302 | ENSG00000131408 | ENSG00000079257 |
| --- | --- | --- | --- | --- |
| ENSG00000046604 | ENSG00000017427 | ENSG00000172534 | ENSG00000170899 | ENSG00000140416 |
| ENSG00000176014 | ENSG00000119326 | ENSG00000148677 | ENSG00000170340 | ENSG00000142875 |
| ENSG00000010292 | ENSG00000177169 | ENSG00000131711 | ENSG00000065978 | ENSG00000163661 |
| ENSG00000010278 | ENSG00000241186 | ENSG00000105810 | ENSG00000143977 | ENSG00000163993 |
| ENSG00000079134 | ENSG00000111605 | ENSG00000131747 | ENSG00000090060 | ENSG00000064012 |
| ENSG00000176105 | ENSG00000106366 | ENSG00000185515 | ENSG00000184227 | ENSG00000081237 |
| ENSG00000141458 | ENSG00000176692 | ENSG00000165702 | ENSG00000115339 | ENSG00000100823 |
| ENSG00000101558 | ENSG00000168646 | ENSG00000095303 | ENSG00000162367 | ENSG00000134323 |
| ENSG00000091879 | ENSG00000000003 | ENSG00000165209 | ENSG00000143252 | ENSG00000137710 |
| ENSG00000176890 | ENSG00000138778 | ENSG00000011198 | ENSG00000118260 | ENSG00000144381 |
| ENSG00000244734 | ENSG00000090863 | ENSG00000130429 | ENSG00000116001 | ENSG00000147065 |
| ENSG00000223609 | ENSG00000006634 | ENSG00000100097 | ENSG00000197956 | ENSG00000149968 |
| ENSG00000160208 | ENSG00000072682 | ENSG00000106546 | ENSG00000004866 | ENSG00000166598 |
| ENSG00000102572 | ENSG00000041982 | ENSG00000099995 | ENSG00000159388 | ENSG00000172116 |
| ENSG00000181649 | ENSG00000140836 | ENSG00000100320 | ENSG00000100902 | ENSG00000178562 |
| ENSG00000104368 | ENSG00000136237 | ENSG00000075415 | ENSG00000100479 | ENSG00000198211 |
| ENSG00000167315 | ENSG00000165732 | ENSG00000051108 | ENSG00000057252 | ENSG00000198821 |
| ENSG00000137563 | ENSG00000109320 | ENSG00000153879 | ENSG00000163430 | ENSG00000003436 |
| ENSG00000111716 | ENSG00000152795 | ENSG00000167491 | ENSG00000213516 | ENSG00000022267 |
| ENSG00000142173 | ENSG00000168036 | ENSG00000054598 | ENSG00000079785 | ENSG00000088305 |
| ENSG00000171791 | ENSG00000151090 | ENSG00000135272 | ENSG00000196591 | ENSG00000096093 |
| ENSG00000188536 | ENSG00000136824 | ENSG00000130203 | ENSG00000097046 | ENSG00000106211 |
| ENSG00000104442 | ENSG00000112081 | ENSG00000002834 | ENSG00000125398 | ENSG00000127955 |
| ENSG00000213005 | ENSG00000187109 | ENSG00000167081 | ENSG00000116750 | ENSG00000130147 |
| ENSG00000134333 | ENSG00000110092 | ENSG00000120802 | ENSG00000181449 | ENSG00000143995 |
| ENSG00000078668 | ENSG00000105755 | ENSG00000101161 | ENSG00000181458 | ENSG00000165995 |
| ENSG00000101266 | ENSG00000100353 | ENSG00000064651 | ENSG00000025039 | ENSG00000166147 |
| ENSG00000137804 | ENSG00000171617 | ENSG00000039068 | ENSG00000143363 | ENSG00000171843 |
| ENSG00000157617 | ENSG00000008083 | ENSG00000152465 | ENSG00000198087 | ENSG00000178035 |
| ENSG00000183255 | ENSG00000123064 | ENSG00000102024 | ENSG00000114854 | ENSG00000186310 |
| ENSG00000077782 | ENSG00000146674 | ENSG00000141753 | ENSG00000169679 | ENSG00000010810 |
| ENSG00000103423 | ENSG00000183696 | ENSG00000149294 | ENSG00000214114 | ENSG00000101224 |
| ENSG00000170540 | ENSG00000122641 | ENSG00000120805 | ENSG00000135316 | ENSG00000121552 |
| ENSG00000149925 | ENSG00000137193 | ENSG00000174547 | ENSG00000152904 | ENSG00000140575 |
| ENSG00000089289 | ENSG00000166747 | ENSG00000099942 | ENSG00000196549 | ENSG00000165140 |
| ENSG00000007237 | ENSG00000135446 | ENSG00000085733 | ENSG00000124831 | ENSG00000168461 |
| ENSG00000165025 | ENSG00000087460 | ENSG00000070061 | ENSG00000178053 | ENSG00000172340 |
| ENSG00000147677 | ENSG00000146731 | ENSG00000204370 | ENSG00000155380 | ENSG00000172575 |
| ENSG00000101412 | ENSG00000106399 | ENSG00000170522 | ENSG00000136628 | ENSG00000175040 |
| ENSG00000137090 | ENSG00000106799 | ENSG00000089685 | ENSG00000050628 | ENSG00000182866 |
| ENSG00000086061 | ENSG00000171476 | ENSG00000115306 | ENSG00000138081 | ENSG00000118503 |
| ENSG00000147649 | ENSG00000145386 | ENSG00000114120 | ENSG00000153936 | ENSG00000001497 |
| ENSG00000153487 | ENSG00000118785 | ENSG00000189280 | ENSG00000112118 | ENSG00000066279 |
| ENSG00000137073 | ENSG00000132155 | ENSG00000158050 | ENSG00000188130 | ENSG00000071539 |
| ENSG00000127445 | ENSG00000091136 | ENSG00000134308 | ENSG00000100625 | ENSG00000074800 |
| ENSG00000129195 | ENSG00000168090 | ENSG00000071054 | ENSG00000162551 | ENSG00000087586 |
| ENSG00000179091 | ENSG00000148346 | ENSG00000135525 | ENSG00000081479 | ENSG00000103275 |
| ENSG00000079616 | ENSG00000149573 | ENSG00000196923 | ENSG00000163171 | ENSG00000119203 |
| ENSG00000187555 | ENSG00000150093 | ENSG00000171793 | ENSG00000188042 | ENSG00000122565 |
| ENSG00000104320 | ENSG00000131171 | ENSG00000163041 | ENSG00000108055 | ENSG00000129351 |
| ENSG00000171490 | ENSG00000076248 | ENSG00000130725 | ENSG00000123610 | ENSG00000131828 |
| ENSG00000135046 | ENSG00000087077 | ENSG00000121774 | ENSG00000113810 | ENSG00000132341 |
| ENSG00000104415 | ENSG00000130713 | ENSG00000197713 | ENSG00000105993 | ENSG00000136270 |
| ENSG00000124107 | ENSG00000164134 | ENSG00000162851 | ENSG00000073756 | ENSG00000158710 |
| ENSG00000170873 | ENSG00000163110 | ENSG00000146457 | ENSG00000091409 | ENSG00000159352 |
| ENSG00000101911 | ENSG00000164611 | ENSG00000134324 | ENSG00000013016 | ENSG00000163918 |
| ENSG00000074181 | ENSG00000134982 | ENSG00000077254 | ENSG00000075945 | ENSG00000166483 |
| ENSG00000135480 | ENSG00000184216 | ENSG00000137642 | ENSG00000058085 | ENSG00000173207 |
| ENSG00000166426 | ENSG00000112079 | ENSG00000104960 | ENSG00000127884 | ENSG00000175792 |
| ENSG00000013810 | ENSG00000077943 | ENSG00000152284 | ENSG00000114030 | ENSG00000051825 |
| ENSG00000049449 | ENSG00000011260 | ENSG00000082153 | ENSG00000021574 | ENSG00000094916 |
| ENSG00000104341 | ENSG00000085224 | ENSG00000082701 | ENSG00000081320 | ENSG00000100297 |
| ENSG00000007168 | ENSG00000087510 | ENSG00000187097 | ENSG00000070495 | ENSG00000102900 |
| ENSG00000149503 | ENSG00000120708 | ENSG00000142864 | ENSG00000144354 | ENSG00000108424 |
| ENSG00000125970 | ENSG00000181104 | ENSG00000151704 | ENSG00000170854 | ENSG00000110090 |
| ENSG00000205420 | ENSG00000186847 | ENSG00000177707 | ENSG00000112029 | ENSG00000130635 |
| ENSG00000180447 | ENSG00000164032 | ENSG00000087206 | ENSG00000112208 | ENSG00000130826 |
| ENSG00000112972 | ENSG00000196562 | ENSG00000100664 | ENSG00000088205 | ENSG00000152990 |
| ENSG00000147862 | ENSG00000147394 | ENSG00000163507 | ENSG00000144136 | ENSG00000163453 |
| ENSG00000167460 | ENSG00000185721 | ENSG00000109062 | ENSG00000169714 | ENSG00000164466 |
| ENSG00000169851 | ENSG00000164692 | ENSG00000187514 | ENSG00000170634 | ENSG00000166508 |
| ENSG00000130222 | ENSG00000141076 | ENSG00000198765 | ENSG00000115170 | ENSG00000183207 |
| ENSG00000038382 | ENSG00000147140 | ENSG00000118495 | ENSG00000143546 | ENSG00000129757 |
| ENSG00000155096 | ENSG00000198901 | ENSG00000115902 | ENSG00000143401 | ENSG00000148848 |
| ENSG00000197535 | ENSG00000136754 | ENSG00000163848 | ENSG00000134222 | ENSG00000170558 |
| ENSG00000037474 | ENSG00000148400 | ENSG00000112210 | ENSG00000114503 | ENSG00000109861 |
| ENSG00000067225 | ENSG00000168785 | ENSG00000115935 | ENSG00000076003 | ENSG00000121769 |
| ENSG00000196781 | ENSG00000102144 | ENSG00000163486 | ENSG00000109832 | ENSG00000143369 |
| ENSG00000168496 | ENSG00000081059 | ENSG00000128708 | ENSG00000082258 | ENSG00000134697 |
| ENSG00000107937 | ENSG00000005022 | ENSG00000125630 | ENSG00000155130 | ENSG00000110195 |
| ENSG00000075618 | ENSG00000130702 | ENSG00000100842 | ENSG00000117505 | ENSG00000115310 |
| ENSG00000164754 | ENSG00000086827 | ENSG00000139970 | ENSG00000163191 | ENSG00000116062 |
| ENSG00000131051 | ENSG00000197977 | ENSG00000135775 | ENSG00000159377 | ENSG00000187735 |
| ENSG00000101335 | ENSG00000125686 | ENSG00000134954 | ENSG00000117569 | ENSG00000188419 |
| ENSG00000176102 | ENSG00000127184 | ENSG00000135829 | ENSG00000188910 | ENSG00000034063 |
| ENSG00000125977 | ENSG00000110880 | ENSG00000148798 | ENSG00000169251 | ENSG00000072571 |
| ENSG00000149257 | ENSG00000171634 | ENSG00000116560 | ENSG00000176887 | ENSG00000112576 |
| ENSG00000153044 | ENSG00000119402 | ENSG00000143033 | ENSG00000162704 | ENSG00000112984 |
| ENSG00000147202 | ENSG00000197694 | ENSG00000119953 | ENSG00000162909 | ENSG00000113387 |
| ENSG00000156110 | ENSG00000139289 | ENSG00000148835 | ENSG00000114491 | ENSG00000118985 |
| ENSG00000170275 | ENSG00000110955 | ENSG00000166165 | ENSG00000049323 | ENSG00000138160 |
| ENSG00000143476 | ENSG00000163931 | ENSG00000160752 | ENSG00000095637 | ENSG00000111711 |
| ENSG00000152601 | ENSG00000165678 | ENSG00000064393 | ENSG00000133169 | ENSG00000136861 |
| ENSG00000156970 | ENSG00000166741 | ENSG00000087842 | ENSG00000116157 | ENSG00000147044 |
| ENSG00000162613 | ENSG00000172059 | ENSG00000102145 | ENSG00000162928 | ENSG00000176697 |
| ENSG00000165304 | ENSG00000172216 | ENSG00000123080 | ENSG00000182481 | ENSG00000184588 |
| ENSG00000170312 | ENSG00000173221 | ENSG00000132432 | ENSG00000099204 | ENSG00000068305 |
| ENSG00000170345 | ENSG00000174307 | ENSG00000111581 | ENSG00000108788 | ENSG00000088325 |
| ENSG00000171848 | ENSG00000177469 | ENSG00000123297 | ENSG00000109846 | ENSG00000122786 |
| ENSG00000175305 | ENSG00000185201 | ENSG00000135679 | ENSG00000115361 | ENSG00000157456 |
| ENSG00000188229 | ENSG00000185825 | ENSG00000093000 | ENSG00000116016 | ENSG00000182492 |
| ENSG00000211456 | ENSG00000186868 | ENSG00000107290 | ENSG00000116096 | ENSG00000196924 |
| ENSG00000020426 | ENSG00000189221 | ENSG00000115966 | ENSG00000124762 | ENSG00000056345 |
| ENSG00000117676 | ENSG00000205336 | ENSG00000128590 | ENSG00000125037 | ENSG00000125257 |
| ENSG00000135318 | ENSG00000115364 | ENSG00000137947 | ENSG00000130164 | ENSG00000164171 |
| ENSG00000106278 | ENSG00000035403 | ENSG00000159479 | ENSG00000132541 | ENSG00000184500 |
| ENSG00000060718 | ENSG00000072778 | ENSG00000165030 | ENSG00000134480 | ENSG00000017260 |
| ENSG00000163235 | ENSG00000131504 | ENSG00000168268 | ENSG00000138382 | ENSG00000117155 |
| ENSG00000087088 | ENSG00000133606 | ENSG00000172071 | ENSG00000140284 | ENSG00000104549 |
| ENSG00000125755 | ENSG00000133997 | ENSG00000175197 | ENSG00000140941 | ENSG00000109929 |
| ENSG00000145194 | ENSG00000009413 | ENSG00000061918 | ENSG00000151929 | ENSG00000147383 |
| ENSG00000214706 | ENSG00000101158 | ENSG00000163659 | ENSG00000160307 | ENSG00000149809 |
| ENSG00000086062 | ENSG00000137497 | ENSG00000077150 | ENSG00000006468 | ENSG00000173402 |
| ENSG00000104725 | ENSG00000138772 | ENSG00000115904 | ENSG00000068366 | ENSG00000113083 |
| ENSG00000118777 | ENSG00000159131 | ENSG00000121879 | ENSG00000160712 | ENSG00000138095 |
